# Sesquiterpene Cyclase BcBOT2 Promotes the Unprecedented Wagner-Meerwein Rearrangement of the Methoxy Group

**DOI:** 10.1021/jacs.4c03386

**Published:** 2024-06-18

**Authors:** Malte Moeller, Dipendu Dhar, Gerald Dräger, Mikail Özbasi, Henry Struwe, Maik Wildhagen, Mehdi D. Davari, Sascha Beutel, Andreas Kirschning

**Affiliations:** † Institute of Organic Chemistry, Leibniz Universität Hannover, Schneiderberg 1B, 30167 Hannover, Germany; ‡ Department of Bioorganic Chemistry, Leibniz Institute of Plant Biochemistry (IPB), Weinberg 3, 06120 Halle, Germany; § Institute for Technical Chemistry, Leibniz University Hannover, Callinstr. 5, 30167 Hannover, Germany; ∥ Uppsala Biomedical Center (BMC), University Uppsala, Husargatan 3, 752 37 Uppsala, Sweden

## Abstract

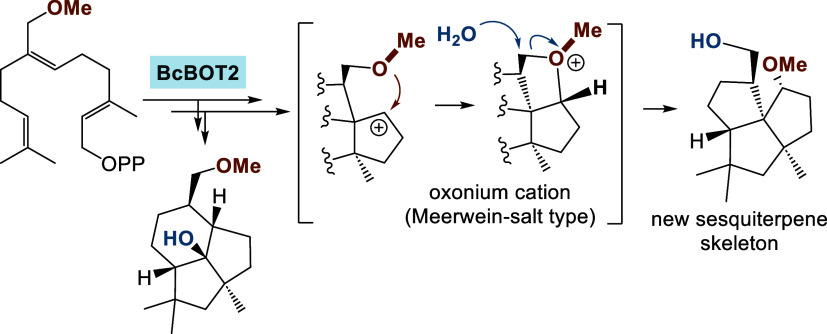

Presilphiperfolan-8β-ol synthase (BcBOT2), a substrate-promiscuous sesquiterpene cyclase (STC) of fungal origin, is capable of converting two new farnesyl pyrophosphate (FPP) derivatives modified at C7 of farnesyl pyrophosphate (FPP) bearing either a hydroxymethyl group or a methoxymethyl group. These substrates were chosen based on a computationally generated model. Biotransformations yielded five new oxygenated terpenoids. Remarkably, the formation of one of these tricyclic products can only be explained by a cationically induced migration of the methoxy group, presumably via a Meerwein-salt intermediate, unprecedented in synthetic chemistry and biosynthesis. The results show the great principle and general potential of terpene cyclases for mechanistic studies of unusual cation chemistry and for the creation of new terpene skeletons.

## Introduction

Sesquiterpene cyclases (STCs), like other terpene cyclases (TCs), use linear, unsaturated, methyl-branched precursors activated as diphosphate monoesters. In the case of STCs, this is farnesyl pyrophosphate (FPP **1**), and initially, a more or less prominent cationic allyl intermediate is formed that can react further with distant alkenes to form (oligo)carbocyclic products.^1^ In recent years, the substrate promiscuity of sesquiterpene cyclases has become an active field of research. In this context, oxygenated FPP derivatives are attractive unnatural candidates,^2−4^ also because oxyfunctionalized mono- and sesquiterpenes are widely used in the flavor and fragrance industry due to favorable olfactoric properties compared to the hydrocarbons initially formed by STCs.^5^

**Scheme 1:**
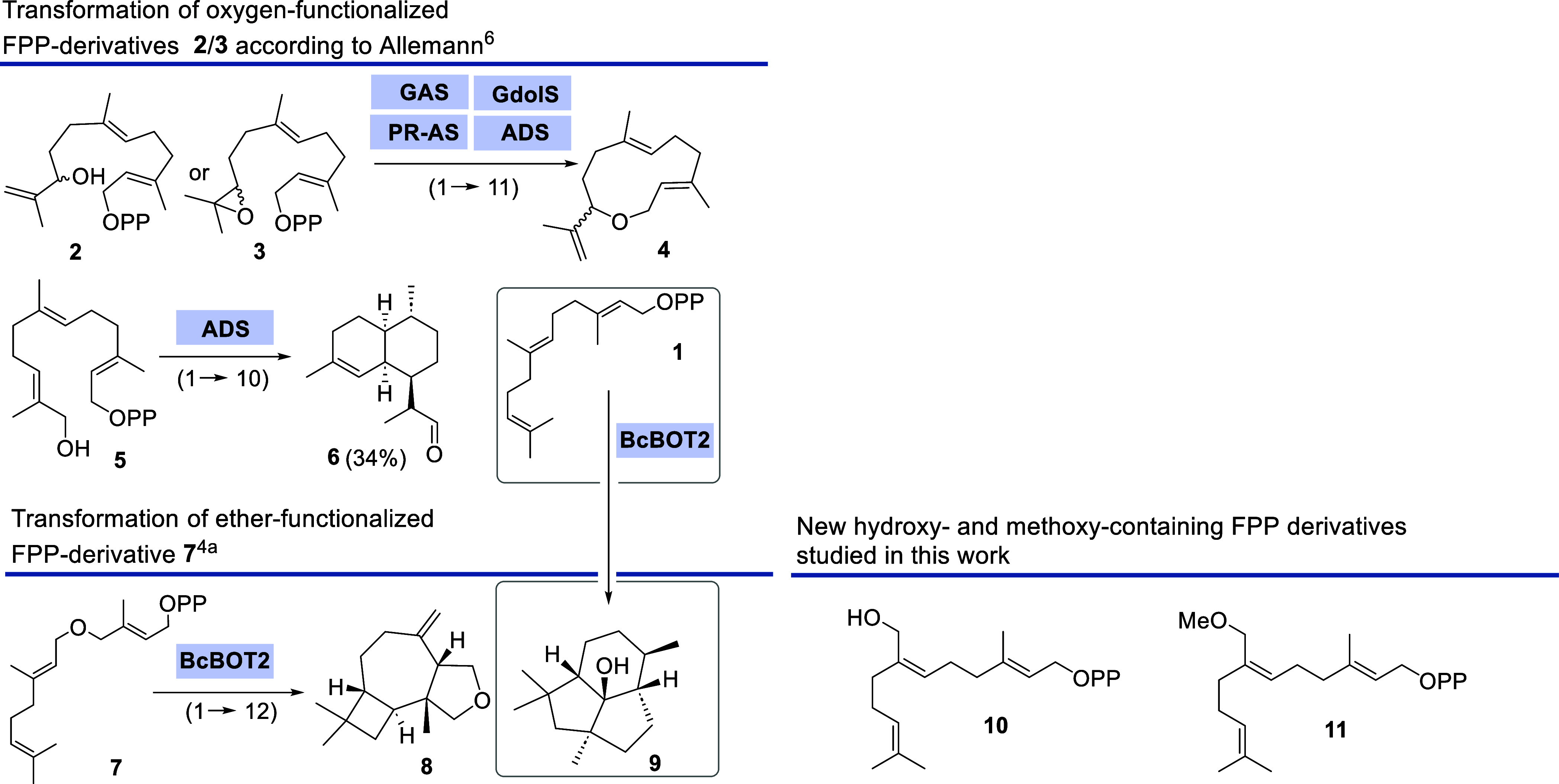
Structure of FPP **1,** STC-Promoted Conversions of Oxygenated FPP-Derivatives **2**, **3**, and **5**, of FPP Ether Derivative **7** and Structures of FPP Derivatives **10** and **11** Studied in This Work [OPP = OP_2_O_6_H_3_ (Protonated Form)]

Allemann and co-workers chose two related oxygen-functionalized FPP derivatives, the allyl alcohol **2** and the oxirane **3,** and investigated their potential as unnatural substrates for different STCs (Scheme 1).^6^ In particular, germacrene A synthase from *Solidago canadensis* (GAS) and the germacradiene-4-ol synthase (GdolS) showed promiscuity toward these two substrates, and the product-converging transformations yielded the same macrocyclization product **4**.^6^ Acceptance for FPP **2** and **3** derivatives was also found for aristolochene synthase from *Penicilium roqueforti* (PR-AS) and amorphadiene synthase (ADS), although with variable and lower yields. A remarkable preparative application of using non-natural FPP derivatives was reported by the same group. The biotransformation of 12-hydroxy-FPP **5** with ADS yielded dihydroartemisinic aldehyde (**6**, DHAAI) as a suitable precursor for accessing artemisinin.^7^

Also, ether and thioether FPP derivatives were harnessed and transformed, and among them, FPP ether **7**(2) proved to be a particularly interesting substrate for STCs. With presilphiperfolan-8β-ol synthase (BcBOT2), a fungal sesquiterpene cyclase from *Botrytis cinerea*,^8^ the biotransformation yielded the tricyclic terpenoid **8**, which displays a significantly altered backbone to the natural cyclization product presilphiperfolan-8β-ol **9,** and this new oxysesquiterpene exerts a similar olfactory profile to the sesquiterpene rotundone.^4a^

The examples described in Scheme 1 show that the initially formed cationic allyl species either allows one to create a C–C bond during macrocyclization or can alternatively be captured by an oxygen nucleophile to form a C–O bond. In essence, TCs show a much broader synthetic potential than is commonly assumed.

## Results and Discussion

As part of a larger research program, we now disclose our findings on the substrate promiscuity of the fungal sesquiterpene cyclase BcBOT2 using oxygenated FPP derivatives **10** and **11**. In the list of sesquiterpene cyclases whose substrate promiscuity we have tested in the past, BcBOT2 stands out, both in terms of substrate promiscuity as well as in terms of its efficiency, which allows preparative scalable transformations to be performed with relative ease.

**Figure 1:**
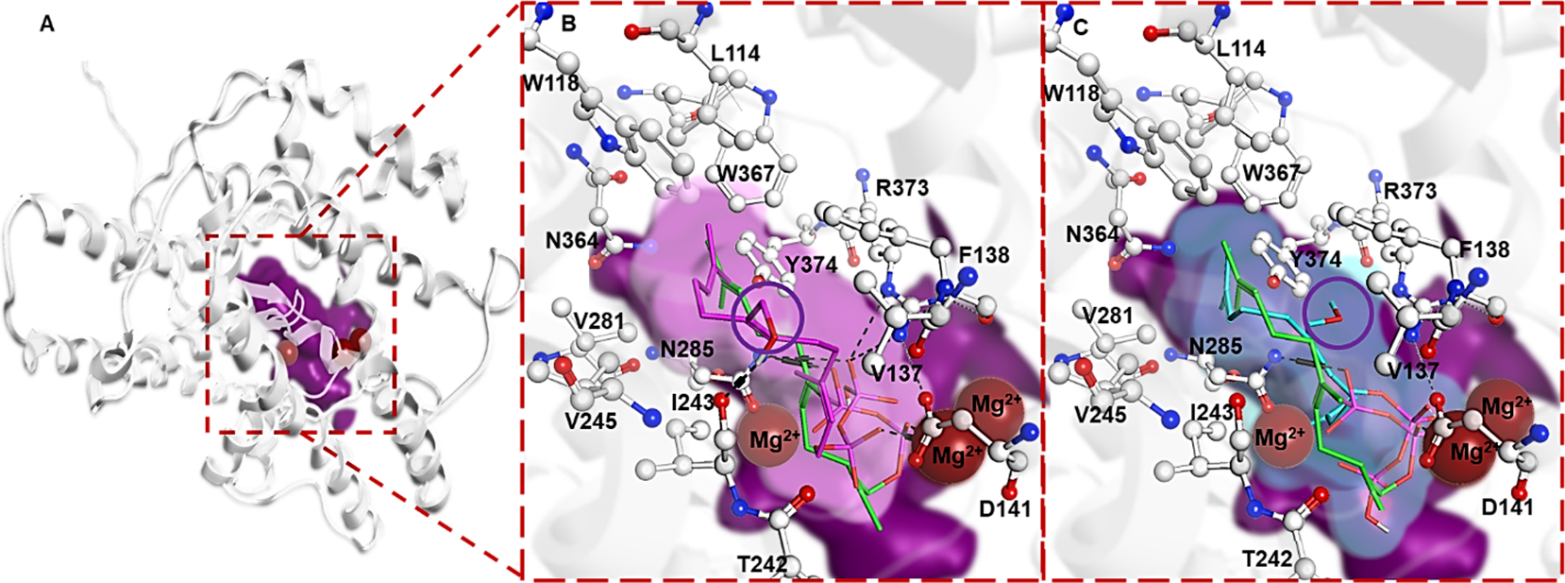
Structural model of BcBOT2 with FPP **1** and new derivatives **10** and **11**. (A) Model is displayed graphically in gray, with the shaded area representing the volume occupied by the three superimposed substrates, while the triad of Mg^2+^ ions is shown as red spheres. The purple shaded area shows the total volume of the binding pocket, while the magenta and cyan shaded areas indicate the volume of the hydroxyl and methoxy derivatives of FPP **10** and **11**, respectively. The substituted group in each of the derivatives is indicated by a purple circle. The FPP molecule is shown in the form of green sticks. Close-up of the binding pocket with FPP derivatives **10** (B) in cyan and **11** (C) in magenta sticks superimposed on FPP **1,** respectively.

The choice of these substrates **10** and **11** was based on structural rationale. During the course of these studies, the first structural data on BcBOT2 were reported.^9^ Furthermore, we developed a structural model for BcBOT2 which was predicted using AlphaFold2.^10^ It was combined with an energy minimization using the AMBER14 force field, the overall RMSD of the Alphafold model used, and the newly reported structure being <1.72 Å (details are found in SI).^10^ The cavity calculations revealed that the total volume for the active site cavity of BcBOT2 where the substrate binding occurs is 567.8 Å^3^. As a result, we performed additional molecular docking simulations to assess whether additional substituents could be added to the FPP and whether the new derivatives would still fit into the active site of BcBOT2. Specifically, as we planned to attach an additional hydroxy and a methoxy group, respectively, at the central methyl group at C7 of FPP **1**, we calculated the total volume for these two derivatives and found that it was 350 and 369.5 Å^3^, respectively (Figure 1; details are found in the SI). Given these results, we assumed that the free space should be sufficient for positioning the additional hydroxy and methoxy groups in the active site of BcBOT2. These findings served as the starting point for the present study which was initiated with the synthesis of the two FPP derivatives **10** and **11**.

**Scheme 2:**
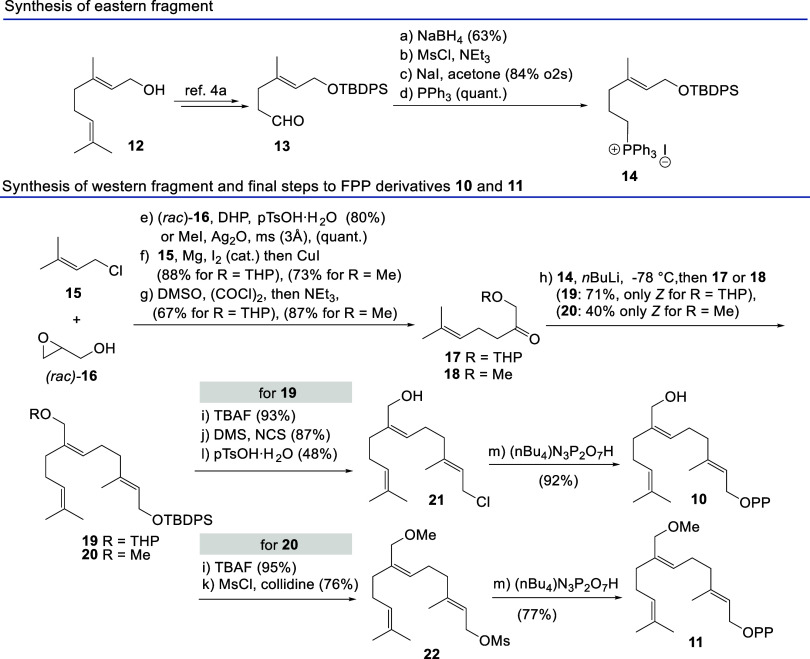
Syntheses of FPP Derivatives **10** and **11**

The syntheses for the two FPP derivatives **10** and **11** were carried out quite analogously, except that the two pathways separated at an early stage, namely, when the methoxy group was established (Scheme 2). The western and eastern fragments were merged by a highly *Z*-selective Wittig olefination of the P-ylid derived from phosphonium salt **14** and ketones **17** and **18**,^11^ respectively.

The synthesis of fragment **14** commenced from geraniol **12,** which was transformed to aldehyde **13** by a known procedure.^4a^ This was converted to **14** via the alcohol, the mesylate, and hence the intermediate iodide. The syntheses of the two western fragments **17** and **18** utilize prenyl chloride **15** and *rac*-glycidol **16**. The alcohol group was either protected as THP-acetal or *O*-methylated before being reacted with the Grignard reagent derived from chloride **15**.

Both Wittig olefinations that followed proceeded with excellent *Z*-selectivity and yielded TBDPS-ether **19** and **20**. At this stage, the diphosphate moiety was introduced after the removal of the TBDPS protection following a protocol that either proceeds via the intermediate allyl chloride or allyl mesylate.^12^

Next, the enzyme BcBOT2 was cloned and expressed in *E. coli* as detailed in the SI. To determine enzyme activity and substrate tolerance, in vitro enzyme assays were conducted with FPP **1** and derivatives **10** and **11** (500 μL scale, 0.1 g/L BcBOT2, 37 °C, 0.5 h). The outcome of biotransformations of FPP derivatives **10** and **11** with BcBOT2 is summarized in Table 1, revealing the formation of several products.

**Table 1:** Overview of Biotransformations of BcBOT2 Using Diphosphates **10** and **11**^a^

diphosphate	products *m*/*z*	retention index (RI)
**10**	220^b^	1591
**11**	major:^b^ 2 × 252	1832, 1844
	minor: 3 × 234	1602, 1619, 1639

a Determined by GC/MS; *m*/*z* = 220 or 234 final deprotonation; *m*/*z* = 252 trapping of water.

b The structures of these products are reported in Scheme 3.

Indeed, transformations with STCs usually do not yield only one product; this is also true for BcBOT2 with the natural substrate FPP **1** which yields presilphiperfolan-8β-ol (**9**) as major product but also β-caryophyllene, caryophyllene oxide, β-elemene, germacrene A, presilphiperfol-7-ene, (*E*)-β-farnesene, and (*E*)-nerolidol (GC: overall A[%] < 5%).^10^ However, the major product usually predominates to such an extent that it is published alone.

Mass spectrometric analysis of the crude product provides a rapid overview of the product spectrum, including whether the individual product results from the terminal elimination of a proton or the reaction of the final carbocation with the nucleophile water. Incubation of BcBOT2 in the presence of diphosphate **10** only provided one product while diphosphate **11** was transformed into five well-detectable new sesquiterpene derivatives.

Our screening program also included other sesquiterpene cyclases, namely, (+)-caryolan-1-ol synthase (GcoA), viridiflorene synthase (Tps32), epi-isozizaene synthase (Cyc1), and vetispiradiene synthase (Hvs1).^13^ In most cases, conversion in the presence of FPP derivatives **10** and **11** resulted in new products, as judged by GC-MS analysis. However, a more detailed analysis revealed that only very small amounts were formed, which are not sufficient to practically upscale these transformations. These results support our previous observations on the uniqueness of BcBOT2.

For conducting semipreparative reactions in the following, for the transformations of BcBOT2 with oxygenated FPP derivatives **10** and **11,** the conditions were slightly changed (1 mM scale, 0.1 g/L BcBOT2, 37 °C, 24 h). A sufficient amount of material was collected for the purification and structural elucidation of the three main products, namely, the tricyclic oxygenated sesquiterpenes **23**, **25,** and **26** (Scheme 3, top).

**Scheme 3:**
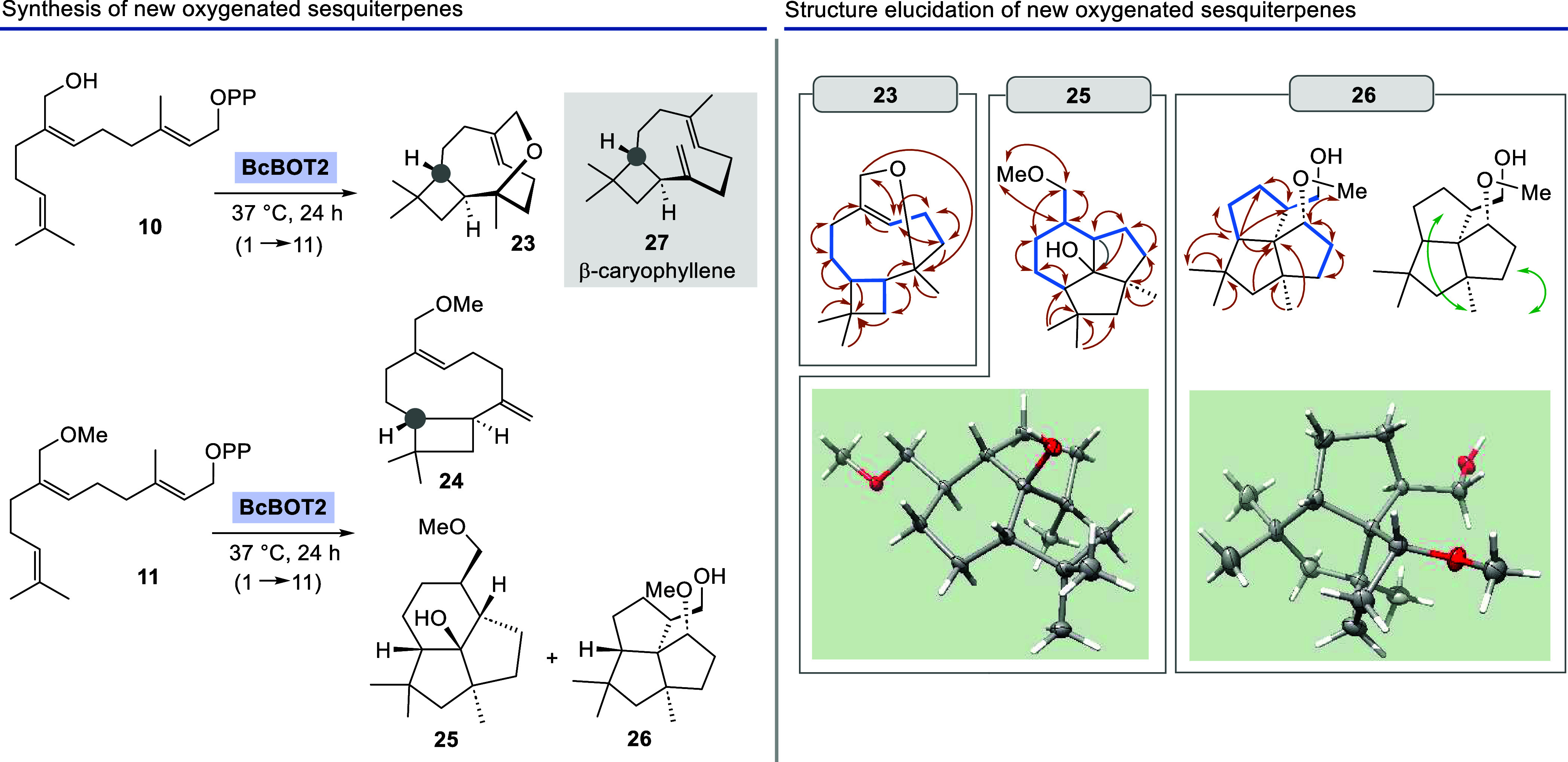
Left: Transformations of FPP Derivatives **10** and **11** by BcBOT2 (Positions Marked in Grey Are Reference Carbon Atoms for the Absolute Stereochemistry); Right: Details on the Structure Analysis of **23**, **25** and **26** Key correlations of HMBC-spectra (orange arrows), H,H–COSY correlations (blue bonds), key NOEs (areen arrows), and crystal structures as analyzed by X-ray (the absolute configuration was determined via *Anormalous Dispersion* according to ref (14)).

**Scheme 4:**
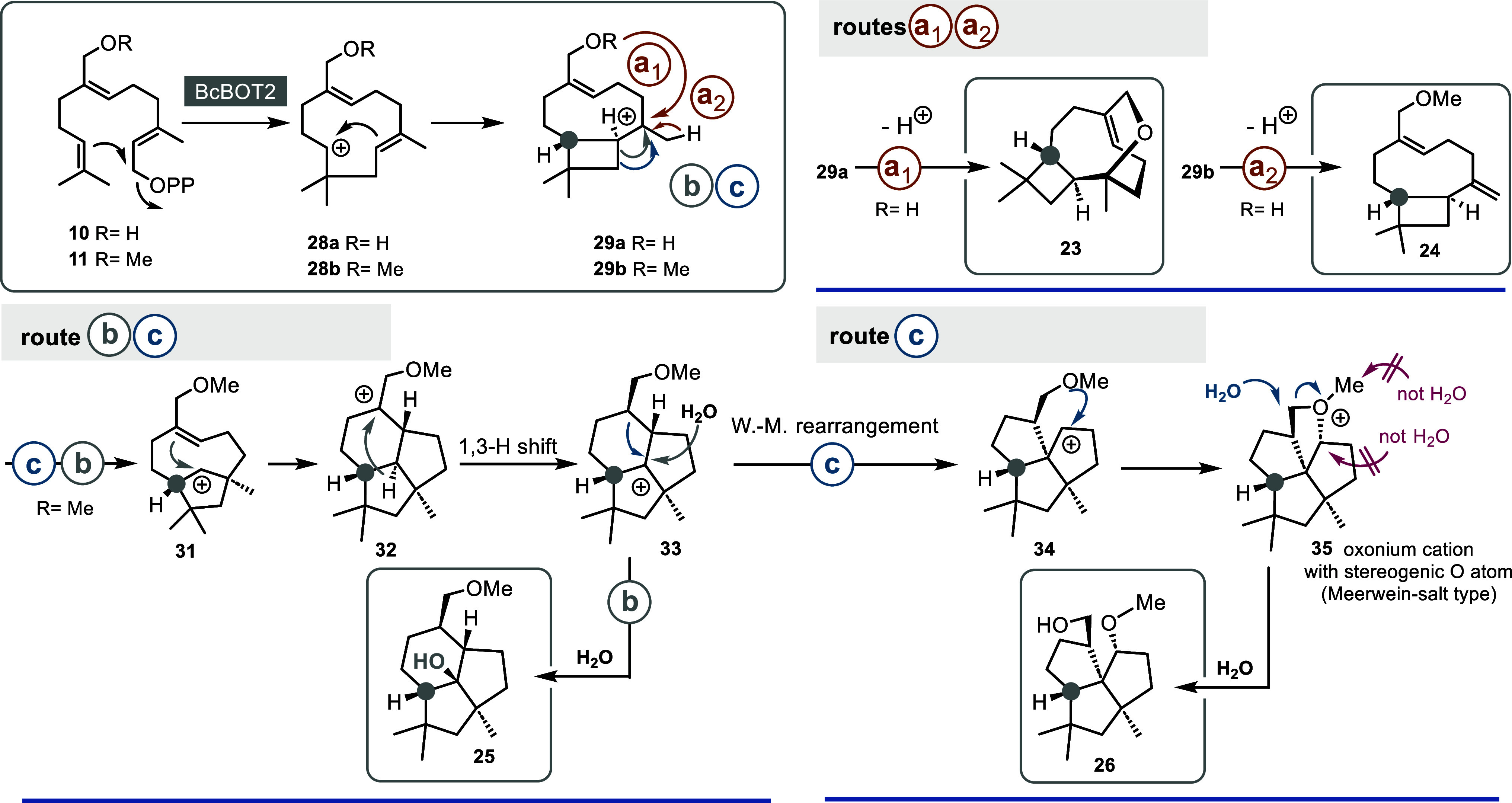
Mechanistic Considerations on the Formation of New Oxygenated Sesquiterpenoids **23**–**26** Routes A–C; carbon atom marked in grey acts as a reference point for the absolute stereochemistry of new products.

The constitutions of these new products were elucidated by using various methods of NMR spectroscopy. In particular, the COSY and HMBC correlations played an important role, which were additionally complemented by the determination of selected NOE data (Scheme 3, bottom). These provided information about the preferred conformation of the macrocycles and thus additional information about the relative stereochemistry. The absolute stereochemistry was first determined by assuming a common mechanism for the formation of the four biotransformation products **23**-**26**. The labeled stereogenic center serves as a reference, which is formed right at the beginning of the cation cascade and which is identical to the one in presilphiperfolan-8β-ol (**9**) at this position (Scheme 4).

The following points list key elements of the structure elucidation of oxo-bridged sesquiterpene **23** by NMR analysis. A doublet at δ = 4 ppm indicates the presence of only one CH_2_–group next to a heteroatom. Following its coupling partners in the HMBC spectrum, it was clear that a macrocyclic ether with a neighboring quaternary carbon atom must have formed. The cyclobutane ring was resolved by identifying the downfield shifted hydrogen atoms of the methylene group. The upper part of **23** was resolved by combining COSY- with HMBC-correlations starting with the signal of the double bond proton. The carbon backbone of oxo-bridged product **23** resembles that of the natural sesquiterpene β-caryophyllene **27**.

The alkene and two geminal methyl groups served as the starting point for the structural elucidation of **24** by NMR spectroscopy. COSY, HSQC, DEPT135, and HMBC NMR spectra were primarily used for the assignment and thus for the structure elucidation. The characteristic signals for the 1,1-disubstituted exocyclic double bond (δ = 4.97 and 4.79 ppm) and the presence of the cyclobutane methine groups support the proposed structure. The detection of two sets of signals for the 1,1-disubstituted olefin protons indicates the presence of two different conformers as the GC-MS analysis does not reveal any significant impurities.

The structural elucidation of product **25** was facilitated by direct comparison with the NMR data of presilphiperfolan-8β-ol (**9**). While the NMR data for the lower part of the two sesquiterpenes are almost identical, a new CH_2_ group was identified resulting from the presence of the extra OMe group in FPP derivative **11**. This additionally led to slight downward shifts of the ^1^H signals in the neighborhood compared to **9**.

The position of the methoxy group in **26** was first determined by HSQC and HMBC spectra and revealed that it is unexpectedly not attached to a methylene group as in starting FPP derivative **11** but rather bound to a tertiary carbon atom. Obviously, the methoxy group must have undergone migration. Gratifyingly, efforts to crystallize terpenoids **25** and **26** were successful, and X-ray analyses provided final structural proof. These analyses also confirmed the absolute stereochemistry of these new oxygenated terpenoids.

A closer inspection of the backbones of the products reveals that they must have been formed by a complex cationic cascade. This is especially true for tricyclic oxygenated sesquiterpene **26**, which has some surprises to offer. Mechanistically, several migrations must have taken place here, and most surprisingly, the methoxy group must have changed its position with respect to the carbon backbone. Given that the caryophyllene cation is an important intermediate on the pathway to presilphiperfolan-8β-ol (**9**), we propose that the analogous cations **29a**,**b** (from the humulyl-type cations **28a**,**b**) are mechanistically central intermediates from which two main pathways branch mechanistically (a and b, c) that yield the four products **23**–**26** (Scheme 4). For accessing terpenoid **23,** cation **29a** is internally trapped by the hydroxyl group (route a_1_) which creates the oxymethylene bridge which upon deprotonation furnishes sesquiterpene derivative **23**. Likewise, the methoxy FPP derivative **11** yields **24** via a similar sequence except that cation **29b** is deprotonated in the final step (a_2_).

Product **25** represents the methoxy derivative of presilphiperfolan-8β-ol (**9**) and will therefore be formed according to route b by following the mechanism discussed in the literature.^4c^ This route starts with ring expansion of the cyclobutane ring by Wagner-Meerwein rearrangement at the stage of cation **29b**. The newly formed cation **31** undergoes another ring closure, and the new carbocation **32** then presumably performs a 1,3-hydride shift until the tertiary cation **33** is intercepted by water and, consequently, **25** is formed.

The proposed formation of terpenoid **26** also starts from intermediate **29b** and follows pathway b up to the point of intermediate cation **33**. From there, we propose a ring contraction which yields intermediate **34** with a triquinane skeleton. The carbocation and the methoxy group are positioned close in space so that by capturing the cation, the unusual intermediate oxonium ion **35** is formed. Attack by water at the carbon atom where the methoxy group was originally located finally yields terpenoid **26**. The oxonium salt **35** represents a complex version of what is called a Meerwein-salt. These are strong alkylating agents.^15^

**Figure 2:**
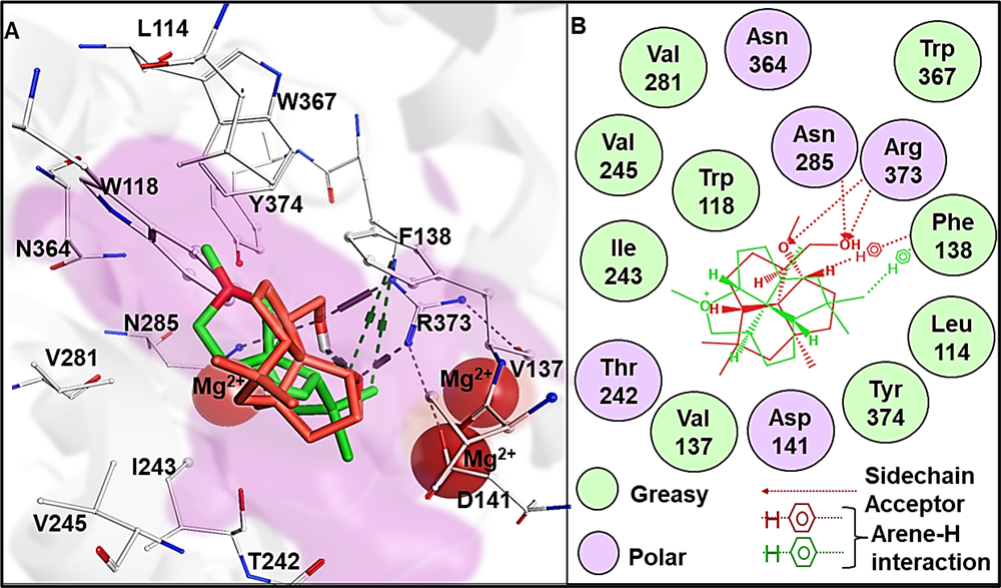
(A) Closeup of the active site of BcBOT2 with the top scoring catalytically competent binding poses from the molecular docking of the reaction intermediate **35** (green) and the product **26** (red) from proposed pathway c, the active site pocket (violet) with interacting residues (white stick and balls) along with the triad of Mg^2+^ (red spheres) also shown. Side chain acceptor and arene-H interaction depicted as black and green dotted lines, respectively.^19^ (B) 2D overlay of the ligand interaction diagram of **36** (green) and **26** (red) showing their interactions with the neighboring residues; legend for the ligand interaction diagram shown below. A complete depiction of all the intermediates leading to the formation of **26** by proposed pathway c can be found in SI.

In fact, mechanistically, this sequence represents a 1,4-Wagner Meerwein rearrangement. Remarkably, the trialkyloxonium ion **35** bears a stereogenic oxygen formed in a diastereoselective manner. Smith and co-workers only recently presented the first report of a chiral oxonium cation. Its architecture is based on a stable triaryloxonium ion which is embedded in a helical environment.^16^ The molecular docking of the intermediates in proposed pathway c aligns with findings from previous works on terpenoid cyclases including BcBOT2^1−4^ which has shown that the carbocations formed in the intermediates experience stabilization through π-system interactions mainly through phenylalanine (Phe), tyrosine (Tyr), and tryptophan (Trp) amino acid residues. As can be seen in Figure 2, such an interaction is also found here with Phe138. An additional interaction between **26** and adjacent Asn285 and Arg373 likely favors the formation of **26**.

In silico site saturation mutagenesis of these two positions was performed to investigate this hypothesis. All of the variants of Arg373 were predicted not to be stabilizing. The stable variants for Asn285 (N285R, N285F, N285W, and N285Y), all had the critical aryl interaction with Phe138, but the additional interaction was replaced with adjacent Tyr374 for most of **26** (details are found in SI).^17^ This supports our initial assumption and finds the interaction with Arg373 specifically to be important in the formation of **26**.

**Scheme 5:**
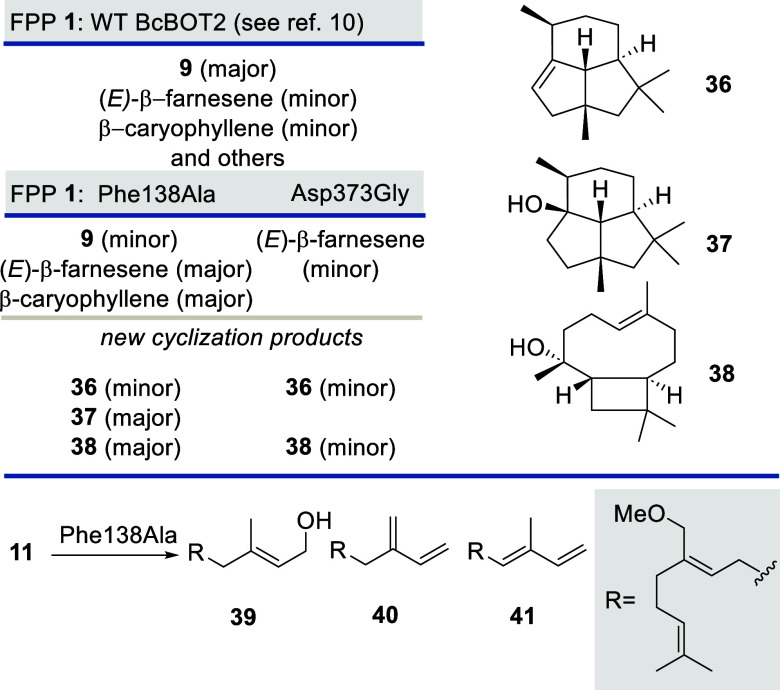
Results of Transformations of FPP **1** and FPP Derivative **11** Using BcBOT2 Variants Phe138Ala and Asp373Gly Further details using **1** as a substrate are reported in ref (10).

To corroborate the outcome of the computational studies, we carried out additional biotransformations with two BcBOT2 variants. Specifically, we chose the two Phe138Ala and Arg373Gly variants, which upon incubation with FPP **1** provide new sesquiterpenes **36**–**38** along with sesquiterpenes also produced by the wildtype enzyme (Scheme 5, top).^10^ When the FPP derivative **11** was exposed to the Arg373Gly variant, complete suppression of product formation was observed (see the SI). This also includes the cyclization products **25** and **26**, as judged by GC-MS. In contrast to this observation, the Phe138Ala variant was able to transform FPP derivative **11**, but instead of terpenoids **25** and **26**, the formation of the “hydrolysis” product **39** and two minor “elimination” products **40** and **41** were found (Scheme 5, bottom). Both results clearly demonstrate the active involvement of Phe138 and Arg373 in promoting transformations with FPP derivative **11** and the generation of terpenoids **25** and **26**.

Carbocation-induced migration of methoxy groups is virtually unknown. In rare cases, migrations of anomeric methoxy groups in methyl pyranosides toward ring carbon atoms whose alcohol groups are modified as esters with marked leaving group quality have been reported.^18^ The resulting oxocarbenium ions at the anomeric center are finally captured by a nucleophile such as water. However, these cases cannot fully be compared with the present case as the oxygen atom of the pyran ring exerts a strong stabilizing effect, which supports such migrations. As such, the migration process described here is unique because it takes place with an isolated methoxy group. It is probably made possible by the protein and its preformed 3D space. This controls the position of the final incoming nucleophile (here water) and the course of the last steps of the cationic cascade, including the methoxy migration in a completely controlled way.

**Figure 3:**
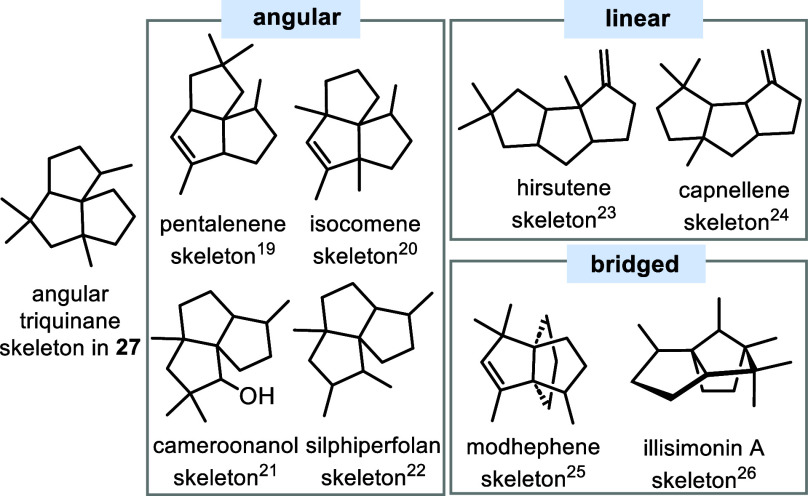
Terpene-based triquinane skeletons (angular, linear, bridged) and comparison with the new triquinane backbone **27** reported here (illisimonin A is a highly oxygenated sesquiterpene; for better clarity, only the carbon backbone is shown).

While the carbon skeleton of oxo-bridged terpene **23** and the methoxy terpenoid **24** are akin to that of caryophyllene **27**, the relationship of the backbone terpene **26** can be assigned to triquinanes. Tricyclic terpenes composed of three annulated cyclopentane rings are well-known. Angular,^19−22^ linear,^23,24^ and bridged^25,26^ members are listed in Figure 3 and compared, and they reveal that **26** belongs to the group of angular triquinanes. However, the methylation pattern in **26** differs from that found in the known angular triquinane terpenes pentalenan, isocoman, cameroonan, and silphiperfolan carbon backbones.

## Conclusions

In summary, we report the first example of methoxy migration via an oxonium intermediate mediated by the sesquiterpene cyclase BcBOT2, a sesquiterpene cyclase with unique substrate promiscuity, a process not previously observed in chemical environments. So far, oxonium ions have been proposed as temporary intermediates in a series of biosynthetic^27^ and synthetic transformations,^28,4a^ usually upon interference of oxirane, furan, or pyran oxygen atoms commonly on nascent bromine or selenonium cations after activation of alkenes.

These results document that not only do small changes in the amino acid composition located in the active site pocket of sesquiterpene cyclases exert large effects on cationic cascade sequences^13^ but also in native STCs small structural changes in the FPP substrate lead to novel cationic sequences. We show that STCs are able to handle oxonium cations in a highly controlled manner, which was previously unknown, because the natural substrate FPP **1** leads to cationic intermediates in the absence of an oxygen atom. Therefore, the question of whether or how terpene cyclase handles oxonium ions could have never arisen before. We believe that the 3D space formed by the protein in terpene cyclases can thus serve as a chemical laboratory in which a large variety of cationic sequence scenarios become feasible, much more widely than was originally “planned” by Nature. This unique catalytic space should be exploited by chemists to study the scopes of cationic cascade chemistry in confined environments. These may eventually be transferable to chemically designed, catalytically active 3D spaces such as those elegantly described by Tiefenbacher and co-workers in recent years.^29^

## Supporting Information Available

Experimental procedures, characterizations, and analytical data of new compounds, X-ray diffraction data for **25** and **26**, and NMR spectra (PDF)

## ABBREVIATIONS

TBDPS: *tert*-butyldiphenylsilyl
Ms: methylsulfonyl
DHP: dihydropyrane
THP: tetrahydropyranyl
*p*TsOH: *p*-tosylsulfonyl
TBAF: *tetra*-*n*-butylammonium fluoride
DMS: dimethylsulfide
NCS: *N*-chlorosuccinimide
DMSO: dimethyl sulfoxide
