# Supplementary material for: Sesquiterpene Cyclase BcBOT2 Promotes the Unprecedented Wagner-Meerwein Rearrangement of the Methoxy Group
Source: J Am Chem Soc. 2024 Jun 18;146(26):17838–46. doi: 10.1021/jacs.4c03386 (PMC11228982; doi:10.1021/jacs.4c03386)
Supplement: Supplementary file 1 — ja4c03386_si_001.pdf [file ja4c03386_si_001.pdf]

The sesquiterpene cyclase BcBOT2 promotes the unprecedented Wagner-Meerwein rearrangement of the methoxy group

Malte Moeller [a], Dipendu Dhar[b], Gerald Dräger[a], Mikail Özbasi[a], Henry Struwe[a], Maik Wildhagen[c], Mehdi D. Davari[b], Sascha Beutel[c], Andreas Kirschning[*a,d]*

[a] Institute of Organic Chemistry, Leibniz University Hannover, Schneiderberg 1B, 30167 Hannover, Germany

[b] Department of Bioorganic Chemistry, Leibniz Institute of Plant Biochemistry Weinberg 3, 06120 Halle, Germany

[c] Institute of Technical Chemistry, Leibniz University Hannover, Callinstr 5, 30167 Hannover, Germany

[d] Uppsala Biomedical Center (BMC), University Uppsala, Husargatan 3, 752 37 Uppsala, Sweden.

1. Materials and methods
1.1 General information
1.2 Genes, strains, plasmids, expression and growth conditions
1.3 Protein purification
1.4 Analytical enzyme tests

2. Chemical Syntheses
2.1 Synthesis of tris(tetra-*n*-butylammonium)hydrogenpyrophosphate trihydrate **S1**
2.2 Synthesis of farnesylpyrophosphate derivative **10**
2.3 Synthesis of farnesylpyrophosphate derivative **11**

3. Biotransformations with unnatural farnesyl pyrophosphates on semi-preparative scale
3.1 Structure elucidations of new biotransformation product **23**
3.2 Structure elucidations of new biotransformation products **24-26**
3.3 Structure elucidations of new biotransformation products **40** and **41**

4. Molecular Modelling

5. Xray analysis

5.1 Xray analysis of 25

5.2 Xray analysis of 26

6. References (supporting information)

Attachments

A. GC chromatograms and MS spectra of new terpenoids
B. Copies NMR spectra of new terpenoids
C. Copies of ^{1}H- and ^{13}C-NMR spectra (chemical synthesis)
D. Copies of ^{31}P-NMR spectra (chemical synthesis)

1. Materials and methods

1.1 General information

^1H NMR spectra were recorded at 400 MHz with a BRUKER Avance-400 and BRUKER Ascend-400 as well as at 500 MHz with a BRUKER DRX-500 spectrometer or at 600 MHz with a BRUKER Ascend-600 or BRUKER Avance III-600 at 298K or 255K for low temperature NMR measurements. ^{13}C NMR spectra were recorded at 100 MHz with a BRUKER Avance-400 and BRUKER Ascend-400 or at 125 MHz with a BRUKER DRX-500 or at 151 MHz with a BRUKER Ascend-600/ Avance III-600 instrument. ^{31}P NMR spectra were recorded at 162 MHz with a BRUKER Avance-400 and BRUKER Ascend-400 or at 202 MHz with a BRUKER DRX-500 instrument. Multiplicities are described using the following abbreviations: s = singlet, d = doublet, t = triplet, q = quartet, sex = sextet, m = multiplet, b = broad. Chemical shift values of ^1H and ^{13}C NMR spectra are commonly reported in ppm relative to residual solvent signal as internal standard. The multiplicities refer to the resonances in the off-resonance decoupled spectra and were elucidated using phase-sensitive HSQC experiments. Mass spectra were obtained with a lockspray dual ion source in combination with a WATERS Alliance 2695 LC system, or with a type Q-TOF premier (MICROMASS) spectrometer (ESI mode) in combination with a WATERS Acquity UPLC system equipped with a WATERS Acquity UPLC BEH C18 1.7 µm (SN 01473711315545) column (solvent A: water + 0.1 % {v/v} formic acid, solvent B: MeCN or MeOH {given in experimental part} + 0.1 % {v/v} formic acid; flow rate = 0.4 mL/min; gradient {t [min]/solvent B [%]}: {0/5} {2.5/95} {6.5/95} {6.6/5} {8/5}; retention times {r_t} given in the experimental part). Ion mass signals (*m/z*) are reported as values in atomic mass units.

GC/MS analyses were carried out either with an HP 6890 chromatograph with Gerstel MPSII and KAS 4 injector, coupled to an HP 5973 quadrupole mass selective detector. Samples were analysed on an Optima 5 column (poly(5%-phenyl-95%-methylsiloxane), 30 m x 0.32 mm i.d. x film thickness 0.25 µm). Carrier gas, He; injector temp., 60°C to 300°C at 12°C/min, splitless; temp. program: 50°C (isothermal 1 min) to 300°C, at 20 °C/min and held isothermal for 6 min at 300°C; ion source: EI, ionisation energy, 70 eV; EI mass spectra were acquired over the mass range of 40 – 500 amu. Further GC/MS analyses were carried out on with an Agilent 7890B GC with 5977B GC/MSD and Gerstel MPS Robotic XL with KAS 4C injector. Samples were analysed on an Optima 5HT column, 30 m x 250 µm i.d. x film thickness 0.25 µm). Carrier gas, He; injector temp., 60°C to 300°C at 12°C/min, splitless; temp. program: 50°C (isothermal 1 min) to 300°C, at 20 °C/min and held isothermal for 6.5 min at 300°C; FID: 300°C, H_2: 30 mL/min, N_2: 25 mL/min, MSD: ion source: EI 70 eV, 230 °C; detector: quadrupole, EI mass spectra were acquired over the mass range of 30 –650 amu.

HR-GC/MS analyses were carried out on a Waters GCT Premier mass spectrometer coupled with an Agilent 6890n GC with CTC CombiPAL sampler. Samples were analysed on an Optima 5HT column, 30 m x 250 µm i.d. x film thickness 0.25 µm). Carrier gas, He; injector temp. 300°C, split ratio 1:40; temp. program: 50°C (isothermal 1 min) to 300°C, at 20 °C/min and held isothermal for 6.5 min at 300°C; FID: 300°C, H_2: 30 mL/min, N_2: 25 mL/min, GCT-Premier: ion source: EI 70 eV, 250 °C; detector-voltage: 2500 V, EI mass spectra were acquired over the mass range of 20 –800 amu. HR-CI-MS was performed with a HP 6890 Series GC-system by Hewlett Packard.

HR-ESI-MS analysis was performed on Waters LCT Premier mass spectrometer coupled with a Waters Alliance 2695 HPLC. The sample was dissolved in a suitable solvent (approx.. 1 mg/mL) and diluted in methanol (1:100). An aliquot of 5 µL was injected in constant flow of methanol without any HPLC column installed and data were recorded in positive or negative ion mode.

Heating of reactions was performed in oil baths. Analytical thin-layer chromatography was performed using precoated silica gel plates (Macherey-Nagel, Düren) and the spots were visualised with UV light at 254 nm or alternatively by staining with permanganate or 4-methoxybenzaldehyde solutions. Commercially available reagents, chromatography type or dry solvents were used as received or purified by standard techniques according to the literature.

Ion exchange chromatography was performed using the Amberchrom® 50WX8 (H^+-form, 100-200 mesh) which can be reused up to ten times. 100 g of the material were taken up in water and washed with an excess of 6% aq. NH_3 (1/4 concentrated). The residue was then washed with an excess of water and transferred into a column where it was stored in an upright position. The column was stored in its H^+-form, therefore the material was washed with an excess of 3 M HCl until pH = 1 was reached. After that, it was washed with an excess of water until pH = 7 was reached. Prior to use, the column was washed with 6% aq. NH_3 (~50 mL) until the material changed its color from brown to light orange. Then the column was washed with an excess of water (~200 mL) until pH = 7 was reached. Then it was equilibrated with IEB (aq. 25 mM NH_4HCO_3 with 2% *i*PrOH, ~100 mL). At this point, the reaction mixture was dissolved in IEB and loaded onto the column which was performed using gravity-powered flow. When the purification was finished the material was transformed back to its H^+-form by washing it with 3 M HCl (~50 mL) until pH = 1 was reached (the color changes from light orange back to brown). Then it was washed with an excess of water (~200 mL) until pH = 7 was reached. The eluent was concentrated *in vacuo* and the residue was lyophilized overnight. The residue was dissolved in 0.05 M NH_4HCO_3 (2 mL) and transferred into a 15 mL Falcon-tube. Then a 1:1 mixture of *i*PrOH/MeCN (9 mL) was added and the mixture was thoroughly mixed using a vortex. The suspension was centrifuged at 5000 g for 10 min and the supernatant was collected. The solid was redissolved in 0.05 M NH_4HCO_3 (2 mL) and a 1:1 mixture of *i*PrOH/MeCN (9 mL) was added and the mixture was thoroughly mixed using a vortex. The suspension was centrifuged at 5000 rpm for 10 min and the supernatant was collected. The comb. supernatants were concentrated *in vacuo* and lyophilized overnight to give the desired pyrophosphates.

1.2 Genes, strains, plasmids and growth conditions

E. coli cultures were grown at 37 °C and 180 rpm in Luria-Bertani medium (LB medium: 0.5 % yeast extract, 1 % tryptone/peptone, 0.05 % sodium chloride) supplemented with 50 µg mL^{-1} kanamycine. For heterologous expression 2TY medium (1 % yeast extract, 1.6 % tryptone/pepton, 0.5 % sodium chloride) supplemented with 50 µg mL^{-1} kanamycin was inoculated by 2% pre-culture of *E. coli* BL21 (DE3) and grown at 37 °C and 180 rpm to an optical density (OD) of 0.6. Then, the expression controlled by the T7lac-promotor was induced by adding 0.5 mM IPTG (isopropyl-*β*-thiogalactopyranoside) and the temperature was decreased to 16 °C for overnight cultivation.

1.3 Protein purification

Terpene cyclase Bot2 was overexpressed as described above. After harvesting, the cells were suspended in lysis buffer (100 mM Tris, 40 mM NaCl, 25 mM imidazole), disrupted by sonification (sonotrode: KE76, I= 37%, 4s/6s) and the histidine-tagged protein was purified by affinity chromatography. Therefore, Protino® Ni-NTA-Agarose (Macherey-Nagel GmbH & Co. KG) was used Protein concentrations were determined by using UV/VIS spectroscopy.

1.4 Large scale heterologous production and purification

Transformed *E. coli* BL21(DE3) containing the pET-28a(+) plasmid, harboring the genetic information of BcBOT2, was used for heterologous production of the sesquiterpene cyclase. The gene was expressed in TB (terrific broth) medium (12 g l^{-1} tryptone, 24 g l^{-1} yeast extract, 5 g l^{-1} glycerol, 100 ml l^{-1} potassium

phosphate buffer). Precultures were carried out in 100 mL LB (lysogeny broth) medium (10 g l^{-1} tryptone, 5 g l^{-1} yeast extract, 10 g l^{-1} NaCl) supplemented with 50 µg mL^{-1} kanamycin in 500 mL baffled shake flasks. Precultures were inoculated with glycerol stocks of cells and incubated overnight at 37 °C and 150 rpm in an orbital shaker. The main cultures were carried out in a 2 l stirred tank bioreactor system (Biostat A+, Sartorius) with a working volume of 1.8 l TB medium supplemented with 50 µg mL^{-1} kanamycin. The bioreactor was equipped with two Rushton impellers. The reactor was gassed with compressed air with an aeration rate of 1 vvm. The stirrer speed is cascaded (400 – 700 rpm) so that the oxygen partial pressure (pO_2) in the culture does not decrease below 30 %. Foam production was controlled by the addition of antifoam. The pH value was set to 7.0 and was controlled by titration with 1 M HCl and 25 % ammonia. The bioreactor was inoculated with preculture to give an initial OD_{600} of 0.1 rel. AU. Fermentation was carried out at 37 °C till induction. Gene expression was induced at an OD_{600} of 1.0 rel. AU by addition of 0.5 mM isopropyl-ß-thiogalactopyranoside (IPTG) and the temperature was lowered to 16 °C to prevent inclusion body formation. The overexpression was carried out for 24 h.

After overexpression of BcBOT2, cells were harvested by centrifugation (5,000 rpm, 30 min, and 4 °C) and suspended in TRIS buffer (40 mM TRIS-HCl, 100 mM NaCl) containing 25 mM imidazole. The cells were disrupted by high-pressure homogenization (15,000 psi, 5 cycles), and cell debris was removed by centrifugation (5,000 rpm, 50 min, and 4 °C). The supernatant was filtered using a bottle top filter with 0.22 µm pore size. The BcBOT2 enzyme was purified by Ni^{2+}-decorated IMAC using the ÄKTAstart™ (Cytiva) system. Ni-NTA Column (HisTrap™ Fast Flow Crude, Cytiva) was loaded with the filtered supernatant. BcBOT2 was eluted with imidazole using a gradient with a maximum concentration of 250 mM. Proteins were detected by absorption at 280 nm and the peak fraction of the elution was collected. Imidazole was removed by buffer exchange using ultrafiltration with a centrifugal concentrator with a cutoff of 30 kDa. BcBOT2 enzyme was preserved in preservation buffer (20 mM TRIS-HCl, 1 mM DTT, 100 mM NaCl, 20 % (v/v) glycerol) and stored at -80 °C.

1.5 Analytical enzyme tests

For a qualitative and semi-quantitative biotransformation the desired pyrophosphate (150 µM) was incubated with 0.1 g/L of the corresponding STC together with an 50 mM HEPES-buffer (pH = 7,5), 5 mM DTT and 5 mM $MgCl_2$ in a final volume of 0.5 mL in a small glass vial. The mixture was incubated at 37 °C and 200 rpm for 30 min and then extracted with 100 µL of GCMS-grade hexanes. The layers were separated using centrifugation at 2000 rpm for 4 min at 4 °C. Then 60 µL of the org. layer was collected and submitted for GC-MS analysis (see attachments below).

2 Chemical syntheses

2.1 Synthesis of tris(tetra-*n*-butylammonium)hydrogenpyrophosphate trihydrate S1

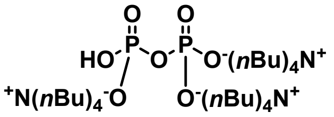

S1

$Na_2P_2O_7H_2$ (4.32 g, 19.45 mmol, 1.00 eq) was dissolved in water (32.5 mL) and conc. NH_3 (aq. 25%, 1.4 mL) was added. The mixture was loaded onto a column of Dowex AG 50W-X8 cation exchange resin (100–200 mesh, H^+) and eluted with 150 mL of water. The eluent was titrated with $(n\text{Bu})_4$OH (40% in water) until a pH of 7.3 is reached. The mixture is concentrated *in vacuo* and the residue was dissolved in water, frozen in liquid nitrogen and lyophilized overnight to yield pyrophosphate **S1** (17.56 g, 19.45 mmol, *quant.*) as a white solid which is stored at 4 °C in the glove box. The analytical data match those reported in the literature.[S1]

^{1}H-NMR (400 MHz, $CDCl_3$): δ = 3.19-3.15 (m, 24H), 1.66-1.58 (m, 24H), 1.33 (se, 7.34 Hz, 24H), 0.92 (t, 7.36 Hz, 36H).

2.2 Synthesis of farnesylpyrophosphate derivative 10

2.2. Synthesis of C-9 oxy farnesyl derivative 20 and 21

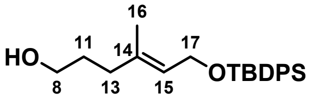

S2

$NaBH_4$ (1.25 g, 33.10 mmol, 2.00 eq) was slowly added to a stirred solution of aldehyde **13** (6.07 g, 16.55 mmol, 1.00 eq) in EtOH (330 mL) at 0 °C and the resulting mixture was stirred at 0 °C for 20 min. The reaction was terminated by addition of a sat. aq. $NaHCO_3$-solution (100 mL), the aq. layer was extracted with EtOAc (3x 300 mL), the combined organic layers were washed with brine (300 mL), dried over $MgSO_4$, filtered and concentrated *in vacuo.* Column chromatography (PE/EtOAc 5:1) yielded alcohol **S2** (3.87 g, 10.49 mmol, 63%) as a yellow oil. The analytical data match those reported in the literature.[S2]

^{1}H-NMR (400 MHz, $CDCl_3$): δ = 7.70-7.68 (m, 4H, TBDPS), 7.42-7.36 (m, 6H, TBDPS), 5.41 (dt, J = 6.25, 1.25 Hz, 1H, H-15), 4.21 (d, J = 5.84 Hz, 2H, H-8), 3.62 (t, J = 6.36 Hz, 2H, H-17), 2.05 (t, J = 7.97 Hz, 2H, H-13), 1.66 (qi, J = 7.03 Hz, 2H, H-11), 1.46 (s, 3H, H-16), 1.26 (t, J = 7.14 Hz, 1H, OH), 1.04 (s, 9H, TBDPS) ppm; R_f (5:1 PE/EtOAc): 0.55.

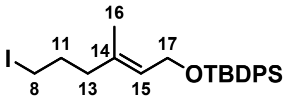

S3

MsCl (0.97 mL, 12.57 mmol, 1.20 eq) and NEt_3 (3.0 mL, 20.94 mmol, 2.00 eq) were added to a stirred solution of alcohol **S2** (3.86 g, 10.47 mmol, 1.00 eq) in CH_2Cl_2 (55 mL) at 0 °C and the resulting mixture was stirred at 0 °C for 2 h. The reaction was terminated by addition of a sat. aq. $NaHCO_3$-solution (20 mL), the aq. layer was extracted with CH_2Cl_2 (3x 20 mL), the comb. org. layers were washed with 1 M HCl (50 mL) and brine (50 mL), dried over $MgSO_4$, filtered and concentrated *in vacuo*. The residue was dissolved in acetone (100 mL) and NaI (2.04 g, 13.61 mmol, 1.30 eq) was added and the resulting mixture was heated to 50 °C overnight. The mixture was concentrated *in vacuo* and the resiue was dissolved in 1:1 mixture of an aq. 10% $Na_2S_2O_3$-solution and a sat. aq. $NaHCO_3$-solution (50 mL), the aq. layer was extracted with Et_2O (3x 30 mL), the combined organic layers were washed with brine (50 mL), dried over $MgSO_4$, filtered and concentrated *in vacuo*. Column chromatography (PE/EtOAc 50:1) yielded iodide **S3** (4.23 g, 8.83 mmol, 84%) as a colorless oil. The analytical data match those reported in the literature.[S2]

^{1}H-NMR (400 MHz, $CDCl_3$): δ = 7.70-7.67 (m, 4H, TBDPS), 7.44-7.36 (m, 6H, TBDPS), 5.42 (dt, J = 6.24, 1.18 Hz, 1H, H-15), 4.22 (d, J = 5.76 Hz, 2H, H-17), 3.13 (t, J = 7.00 Hz, 2H, H-8), 2.07-2.04 (m, 2H, H-13), 1.93-1.86 (m, 2H, H-11), 1.42 (s, 3H, H-16), 1.04 (s, 9H, TBDPS) ppm; R_f (10:1 PE/EtOAc): 0.65.

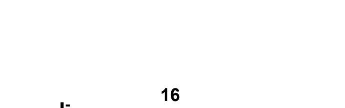

14

A mixture of iodide **S3** (4.22 g, 8.81 mmol, 1.00 eq) and PPh_3 (2.77 g, 10.57 mmol, 1.20 eq) in toluene (45 mL) was heated in a sealed tube under refluxing conditions overnight. The mixture was concentrated *in vacuo* and loaded onto a silica column. Column chromatography (DCM/MeOH 20:1 – 10:1) yielded Wittig salt **14** (6.55 g, 8.81 mmol, *quant.*) as a white solid. The analytical data match those reported in the literature.[S3]

^{1}H-NMR (400 MHz, $CDCl_3$): δ = 7.85-7.78 (m, 9H, PPh_3), 7.71-7.66 (m, 6H, PPh_3), 7.63-7.61 (m, 4H, TBDPS), 7.410-7.31 (m, 6H, TBDPS), 5.32 (t, J = 6.09 Hz, 1H, H-15), 4.15 (d, J = 6.08 Hz, 2H, H-17), 3.78-3.71 (m, 2H, H-8), 2.33 (t, J = 7.10 Hz, 2H, H-13), 1.77 (se, J = 7.86 Hz, 2H, H-11), 1.34 (s, 3H, H-16), 0.98 (s, 9H, TBDPS) ppm; m.p.: 63 °C.

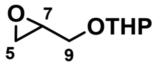
S4

DHP (1.65 mL, 18.22 mmol, 1.35 eq) and *p*TsOH·H_2O (69.7 mg, 0.41 mmol, 0.03 eq) were added to a stirred solution of alcohol **16** (0.90 mL, 13.50 mmol, 1.00 eq) in CH_2Cl_2 (13.5 mL) at 0 °C. The resulting mixture was stirred at 0 °C for 30 min. The reaction was terminated by addition of NEt_3 (0.12 mL, 0.81 mmol, 0.06 eq) and the mixture was concentrated *in vacuo*. Column chromatography (PE/EtOAc 5:1) yielded ether **S4** as a 1:1 mixture of diastereomers (1.70 g, 10.74 mmol, 80%) as a colorless oil. The analytical data match those reported in the literature.[S4]
^{1}H-NMR (400 MHz, $CDCl_3$): δ = 4.76 (t, *J* = 3.92 Hz, 1H, THP), 4.65 (t, *J* = 3.56 Hz, 1H, THP), 3.95 (dd, *J* = 11.58, 3.18 Hz, 1H, H-9), 3.90-2.84 (m, 2H, THP), 3.74 (dd, *J* = 11.70, 5.06 Hz, 1H, H-9), 3.69 (dd, *J* = 11.68, 3.40 Hz, 1H, H-9), 3.53-3.49 (m, 2H, THP), 3.40 (dd, *J* = 11.76, 6.32 Hz, 1H, H-9), 3.22-3.17 (m, 2H, H-7), 2.83-2.80 (m, 2H, H-5), 2.69 (dd, *J* = 5.13, 2.64 Hz, 1H, H-5), 2.60 (4.86, 2.58 Hz, 1H, H-5), 1.85-1.52 (m, 12H, THP) ppm; R_f (5:1 PE/EtOAc): 0.31.

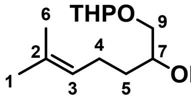
S5

In a two-neck flask equipped with a reflux-condenser Mg turnings (3.12 g, 128.19 mmol, 12.00 eq) and a single crystal of I_2 in THF (8.5 mL) were placed. A solution of prenylchloride **15** (3.6 mL, 32.05 mmol, 3.00 eq) in THF (55.0 mL) was added at 0 °C slowly (0.33 mL/min). The resulting mixture was stirred at 0 °C for 1 h and then transferred to a new flask. The Grignard reagent was titrated three times using menthol and phenantrolin, determining its concentration to be 0.52 M. In a new flask, the prepared Grignard reagent (24.7 mL, 12.82 mmol, 1.20 eq) was added dropwise to a stirred solution of CuI (203.4 mg, 1.07 mmol, 0.10 eq) and epoxide **S4** (1.69 g, 10.68 mmol, 1.00 eq) in THF (50 mL) at -60 °C and the resulting mixture was allowed to warm to -20 °C o/n using a cryostat The reaction was terminated by addition of ice (20 g) and allowed to warm to rt. Then a sat. aq. NH_4Cl-solution (20 mL) was added and the aq. layer was extracted with EtOAc (4x 20 mL), the combined organic layers were washed with a sat. aq. $NaHCO_3$-solution, brine (50 mL), dried over $MgSO_4$, filtered and concentrated *in vacuo* to yield crude alcohol **S5** (2.15 g, 9.40 mmol, 88%) as a yellow oil which was directly used for the next step without further purification.
HRMS (ESI): *m/z calc.* for $C_{13}H_{24}O_3Na$ $[M+Na]^+$: 251.1623; *found*: 251.1626; R_f (2:1 PE/EtOAc): 0.29.

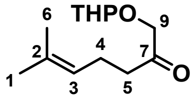
17

DMSO (2.0 mL, 28.12 mmol, 3.00 eq) was added dropwise to a stirred solution of oxalylchloride (1.2 mL, 14.06 mmol, 1.50 eq) in CH_2Cl_2 (35.0 mL) at -78 °C, the resulting mixture was stirred at -78 °C for 10 min, then alcohol **S5** (2.14 g, 9.37 mmol, 1.00 eq) in CH_2Cl_2 (23.5 mL) was added dropwise at -78 °C and the mixture was stirred at -78 °C for 15 min. Then NEt_3 (4.0 mL, 28.12 mmol, 3.00 eq) was added dropwise at -78 °C and the mixture was allowed to warm to rt and stirred at rt for 30 min. The reaction was terminated by addition of a sat. aq. $NaHCO_3$-solution (50 mL), the layers were separated, the aq. layer was extracted with CH_2Cl_2 (3x 50 mL), the combined organic layers were washed with brine (100 mL), dried over $MgSO_4$, filtered and concentrated *in vacuo*. Column chromatography (PE/EtOAc 20:1) yielded ketone **17** (1.43 g, 6.31 mmol, 67%) as a yellow oil. The analytical data match those reported in the literature.[S5]
^{1}H-NMR (400 MHz, $CDCl_3$): δ = 5.08 (t, *J* = 6.48 Hz, 1H, H-3), 4.64 (t, *J* = 3.45 Hz, 1H, THP), 4.25 (d, *J* = 16.51 Hz, 1H, H-9), 4.13 (d, *J* = 17.25 Hz, 1H, H-9), 3.86-3.850 (m, 1H, THP), 3.52-3.50 (m, 1H, THP), 2.50 (dt, *J* = 7.42, 1.83 Hz, 2H, H-5), 2.28 (q, *J* = 7.35 Hz, 2H, H-4), 1.89-1.70 (m, 3H, THP), 1.67 (s, 3H, H-6), 1.62 (s, 3H, H-1), 1.60-1.54 (m, 3H, THP) ppm; R_f (5:1 PE/EtOAc): 0.47.

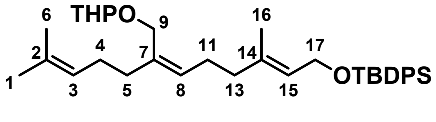

19

*n*BuLi (1.6 M in hex, 1.4 mL, 2.16 mmol, 1.60 eq) was added dropwise to a stirred solution of Wittig salt **14** (1.00 g, 1.35 mmol, 1.00 eq) in a mixture of THF (22.5 mL) and HMPA (1.4 mL) at -78 °C and the resulting mixture was stirred at -78 °C for 1 h. Then ketone **17** (0.37 g, 1.62 mmol, 1.20 eq) in THF (3.3 mL) was added dropwise at -78 °C. The resulting mixture was allowed to warm to rt overnight. The reaction was terminated by addition of a sat. aq. NH_4Cl-solution (20 mL), the aq. layer was extracted with Et_2O (3x 20 mL), the combined organic layers were washed with water (2x 20 mL), brine (30 mL), dried over $MgSO_4$, filtered and concentrated *in vacuo*. Column chromatography (PE/EtOAc 50:1) yielded olefine **19** (0.54 g, 0.96 mmol, 71%) as a colorless oil.

^{1}H-NMR (400 MHz, $CDCl_3$): δ = 7.71-7.67 (m, 4H, TBDPS), 7.44-7.35 (m, 6H, TBDPS), 5.41-5.35 (m, 2H, H-8, H-15), 5.13-5.09 (m, 1H, H-3), 4.59 (t, J = 3.52 Hz, 1H, THP), 4.22 (dd, J = 6.28, 0.51 Hz, 2H, H-17), 4.18 (d, J = 11.48 Hz, 1H, H-9), 4.04 (d, J = 11.42 Hz, 1H, H-9), 3.91-3.86 (m, 1H, THP), 3.54-3.49 (m, 1H, THP), 2.21-1.99 (m, 8H, H-4, H-5, H-11, H-13), 1.88-1.79 (m, 1H, THP), 1.74-1.70 (m, 1H, THP), 1.68 (s, 3H, H-1), 1.63-1.49 (m, 7H, THP, H-16), 1.44 (s, 3H, H-6) ppm; ^{13}C-NMR (100 MHz, $CDCl_3$): δ = 136.8 (q, C-7), 135.9 (q, C-14), 135.7 (t, TBDPS), 134.2 (q, C-2), 131.5 (q, TBDPS), 129.6 (t, TBDPS), 129.1 (t, C-8), 127.7 (t, TBDPS), 124.5 (t, C-15), 124.4 (t, H-3), 97.8 (t, THP), 64.4 (s, C-9), 62.3 (s, THP), 61.3 (s, C-17), 39.9 (s, C-13), 35.6 (s, C-5), 30.8 (s, THP), 27.1 (s, C-11), 27.0 (p, TBDPS), 26.1 (s, C-4), 25.8 (p, C-1), 25.7 (s, THP), 19.7 (s, THP), 19.3 (p, C-16), 17.9 (q, TBDPS), 16.4 (p, C-6) ppm.

HRMS (ESI): *m/z calc.* for $C_{36}H_{52}O_3SiNa$ $[M+Na]^+$: 583.3583; *found*: 583.3581; R_f (10:1 PE/EtOAc): 0.58.

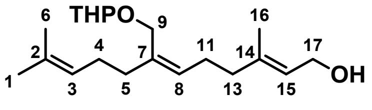

S6

TBAF (1 M in THF, 2.9 mL, 2.86 mmol, 3.00 eq) was added dropwise to a stirred solution of TBDPS-ether **S5** (0.54 g, 0.95 mmol, 1.00 eq) in THF (16.0 mL) at 0 °C and the resulting mixture was allowed to warm to rt overnight. The reaction was terminated by addition of a sat. aq. NH_4Cl-solution (20 mL), the aq. layer was extracted with EtOAc (3x 20 mL), the combined organic layers were washed with brine (30 mL), dried over $MgSO_4$, filtered and concentrated *in vacuo*. Column chromatography (PE/EtOAc 3:1) yielded alcohol **S6** (0.28 g, 0.88 mmol, 93%) as a colorless oil.

^{1}H-NMR (400 MHz, $CDCl_3$): δ = 5.41 (tq, J = 7.01, 1.24 Hz, 1H, H-8), 5.34 (t, J = 7.29 Hz, 1H, H-15), 5.13-5.09 (m, 1H, H-3), 4.59 (t, J = 3.41 Hz, 1H, THP), 4.18 (d, J = 11.44 Hz, 1H, H-9), 4.14 (d, J = 7.00 Hz, 2H, H-17), 4.02 (d, J = 11.43 Hz, 1H, H-9), 3.91-3.85 (m, 1H, THP), 3.55-3.49 (m, 1H, THP), 2.24-2.19 (m, 2H, H-13), 2.15-2.04 (m, 6H, H-4, H-5, H-11), 1.87-1.69 (m, 2H, THP), 1.68 (s, 6H, H-1, H-16), 1.63-1.51 (m, 7H, H-6, THP) ppm; ^{13}C-NMR (100 MHz, $CDCl_3$): δ = 139.3 (q, C-7), 136.1 (q, C-14), 131.6 (q, C-2), 128.9 (t, C-7), 124.4 (t, C-15), 124.0 (t, C-3), 97.9 (t, THP), 64.4 (s, C-9), 62.3 (s, THP), 59.5 (s, C-17), 39.7 (s, C-13), 35.5 (s, C-5), 30.8 (s, THP), 27.1 (s, C-11), 26.1 (s, C-4), 25.8 (p, C-1), 25.7 (s, THP), 19.6 (s, THP), 17.9 (p, C-16), 16.5 (p, C-6) ppm.

HRMS (ESI): *m/z calc.* for $C_{20}H_{34}O_3Na$ $[M+Na]^+$: 345.2406; *found*: 345.2411; R_f (3:1 PE/EtOAc): 0.38.

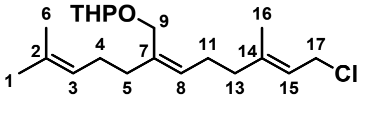

S7

DMS (0.96 mL, 1.30 mmol, 1.50 eq) was added dropwise to a stirred solution of NCS (150.7 mg, 1.13 mmol, 1.30 eq) in CH_2Cl_2 (2.3 mL) at -30 °C. The resulting mixture was allowed to warm to 0 °C over 10 min, then it was cooled to -30 °C. Alcohol **S6** (0.28 g, 0.87 mmol, 1.00 eq) in CH_2Cl_2 (1.5 mL) was added dropwise at

-30 °C and the resulting mixture was allowed to warm to 0 °C over 1 h. The reaction was terminated by addition of brine (10 mL), the aq. layer was extracted with Et_2O (3x 10 mL), the combined organic layers were washed with brine (20 mL), dried over $MgSO_4$, filtered and concentrated *in vacuo*. Column chromatography (PE/EtOAc 10:1) yielded chloride **S7** (0.26 g, 0.76 mmol, 87%) as a colorless oil.
^{1}H-NMR (400 MHz, $CDCl_3$): δ = 5.44 (tq, J = 11.99, 1.30 Hz, 1H, H-8), 5.34 (t, J = 7.16 Hz, 1H, H-15), 5.13-5.09 (m, 1H, H-3), 4.59 (t, J = 3.41 Hz, 1H, THP), 4.17 (d, J = 11.33 Hz, 1H, H-9), 4.09 (d, J = 7.92 Hz, 2H, H-17), 4.02 (d, J = 11.55 Hz, 1H, H-9), 3.91-3.85 (m, 1H, THP), 3.55-3.49 (m, 1H, THP), 2.25-2.19 (m, 2H, H-13), 2.14-2.06 (m, 5H, H-4, H-5, H-11), 1.87-1.77 (m, 1H, THP), 1.74-1.66 (m, 7H, THP, H-1, H-16), 1.63-1.50 (m, 7H, THP, H-6) ppm; ^{13}C-NMR (100 MHz, $CDCl_3$): δ = 142.5 (q, C-7), 136.3 (q, C-14), 131.6 (q, C-2), 128.6 (t, C-8), 124.4 (t, C-15), 120.7 (t, C-3), 97.8 (t, THP), 64.4 (s, C-9), 62.3 (s, THP), 41.2 (s, C-17), 39.8 (s, C-13), 35.5 (s, C-5), 30.8 (s, THP), 27.1 (s, C-11), 25.9 (s, C-4), 25.9 (p, C-1), 25.7 (s, THP), 19.6 (s, THP), 17.9 (p, C-16), 16.2 (p, C-6) ppm.
HRMS (ESI): *m/z calc.* for $C_{20}H_{33}O_2ClNa$ $[M+Na]^+$: 363.2067; *found*: 363.2071; R_f (2:1 PE/EtOAc): 0.69.

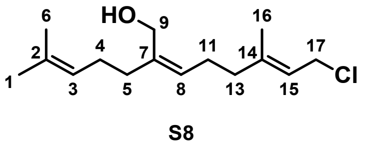

S8

pTsOH·H_2O (7.7 mg, 0.05 mmol, 0.10 eq) was added to a stirred solution of THP-acetal **S7** (152.0 mg, 0.45 mmol, 1.00 eq) in wet MeOH (5.6 mL) at 0 °C. The resulting mixture was allowed to warm to rt over 1 h. The reaction was terminated by addition of a sat. aq. $NaHCO_3$-solution (20 mL). The aq. layer was extracted with CH_2Cl_2 (3x 20 mL), the combined organic layers were washed with brine (50 mL), dried over $MgSO_4$, filtered and concentrated *in vacuo*. Column chromatography yielded alcohol **S8** (54.5 mg, 0.21 mmol, 48%) as a colorless oil.
^{1}H-NMR (400 MHz, $CDCl_3$): δ = 5.45 (dt, J = 7.83, 0.78 Hz, 1H, H-8), 5.28 (t, J = 7.27 Hz, 1H, H-15), 5.13-5.09 (m, 1H, H-3), 4.12 (s, 2H, H-9), 4.09 (d, J = 8.02 Hz, 2H, H-17), 2.25-2.20 (m, 2H, H-13), 2.15-2.07 (m, 6H, H-4, H-5, H-11), 1.73 (s, 3H, H-1), 1.69 (s, 3H, H-16), 1.61 (s, 3H, H-6) ppm; ^{13}C-NMR (100 MHz, $CDCl_3$): δ = 142.3 (q, C-7), 139.0 (q, C-14), 132.0 (q, C-2), 127.9 (t, C-8), 124.2 (t, C-15), 121.0 (t, C-3), 60.5 (s, C-9), 41.2 (s, C-17), 39.7 (s, C-13), 35.3 (s, C-5), 27.2 (s, C-11), 25.8 (s, C-4, C-1), 17.9 (p, C-16), 16.3 (p, C-6) ppm.
HRMS (ESI): *m/z calc.* for $C_{15}H_{25}OClNa$ $[M+Na]^+$: 279.1492; *found*: 279.1492; R_f (3:1 PE/EtOAc): 0.38.

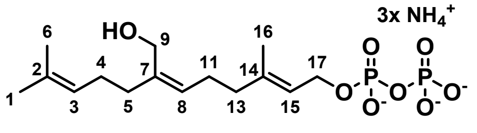

10

Preactivated molecular sieves (3Å) were added to a stirred solution of $(nBu_4N)_3P_2O_7H$ (351.4 mg, 0.39 mmol, 2.00 eq) in acetonitrile (3.9 mL) at 0 °C. Then chloride **S8** (50.0 mg, 0.19 mmol, 1.00 eq) in acetonitrile (2.0 mL) was added dropwise at 0 °C and the resulting mixture was allowed to warm to rt and stirred at rt for 12 h. The mixture was concentrated *in vacuo* and standard work up using an ion exchange column was applied to yield pyrophosphate **10** (80.6 mg, 0.18 mmol, 92%) as a white gum which was stored under an argon atmosphere at -80 °C.
^{1}H-NMR (600 MHz, D_2O): δ = 5.47 (dt, J = 7.13, 1.03 Hz, 1H, H-8), 5.42 (t, J = 7.36 Hz, 1H, H-15), 5.22-5.19 (m, 1H, H-3), 4.49 (t, J = 6.35 Hz, 2H, H-17), 4.13 (s, 2H, H-9), 2.27-2.23 (m, 2H, H-13), 2.16-2.11 (m, 6H, H-4, H-5, H-11), 1.73 (s, 3H, H-1), 1.70 (s, 3H, H-16), 1.62 (s, 3H, H-6) ppm; ^{13}C-NMR (150 MHz, D_2O): δ = 142.7 (q, C-7), 137.4 (q, C-14), 133.6 (q, C-2), 129.0 (t, C-8), 124.3 (t, C-3), 119.6 (d, J = 8.01 Hz, t, C-15), 62.9 (d, J = 5.59 Hz, s, C-17), 58.6 (s, C-9), 39.0 (s, C-13), 34.1 (s, C-5), 26.0 (s, C-11), 25.2 (s, C-4), 24.8 (p, C-1), 17.0 (p, C-16), 15.6 (p, C-6) ppm; ^{31}P-NMR (162 MHz, D_2O): δ = -9.93 - -10.69 (m, 2P) ppm.

HRMS (ESI): m/z calc. for $C_{15}H_{27}O_8P_2$ $[M-H]^-$: 397.1181; found: 397.1176.

2.3 Synthesis of farnesylpyrophosphate derivative 11

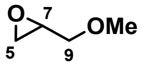

S9

A mixture of *rac*-glycidol (**16**) (1.8 mL, 27.00 mmol, 1.00 eq), Ag_2O (6.26 g, 27.00 mmol, 1.00 eq), MeI (16.8 mL), 269.98 mmol, 10.00 eq) and 3Å-sieves in CH_2Cl_2 (16.0 mL) were heated under refluxing conditions overnight. The mixture was cooled to rt and filtered through a pad of Celite™ and washed with CH_2Cl_2 (30 mL). CH_2Cl_2 and remaining MeI were distilled off at 80 °C (1 atm) yielding epoxide **S9** (2.38 g, 26.99 mmol, *quant.*) as a colorless liquid. The analytical data match those reported in the literature.[S6]

^{1}H-NMR (400 MHz, $CDCl_3$): δ = 3.70 (dd, J = 11.39, 2.94 Hz, 1H, H-9), 3.41 (s, 3H, OMe), 3.33 (dd, J = 11.38, 5.87 Hz, 1H, H-9), 3.17-3.13 (m, 1H, H-7), 2.80 (dd, J = 4.93, 4.25 Hz, 1H, H-5), 2.62 (dd, J = 4.99, 2.70 Hz, 1H, H-5) ppm; R_f (5:1 PE/EtOAc): 0.31.

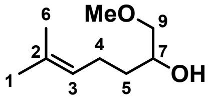

S10

In a two-neck flask equipped with a reflux-condenser Mg turnings (3.59 g, 147.54 mmol, 10.00 eq) and a single crystal of I_2 in THF (10.0 mL) were placed. A solution of prenylchloride (4.2 mL, 69.89 mmol, 2.50 eq) in THF (62 mL) was added at 0 °C slowly (0.33 mL/min). The resulting mixture was stirred at 0 °C for 1 h and then transferred to a new flask. The Grignard reagent was titrated three times using menthol and phenantrolin, determining its concentration to be 0.56 M. In a new flask, the prepared Grignard reagent (31.6 mL, 17.71 mmol, 1.20 eq) was added dropwise to a stirred solution of CuI (281.0 mg, 1.48 mmol, 0.10 eq) and epoxide **S9** (1.30 g, 14.75 mmol, 1.00 eq) in THF (67 mL) at -60 °C and the resulting mixture was allowed to warm to -20 °C overnight using a cryo reactor. The reaction was terminated by addition of ice (20 g) and allowed to warm to rt. Then a sat. aq. NH_4Cl-solution (20 mL) was added and the aq. layer was extracted with EtOAc (4x 20 mL), the combined organic layers were washed with a sat. aq. $NaHCO_3$-solution, brine (50 mL), dried over $MgSO_4$, filtered and concentrated *in vacuo* to yield crude alcohol **S10** (1.70 g, 10.77 mmol, 73%) as a yellow oil which was directly used for the next step without further purification.

HRMS (GC-MS): *m/z calc.* for $C_9H_{18}O_2$ [M]: 158.1307; *found*: 158.1308; R_f (3:1 PE/EtOAc): 0.33.

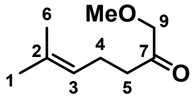

18

DMSO (1.3 mL, 17.96 mmol, 3.00 eq) was added dropwise to a stirred solution of oxalylchloride (0.77 mL, 8.98 mmol, 1.50 eq) in CH_2Cl_2 (22.5 mL) at -78 °C, the resulting mixture was stirred at -78 °C for 10 min, then alcohol **S10** (0.95 g, 5.99 mmol, 1.00 eq) in CH_2Cl_2 (15.0 mL) was added dropwise at -78 °C and the mixture was stirred at -78 °C for 15 min. Then NEt_3 (2.5 mL, 17.96 mmol, 3.00 eq) was added dropwise at -78 °C and the mixture was allowed to warm to rt and stirred at rt for 30 min. The reaction was terminated by addition of a sat. aq. $NaHCO_3$-solution (50 mL), the layers were separated, the aq. layer was extracted with CH_2Cl_2 (3x 30 mL), the combined organic layers were washed with brine (80 mL), dried over $MgSO_4$, filtered and concentrated *in vacuo* at 200 mbar and 35 °C. Column chromatography (pentanes/Et_2O 1:1) yielded ketone **18** (0.81 g, 5.19 mmol, 87%) as a yellow oil.

^{1}H-NMR (400 MHz, $CDCl_3$): δ = 5.06 (t, J = 7.19 Hz, 1H, H-3), 4.00 (s, 2H, H-9), 3.41 (s, 3H, OMe), 2.45 (t, J = 7.38 Hz, 2H, H-5), 2.27 (q, J = 7.40 Hz, 2H, H-4), 1.67 (s, 3H, H-1), 1.61 (s, 3H, H-6) ppm; ^{13}C-NMR (100 MHz, $CDCl_3$): δ = 208.6 (q, C-7), 133.2 (q, C-2), 122.6 (t, C-3), 78.8 (s, C-9), 59.4 (p, OMe), 39.0 (s, C-5), 25.8 (p, C-1), 22.2 (s, C-4), 17.8 (p, C-6) ppm.

HRMS (GC-MS): *m/z calc.* for $C_9H_{16}O_2$ [M]: 156.1150; *found*: 156.1150; R_f (3:1 PE/EtOAc): 0.43.

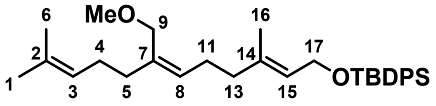

20

nBuLi (1.6 M in hexane 0.87 mL, 1.38 mmol, 1.60 eq) was added dropwise to a stirred solution of Wittig salt **14** (0.64 g, 0.86 mmol, 1.00 eq) in a mixture of THF (14.5 mL) and HMPA (0.87 mL) at -78 °C and the resulting mixture was stirred at -78 °C for 1 h. Then ketone **18** (162.0 mg, 1.04 mmol, 1.20 eq) in THF (2.1 mL) was added dropwise at -78 °C. The resulting mixture was allowed to warm up to rt overnight. The reaction was terminated by addition of a sat. aq. NH_4Cl-solution (10 mL), the aq. layer was extracted with Et_2O (3x, 10 mL), the combined organic layers were washed with water (2x 20 mL), brine (30 mL), dried over $MgSO_4$, filtered and concentrated *in vacuo*. Column chromatography (PE/EtOAc 50:1 – 20:1) yielded triene **20** (174.8 mg, 0.36 mmol, 40%) as a colorless oil.

^{1}H-NMR (400 MHz, $CDCl_3$): δ = 7.71-7.68 (m, 4H, TBDPS), 7.44-7.35 (m, 6H, TBDPS), 5.40-5.35 (m, 2H, H-8, H-15), 5.12-5.08 (m, 1H, H-3), 4.22 (dd, J = 6.29, 0.53 Hz, 2H, H-17), 3.92 (s, 2H, H-9), 3.30 (s, 3H, OMe), 2.19-1.98 (m, 8H, H-4, H-5, H-11, H-13), 1.68 (s, 3H, H-6), 1.60 (s, 3H, H-16), 1.44 (s, 3H, H-1), 1.04 (s, 9H, TBDPS) ppm; ^{13}C-NMR (100 MHz, $CDCl_3$): δ = 136.8 (q, C-7), 136.0 (q, C-14), 125.8 (t, TBDPS), 134.2 (q, TBDPS), 131.6 (q, C-2), 129.6 (t, TBDPS), 129.1 (t, C-8), 127.7 (t, TBDPS), 124.5 (t, C-15), 124.4 (t, C-2), 69.8 (s, C-9), 61.3 (s, C-17), 57.9 (p, OMe), 39.8 (s, C-13), 35.4 (s, C-5), 27.0 (p, TBDPS, C-13), 26.2 (s, C-11), 25.8 (p, C-6), 19.3 (q, TBDPS), 17.9 (p, C-16), 16.5 (p, C-1) ppm.

HRMS (ESI): *m/z calc.* for $C_{32}H_{46}O_2SiNa$ $[M+Na]^+$: 513.3165; *found*: 513.3162; R_f (10:1 PE/EtOAc): 0.55.

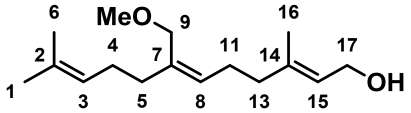

39

TBAF (1 M in THF, 0.86 mL, 0.86 mmol, 3.00 eq) was added dropwise to a stirred solution of TBDPS-ether **S11** (140.0 mg, 0.29 mmol, 1.00 eq) in THF (4.8 mL) at 0 °C and the resulting mixture was allowed to warm to rt overnight. The reaction was terminated by addition of a sat. aq. NH_4Cl-solution (10 mL), the aq. layer was extracted with EtOAc (3x 10 mL), the combined organic layers were washed with brine (20 mL), dried over $MgSO_4$, filtered and concentrated *in vacuo*. Column chromatography (PE/EtOAc 3:1) yielded alcohol **39** (68.6 mg, 0.27 mmol, 95%) as a colorless oil.

^{1}H-NMR (400 MHz, $CDCl_3$): δ = 5.40 (dt, J = 6.89, 0.87 Hz, 1H, H-8), 5.34 (t, J = 7.26 Hz, 1H, H-15), 5.12-5.08 (m, 1H, H-2), 4.13 (d, J = 7.19 Hz, 2H, H-15), 3.89 (s, 2H, H-9), 3.30 (s, 3H, OMe), 2.23-2.17 (m, 2H, H-13), 2.09-2.03 (m, 6H, H-4, H-5, H-11), 1.68 (s, 6H, H-6, H-16), 1.60 (s, 3H, H-1), 1.38 (bs, 1H, OH) ppm; ^{13}C-NMR (100 MHz, $CDCl_3$): δ = 139.2 (q, C-7), 136.4 (q, C-14), 131.6 (q, C-2), 128.7 (t, C-8), 124.4 (t, C-15), 124.2 (t, C-3), 69.8 (s, C-9), 59.4 (s, C-17), 58.1 (p, OMe), 39.5 (s, C-13), 35.4 (s, C-5), 26.9 (s, C-4), 26.1 (s, C-11), 25.8 (p, C-1), 17.9 (p, C-6), 16.4 (p, C-16) ppm; HRMS (ESI-LCT): *m/z calc.* for $C_{16}H_{28}O_2Na$ $[M+Na]^+$: 275.1987; *found*: 275.1976; R_f (2:1 PE/EtOAc): 0.32.

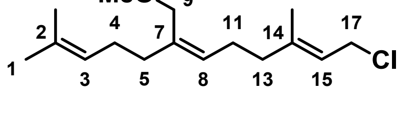

S12

MsCl (0.04 mL, 0.50 mmol, 2.00 eq) was added dropwise to a stirred solution of alcohol **39** (63.4 mg, 0.25 mmol, 1.00 eq) and collidine (0.21 mL, 1.51 mmol, 6.00 eq) in DMF (8.4 mL) at 0 °C and the resulting mixture was stirred at 0 °C for 20 min. Then LiCl (42.6 mg, 1.00 mmol, 4.00 eq) was added at 0 °C and the mixture was allowed to warm to rt over 2 h. The reaction was terminated by addition of brine (10 mL) and diluted with a 1:1 mixture of Et_2O and water (10 mL). The aqueous layer was extracted with Et_2O (3x 10 mL), the comb. org. layers were washed with an aq. 10% $CuSO_4$-solution (20 mL), sat. aq. $NaHCO_3$-solution

(20 mL), brine (20 mL), dried over $MgSO_4$, filtered and concentrated *in vacuo*. Column chromatography (PE/EtOAc 10:1) yielded chloride **S12** (51.8 mg, 0.19 mmol, 76%) as a colorless oil.
^{1}H-NMR (400 MHz, $CDCl_3$): δ = 5.45 (dt, J = 7.96, 1.28 Hz, 1H, H-8), 5.34 (t, J = 7.20 Hz, 1H, H-15), 5.13-5.08 (m, 1H, H-3), 4.09 (d, J = 7.92 Hz, 2H, H-17), 3.91 (s, 2H, H-9), 3.29 (s, 3H, OMe), 2.24-2.18 (m, 2H, H-13), 2.10-2.06 (m, 6H, H-4, H-5, H-11), 1.73 (d, J = 0.65 Hz, 3H, H-6), 1.68 (s, H-16), 1.60 (s, 3H, H-1) ppm; ^{13}C-NMR (100 MHz, $CDCl_3$): δ = 142.4 (q, C-7), 136.5 (q, C-14), 131.6 (q, C-2), 128.5 (t, C-8), 124.4 (t, C-15), 120.8 (t, C-3), 69.8 (s, C-9), 58.0 (p, OMe), 41.2 (s, C-17), 39.7 (s, C-13), 35.4 (s, C-5), 26.9 (s, C-4), 25.9 (s, C-11), 25.8 (p, C-1), 17.9 (p, C-6), 16.3 (p, C-16) ppm.
HRMS (ESI): *m/z calc.* for $C_{16}H_{27}OClNa$ $[M+Na]^+$: 293.1648; *found*: 293.1639; R_f (2:1 PE/EtOAc): 0.66.

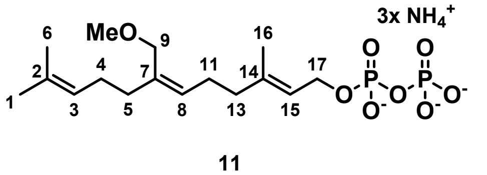

11

Preactivated pieces of 3Å-sieves were added to a stirred solution of $(nBu_4N)_3P_2O_7H$ (333.2 mg, 0.37 mmol, 2.00 eq) in acetonitrile (3.7 mL) at 0 °C. Then chloride **S12** (50.0 mg, 0.18 mmol, 1.00 eq) in acetonitrile (1.9 mL) was added dropwise at 0 °C and the resulting mixture was allowed to warm to rt and stirred at rt overnight. The mixture was concentrated *in vacuo* and standard work up using an ion exchange column was applied to yield pyrophosphate **11** (65.7 mg, 0.14 mmol, 77%) as a white gum which was stored under an atmosphere of Argon at -80 °C.
^{1}H-NMR (600 MHz, D_2O): δ = 5.52 (t, J = 7.36 Hz, 1H, H-8), 5.47 (dt, J = 7.10, 0.96 Hz, 1H, H-15), 5.20 (tt, J = 6.81, 1.34 Hz, 1H, H-3), 4.50-4.48 (m, 2H, H-17), 4.03 (s, 2H, H-9), 3.33 (s, 3H, OMe), 2.28-2.24 (s, 2H, H-13), 2.16-2.08 (s, 6H, H-4, H-5, H-11), 1.73 (s, 3H, H-6), 1.70 (s, 3H, H-16), 1.63 (s, 3H, H-1) ppm; ^{13}C-NMR (150 MHz, D_2O): δ = 142.7 (q, C-7), 134.8 (q, C-14), 133.6 (q, C-2), 130.9 (t, C-8). 124.2 (t, C-15), 119.7 (d, J = 7.88 Hz, t, C-15), 69.2 (s, C-9), 62.9 (d, J = 5.26 Hz, s, C-17), 57.0 (p, OMe), 38.9 (s, C-13), 34.5 (s, C-5), 26.0 (s, C-4), 25.5 (s, C-11), 24.8 (p, C-1), 17.0 (p, C-6), 15.6 (p, C-16) ppm; ^{31}P-NMR (162 MHz, D_2O): δ = -9.98 - -10.48 (m, 2P) ppm.
HRMS (ESI): *m/z calc.* for $C_{16}H_{29}O_8P_2$ $[M-H]^-$: 411.1338; *found*: 411.1335.

3. Biotransformations with unnatural farnesyl pyrophosphates on semi-preparative scale

3.1 Structure elucidations of new biotransformation product 23

Product 23:

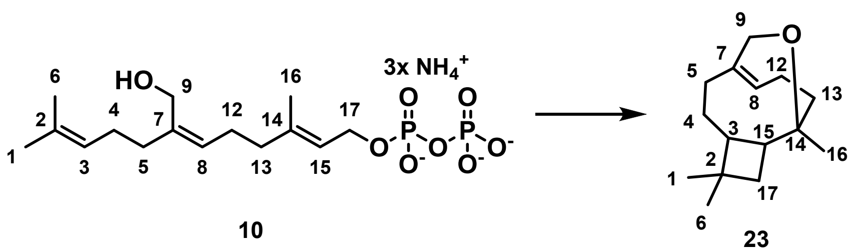

10 **23**

For biotransformations on a large scale to isolate novel products, the desired pyrophosphate (1 mM, 11.2 mg, 0.03 mmol) was incubated with 0.1 g/L of the corresponding STC together in a 50 mM HEPES-buffer (pH = 7.5), 5 mM DTT, 50 mM NaCl, 10 mM $MgCl_2$, 1 U pyrophosphatase and 0.2% (v/v) Tween20 in a final volume of 25 or 50 mL in an Erlenmeyer flask. The mixture was incubated at 37 °C and 100 rpm for 12-24 h and then the same amount of STC was added again to the reaction mixture. Then, incubation was continued and after further 12 h, the mixture was cooled to 15 °C and overlayed with 25 mL of GCMS-ultra grade pentanes and shaken for further 12 h. Then the mixture was transferred into a separation funnel and the layers were separated. The aq. layer was extracted with GCMS-ultra grade pentanes (3x 25 mL) and the comb. org. layers were washed with

brine until no foam forming interphase was present. The org. extracts were dried over $MgSO_4$, filtered and concentrated *in vacuo* (30 °C, 850 mbar). Remaining traces of solvent were evaporated with a stream of argon to yield the crude products which were purified using standard column chromatography (pentanes/Et_2O 1:0 – 1:1) to yield ether **23** (2.7 mg) as a colorless liquid. The fraction still contained traces of pentane..

^{1}H-NMR (500 MHz, C_6D_6): δ = 5.48 (dt, J = 8.03 Hz, 1H, H-8), 4.58 (dd, J = 14.58, 1.08 Hz, 1H, H-9), 3.92 (d, J = 14.60 Hz, 1H, H-9), 3.06-2.98 (m, 1H, H-12), 2.02 (dt, J = 10.12, 8.95 Hz, 1H, H-15), 1.97-1.89 (m, 2H, H-13, H-5), 1.74-1.69 (m, 1H, H-12), 1.68-1.61 (m, 2H, H-3, H-5), 1.49-1.45 (m, 1H, H-17), 1.38-1.32 (m, 3H, H-4, H-13), 1.17-1.15 (m, 1H, H-17), 1.06 (s, 3H, H-16), 0.95 (s, 3H, H-1 or H-6), 0.91 (s, 3H, H-1 or H-6) ppm; ^{13}C-NMR (125 MHz, C_6D_6): δ = 136.3 (q, C-7), 129.0 (t, C-8), 77.7 (q, C-14), 66.4 (s, C-9), 50.3 (t, C-15), 47.8 (t, C-3), 36.6 (s, C-17), 33.8 (s, C-5), 33.7 (s, C-13), 33.3 (q, C-2), 31.3 (p, C-16), 30.1 (p, C-1 or C-6), 36.3 (s, C-4), 23.7 (s, C-12), 22.1 (p, C-1 or C-6) ppm.
HRMS (CI-GC): *m/z calc.* for $C_{15}H_{25}O$ $[M+H]^+$: 221.1905; *found*: 221.1895; R_f (5:1 PE/EtOAc): 0.60.

3.2 Structure elucidations of new biotransformation products 24 – 26

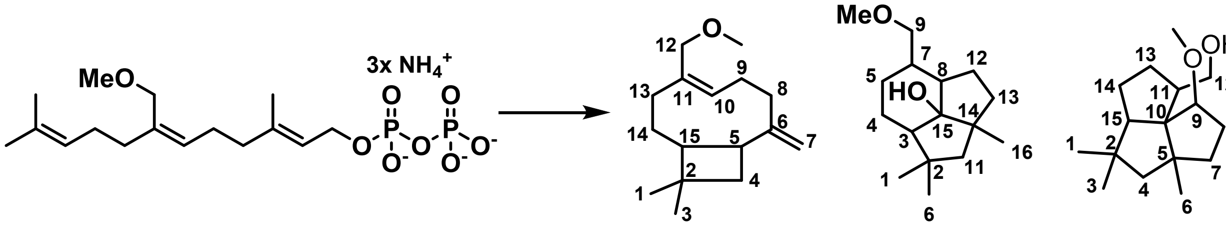

11 **24** **25** **26**

Product isolation was performed in a 1.25 L (15x50 mL, 5x100 mL) scale. FPP derivative **11** (886 mg, 1.91 mmol) was dissolved in an aq. NH_4HCO_3 solution (0.05 M, 102 mL). The following procedure will be described for a 50 mL scale.

Hepes buffer (44.5 mL), FPP derivative **11** solution (500 µL), Tween20® (10 µL), Ppase (1 µL, 100 U/mL) and BcBot2 solution (4.01 mg/mL, 2.5 mg) were mixed. To start the reaction, $MgCl_2$ (2 M, 250 µL) was added and the reaction was incubated at 37 °C and 100 rpm. Every 30 min more FPP derivative **11** solution (500 µL, total volume of 4.00 mL) was added. After 2 h BcBot2 solution (4.01 mg/mL, 2.5 mg, total volume of 1.247 mL) was added. After completing the adding, the reaction stirred at 37 °C and 100 rpm o/n. *n*-pentane was added and the phases were separated. The aqueous phase was extracted with *n*-pentane (3x) and the combined organic phases were washed with brine, dried over $MgSO_4$, filtered and the solvent was carefully removed *in vacuo*. The crude product was purified by column chromatography (*n*-pentane:Et_2O, 5:1 → 3:1 → 1:1). The products **25** (44 mg) and **26** (50 mg) were obtained as colorless crystals. After repeated column chromatography (*n*-pentane:Et_2O, 50:1) of the less polar compounds product **24** was obtained with after co-evaporation with C_6D_6.

Product 24:

^{1}H-NMR (600 MHz, C_6D_6): δ = 5.47 (dd, J = 10.4 Hz, 6.2 Hz, 1H, H-10), 4.97 + 4.79 (s, 2H, H-7), 3.91 (m, 2H, H-12), 3.17 (s, 3H, H-OMe), 2.69 – 2.64 (m, 1H, H-13), 2.36 – 2.30 (m, 1H, H-5), 2.32 – 2.26 (m, 1H, H-9), 2.09 – 2.03 (m, 1H, H-8), 1.96 – 1.93 (m, 1H, H-9), 1.87 – 1.81 (m, 1H, H-8), 1.79 – 1.73 (m, 1H, H-13), 1.70 – 1.62 (m, 3H, H-15, H-4), 1.45 – 1.40 (m, 2H, H-14), 0.98 (s, 3H, H-1/H-3), 0.92 (s, 3H, H-1/H-3) ppm; ^{13}C-NMR (151 MHz, C_6D_6): δ = 153.8 (q, C-6), 136.6 (q, C-11), 129.2 (t, C-10), 113.0 (s, C-7), 69.3 (s, C-12), 57.4 (p, OMe), 52.5 (t, C-15), 49.4 (t, C-5), 40.6 (s, C-4), 35.4 (s, C-13), 35.1 (s, C-8), 33.6 (q, C-2), 30.2 (p, C-1 or C-3) 29.5 (s, C-14), 29.2 (s, C-9), 22.5 (p, C-1 or C-3) ppm.
HRMS (CI-GC): *m/z calc.* for $C_{16}H_{26}O$ $[M-H_2O]^+$: 234.1984; *found*: 234.1988.
R_f (8:1 PE/EtOAc): 0.65.

Product 25:

^{1}H-NMR (600 MHz, C_6D_6): δ = 3.17-3.15 (m, 4H, H-9, OMe), 2.96 (dd, J = 8.83, 7.26 Hz, 1H, H-9), 2.31 (dt, J = 13.56, 8.85 Hz, 1H, H-12), 2.10 (d, J = 11.36 Hz, 1H, H-17), 2.06 (q, J = 9.62 Hz, 1H, H-13), 1.94-1.88 (m, 2H, H-5, H-12), 1.62-1.57 (m, 1H, H-7), 1.51-1.44 (m, 1H, H-4), 1.34 (s, 3H, H-1 or H-6), 1.30-1.26 (m, 1H, H-H-4), 1.26-1.19 (m, 1H, H-8), 1.16 (d, J = 11.03 Hz, 1H, H-17), 1.13 (s, 3H, H-1 or H-6), 1.10 (s, 3H, H-16), 1.00-0.93 (m, 1H, H-5) ppm; ^{13}C-NMR (150 MHz, C_6D_6): δ = 96.2 (q, C-15), 78.0 (s, C-9), 58.8 (p, OMe), 56.6 (q, C-14), 52.5 (t, C-3), 49.2 (s, C-17), 48.0 (q, C-2), 44.5 (t, C-8), 43.3 (t, C-7), 36.5 (p, C-1 or C-6), 34.1 (s, C-13), 33.8 (s, C-12), 29.6 (s, C-5), 28.0 (p, C-16 or C-1 or C-6), 28.0 (p, C-16 or C-1 or C-6), 26.6 (s, C-4) ppm.
HRMS (CI-GC): *m/z calc.* for $C_{16}H_{26}O$ $[M-H_2O]^+$: 234.1984; *found*: 234.1987.
R_f (5:1 PE/EtOAc): 0.33; T_{mp} = 75 – 76 °C.

Product 26:

^{1}H-NMR (500 MHz, C_6D_6): δ = 3.84 (d, J = 5.55 Hz, 2H, H-12), 3.18-3.16 (m, 1H, H-9), 2.83 (s, 3H, OMe), 2.73 (bs, 1H, OH), 2.26-2.21 (m, 1H, H-11), 1.98-1.91 (m, 1H, H-13), 1.72-1.40 (m, 13H, H-15, H-14, H-13, H-4, H-7, H-8),[1] 1.30 (s, 3H, H-6), 0.88 (s, 3H, H-1 or H-3), 0.87 (s, 3H, H-1 or H-3) ppm; ^{13}C-NMR (125 MHz, C_6D_6): δ = 90.4 (t, C9), 71.2 (q, C-10), 64.9 (t, C-15), 64.8 (s, C-12), 59.9 (s, C-4), 55.7 (p, OMe), 49.5 (q, C-5), 47.7 (t, C11), 40.5 (s, C-7 or C-8), 40.1 (q, C-2), 32.2 (p, C-1 or C-3), 32.0 (s, C-13), 27.8 (p, C-6), 26.9 (p, C-1 or C-3), 26.5 (s, C-7 or C-8), 23.7 (s, C-14) ppm.
HRMS (CI-GC): *m/z calc.* for $C_{16}H_{27}O$ $[M-H_2O+H^+]^+$: 235.2062; *found*: 235.2064.
R_f (5:1 PE/EtOAc): 0.12; T_{mp} = 69 – 70 °C.

3.3 Structure elucidations of new biotransformation products 40 – 41

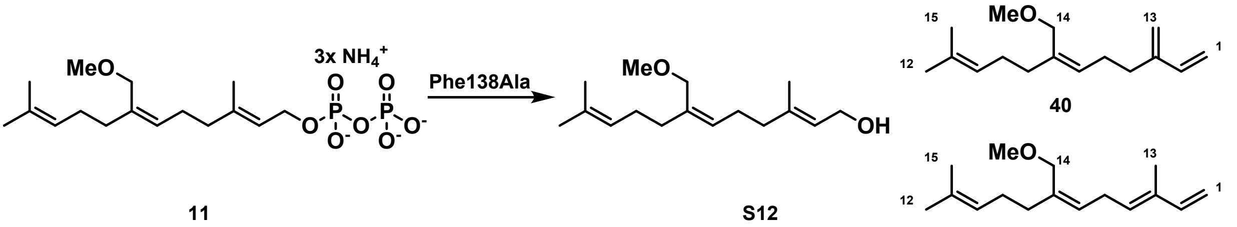

11 S12 40 41

Product isolation was performed in a 550 mL (11x50 mL) scale. FPP derivative **11** (436 mg, 0.94 mmol) were dissolved in an aq. NH_4HCO_3 solution (0.05 M, 50.2 mL). The following procedure will be described for a 50 mL scale.

Hepes buffer (44.5 mL), FPP derivative **11** solution (500 µL), Tween20® (10 µL), Ppase (1 µL, 100 U/mL) and BcBot2 solution (4.16 mg/mL, 2.5 mg) were mixed. To start the reaction, $MgCl_2$ (2 M, 250 µL) was added and the reaction was incubated at 37 °C and 100 rpm. Every 30 min more FPP derivative **11** solution (500 µL, total volume of 4.00 mL) was added. After 2 h BcBot2 solution (4.16 mg/mL, 2.5 mg, total volume of 1.202 mL) was added. After completing the adding, the reaction stirred at 37 °C and 100 rpm o/n. *n*-pentane was added and the phases were separated. The aqueous phase was extracted with *n*-pentane (3x) and the combined organic phases were dried over $MgSO_4$, filtered and the solvent was carefully removed *in vacuo*. The crude product was purified by column chromatography (*n*-pentane:Et_2O, 20:1 → *n*-pentane:EtOAc, 3:1). The mixture **40** and **41** (2 mg) were obtained as a pale yellow oil and **39** (18 mg) as colorless crystals to our surprise. **39** was analysed by GC during the biotransformation and comparison of ^{1}H NMR in C_6D_6 compared to the ^{1}H spectrum obtained during the chemical synthesis in $CDCl_3$. Characteristics for **40** are two olefinic CH_2 groups and

[1] The integral is higher than the 10 protons which appear in this area due to impurities of pentanes.

the missing third methyl group, whereas the CH_2 group in a double allylic position shows the common chemical shift of about 2.85 ppm in structure **41**.

Product 40

^{1}H-NMR (600 MHz, C_6D_6): δ = 6.35 (dd, *J* = 17.6 Hz, 10.1 Hz, 1H, H-2), 5.38 (t, *J* = 6.9 Hz, 1H, H-6), 5.29 – 5.25 (m, 1H, H-10), 5.19 (d, *J* = 17.6 Hz, 1H, H-1), 4.98 – 4.96 (m, 3H, H-1, H-13), 3.88 (s, 2H, H-14), 3.11 (s, 3H, H-OMe), 2.29 – 2.20 (m, 8H, H-4, H-5, H-8, H-9), 1.68 (s, 3H, H-12), 1.58 (s, 3H, H-15) ppm; ^{13}C-NMR (151 MHz, C_6D_6): δ = 146.2 (q, C-3), 139.2 (t, C-2), 137.0 (q, C-7), 131.2 (q, C-11), 128.5 (t, C-6), 125.2 (t, C-10), 116.3 (s, C-13), 113.3 (s, C-1), 70.0 (s, C-14), 57.6 (p, C-OMe), 35.9 (s, C-8), 32.0 (s, C-4), 27.5 (s, C-9), 26.7 (s, C-5), 25.9 (p, C-12), 17.8 (p, C-15) ppm; HRMS (CI-GC): *m/z calc.* for $C_{16}H_{26}O$ $[M]^{+}$: 234.1984; *found*: 234.1975; R_f (50:1 PE/EtOAc): 0.31.

Product 41

^{1}H-NMR (600 MHz, C_6D_6): δ = 6.42 (dd, *J* = 17.4 Hz, 10.7 Hz, 1H, H-2), 5.45 (t, *J* = 7.4 Hz, 1H, H-4), 5.35 (t, *J* = 7.8 Hz, 1H, H-6), 5.25 – 5.23 (m, 1H, H-10), 5.10 (d, *J* = 17.4 Hz, 1H, H-1), 4.96 – 4.94 (m, 1H, H-1), 3.89 (s, 2H, H-14), 3.11 (s, 3H, H-OMe), 2.85 (t, *J* = 7.4 Hz, 2H, H-5), 2.29 – 2.20 (m, 4H, H-8, H-9), 1.67 (s, 3H, H_{13}), 1.67 (s, 3H, H-12), 1.56 (s, 3H, H-15) ppm; ^{13}C-NMR (151 MHz, C_6D_6): δ = 141.9 (t, C-2), 137.2 (q, C-7), 134.5 (q, C-3), 131.5 (t, C-4), 131.4 (q, C-11),126.6 (t, C-6), 124.9 (t, C-10), 111.0 (s, C-1), 70.0 (s, C-14), 57.7 (p, C-OMe), 35.9 (s, C-8), 27.4 (s, C-9), 27.3 (s, C-5), 25.9 (p, C-12), 17.8 (p, C-15), 11.8 (p, C-13) ppm; HRMS (CI-GC): *m/z calc.* for $C_{16}H_{26}O$ $[M]^{+}$: 234.1984; *found*: 234.1991; R_f (50:1 PE/EtOAc): 0.31.

4. Molecular Modelling

All molecular docking studies were performed using MOE 2022.02.[S7] The 3D structures of hydroxy and methoxy derivatives of FPP (Structure **10** and **11**, respectively) were constructed in the online molecule designing platform MolView (https://molview.org/).[S8] The structures were downloaded as .mol file and opened in MOE and standard minimization was performed. The .pdb file containing the wild strain of BcBOT2 along with the substrate FPP **1** was gained from our previous study.[S9] It was compared to the recently published structure (PDB ID:8H6U) which was highly similar to our model (overall RMSD: 1.72Å), the active site residues having RMSD less than 1Å as seen in Figure S1. Prior to substrate docking, the active pocket was selected in the receptor molecule at the active site of BcBOT2 where FPP **1** was situated using Site Finder in MOE. It is in this site that the two derivatives were docked using Dock tool in MOE. For each of the ligands, 400 poses were taken into consideration according to London dG Score criteria of which the top 20 poses were selected for visualization on the basis of GBVI/WSA dG Score criteria. For both ligands, “Triangle Matcher” was chosen for placement, and the “Induced Fit” method was used for refinement of ligand poses, permitting the interaction of flexible side chains at the active site with the two substrates during docking.

The final docking pose for the two derivatives of FPP **10** and **11** were chosen from the 20 poses by comparing them to available pose of the substrate FPP **1** at the active site of BcBOT2 collected from our previous study. Priority was given to those poses that showed expected orientation of the pyrophosphate moiety of the substrates at the midst of the triad of Mg^{2+} ions at the coordination shell of the active site. These are ionic interactions. If a substrate was found with poses that form ionic interactions with only two of the three ions, but the positioning still resembled the original positions of FPP **1** at the active site, then this was taken into consideration. In such cases, additional components were included that might be favourable for the poses. In the final selection of the docking position for the two FPP derivatives, suitable orientations of the carbon component into the hydrophobic part of the active pocket were also considered, namely those that would allow interactions between the substrate and the amino acid residues at the active site. The binding affinity, volume and solvation of the final selected poses for the two derivatives **10** and **11** were automatically calculated after minimising the

steepest descent energy of the considered enzyme-substrate complex using the Ligand Properties tool. These could be visualised in the docking database of MOE. Comparisons of the ligand interaction diagrams of the final docked poses of the two FPP derivatives **10** and **11** with those of the substrate FPP **1** are shown in Figures S2 – S4.

The structures for the reaction intermediates from proposed pathway c was prepared accordingly using MOE builder from **11** to maintain their stereochemistry and searched for possible conformation while maintaining their respective stereochemistry. These conformers were then docked at the active site of $BcBOT_2$, 300 poses were taken into consideration according to London dG Score criteria of which the top 10 poses were selected for visualization on the basis of GBVI/WSA dG Score criteria and their results were analysed for catalytically competent poses.

A deeper understanding of the results obtained can be gained by comparing the substrate-BcBOT interactions of the final poses of the hydroxyl and methoxyl derivatives **10** and **11** from the substrate docking experiment with the available structure of FPP bound to the active site of BcBOT2 (see Table S 1).

Table S1: Comparison of the substrate-BcBOT2 interaction from molecular docking results between FPP and its hydroxyl and methoxyl derivatives. Highlighted are the major differences in interactions seen between the active site residues and the three substrates.

BcBOT2 residue	BcBOT2-Substrate Interactions		
	FPP	FPP-OH derivative	FPP-OMe derivative
LYS (292)	H – Bond	Ionic Bond	Ionic Bond
ASN (285)	H – Bond	H – Bond	None
ARG (373)	H – Bond /Ionic Bond	H – Bond /Ionic Bond	Ionic Bond
ARG (239)	H – Bond	H – Bond	H-Bond
MG (901)	Metal Bond	Metal Bond	Metal Bond
MG (902)	Metal Bond	Metal Bond	Metal Bond
MG (903)	Metal Bond	None	Metal Bond
ASP(293)	H-Bond	H-Bond	H-Bond
ASP(286)	H-Bond	H-Bond	H-Bond
PHE(138)	Hydrophobic	Hydrophobic	Hydrophobic
ILE(243)	Hydrophobic	Hydrophobic/ H-Bond	Hydrophobic

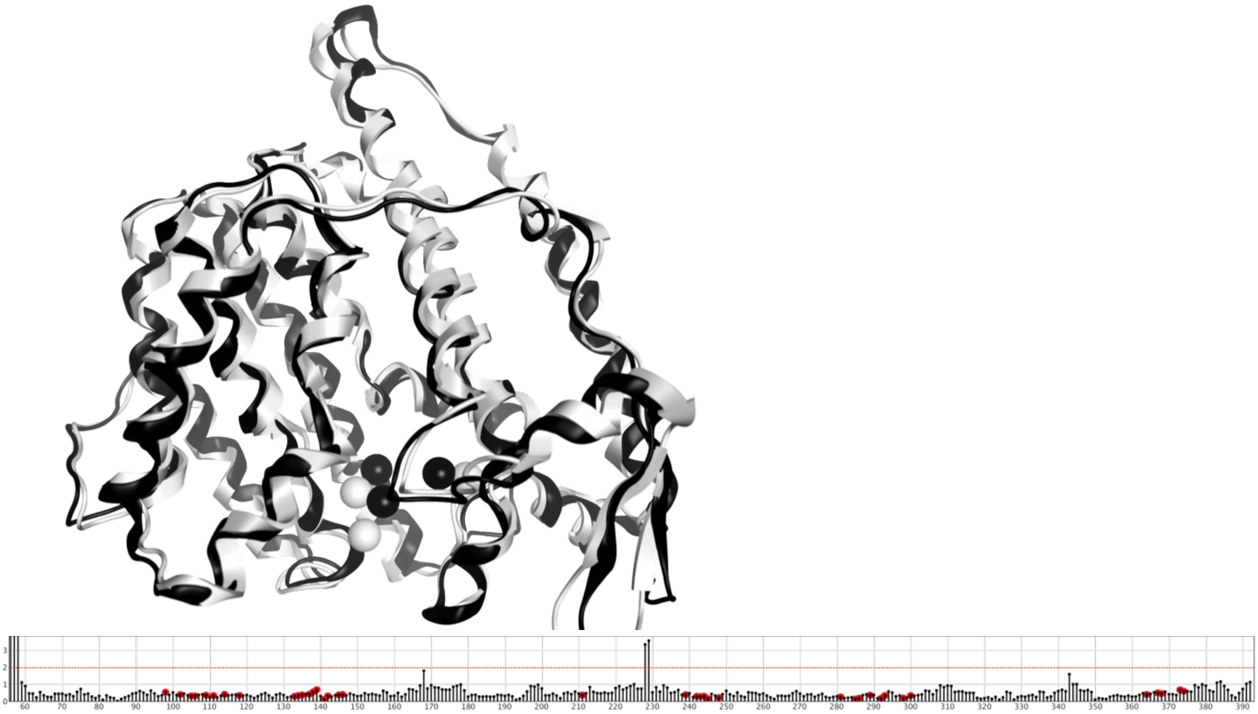

Figure S1: The superimposed structures of the Alphafold model of BcBOT**2** used in our investigation (in white) and the recently published structure from PDB structure 8H6U (in black), the overall RMSD being 1.72Å. The active site residues highlighted in red in below graph with RMSD less than 1Å shows that the predicted model has a highly similar active site to reported structure.

The docking pose of the methoxyl derivative **11** was more similar to that of FPP **1**. However, the presence of a smaller number of hydrogen bonds in the case of the methoxy derivative could be due to the larger steric influence of the methoxy group. In contrast, the hydroxyl derivative **10** showed a greater number of interactions with active site residues compared to FPP **1**. The additional hydroxyl group was highly interactive with neighbouring amino acid residues of the binding pocket. None of the docking poses analyzed after docking for the hydroxyl derivative of FPP **10** showed the ionic interaction between the triad of Mg^{2+} ions and the pyrophosphate moiety of the substrates. A maximum ionic interaction was observed with two of the three Mg^{2+} ions. This could be due to the conformational changes caused by the presence of the highly interactive hydroxyl group in the derivative. Although the hydroxyl derivative remained completely in the binding pocket, it is clear from Figure 1 (main text) that the interactions due to the hydroxyl group could orient the substrate at the active site boundary towards the opposite position of the triad of Mg^{2+} ions. Docking results from the reaction intermediates were analysed for top catalytically competent pose.

In silico site saturation mutagenesis was conducted in MOE on residues As285 and Arg373, which were hypothesized to promote the formation of **26**. Mutagenesis was carried out on these two residues using variations encompassing twenty standard amino acids. The stability of each variant, as well as its affinity towards **26** having 25 conformations with same stereochemistry as **26** were studied. The results of the change in stability and the affinity with the conformer having the lowest binding energy are shown in Table S2. Further visualization of all the conformers of **26** with the stable variants were performed to investigate the interactions between **26** and the neighbouring amino acid residues at the active site of their respective variants as shown in Figure S6.

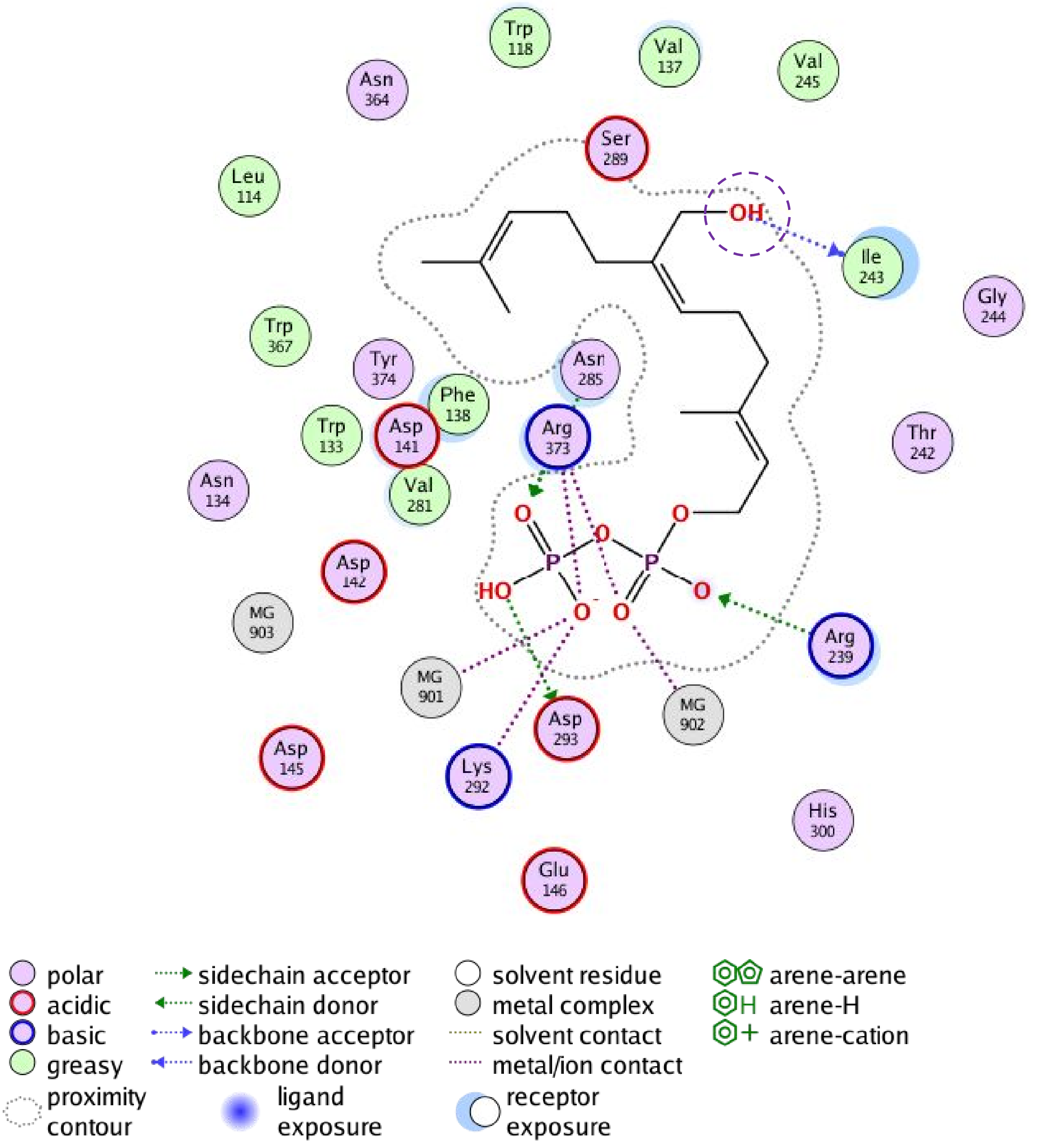

Figure S2: 2D representation of $BcBOT_2$-substrate interaction between the FPP derivative **10** bearing an additional hydroxy group and the residues present at the active site of $BcBOT_2$. The substituted group is marked with a violet dotted circle. Note: This is a 2D representation and the arrangement in 3D is more of a helical nature. The narrow cavity at the $BcBOT_2$-FPP binding pocket has the triad of Mg^{2+} ions and other hydrophilic (acidic and basic) residues like Asp, Arg and Lys at one end of the binding pocket, while the other hydrophobic residues are at the opposite end. Additionally, any orientation that favors a non-linear positioning in the cavity loses the vital interaction between the -OPP subgroup and the triad of Mg^{2+} ions. Without this interaction, the enzymatic reaction would not proceed further.

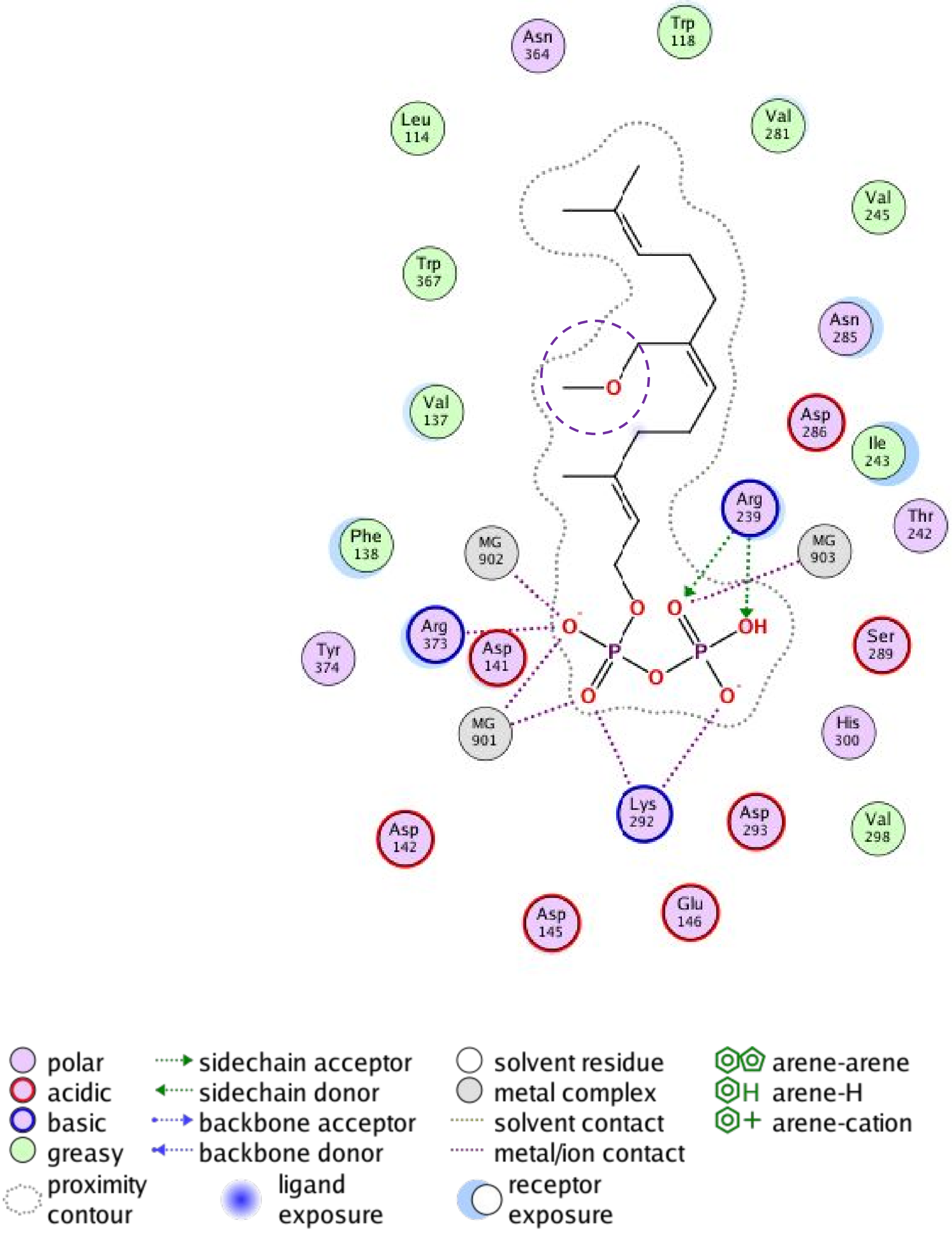

Figure S3: 2D representation of BcBOT$_2$-substrate interaction between the FPP derivative **11** bearing an additional methoxy group and the residues present at the active site of BcBOT$_2$. The substituted group is marked with a violet dotted circle. Note: The narrow cavity at the BcBOT$_2$-FPP binding pocket has the triad of Mg^{2+} ions and other hydrophilic (acidic and basic) residues like Asp, Arg and Lys at one end of the binding pocket, while the other hydrophobic residues are at the opposite end. Additionally, any orientation that favors a non-linear positioning in the cavity loses the vital interaction between the -OPP subgroup and the triad of Mg^{2+} ions. Without this interaction, the enzymatic reaction would not proceed further. Only after the reaction is complete the side will be open for cyclisation at the opposite end.

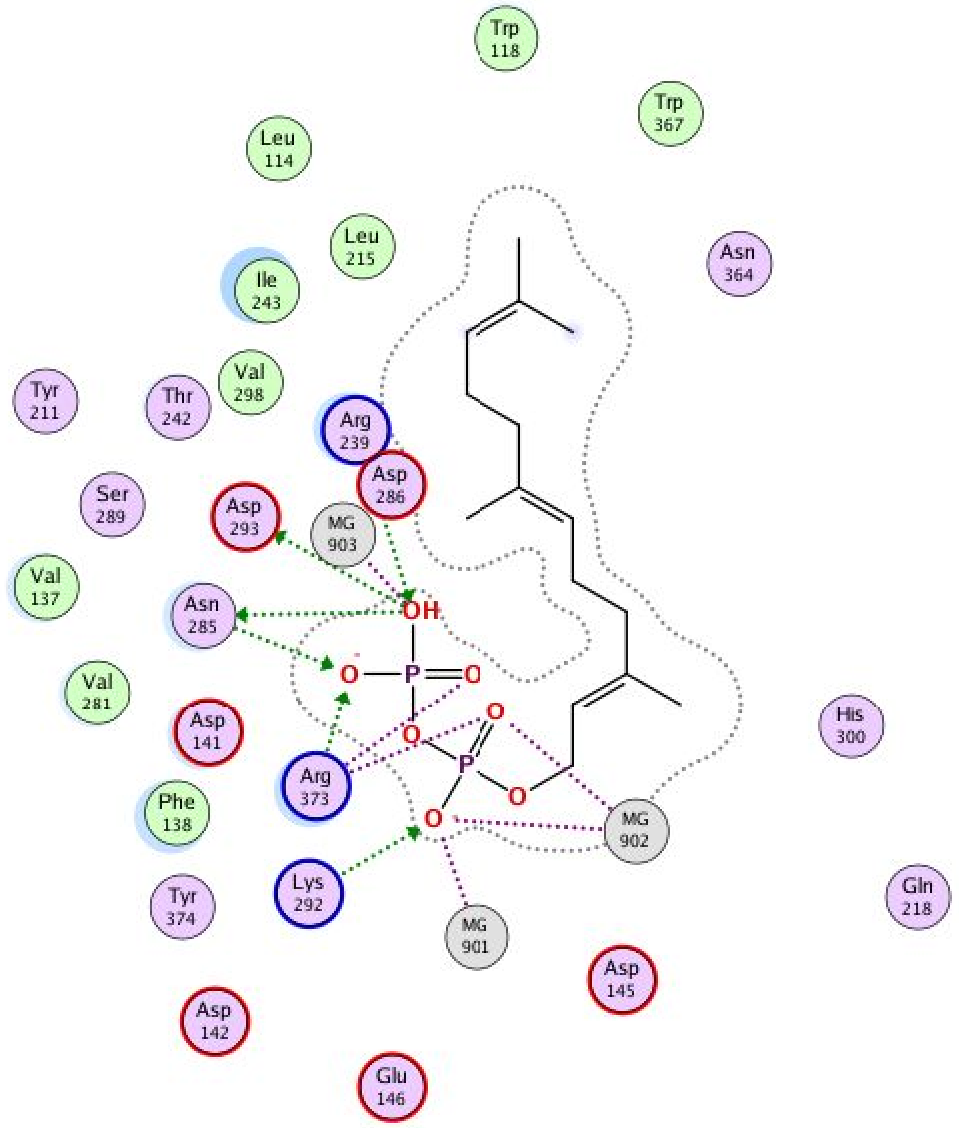

Figure S4: 2D representation of $BcBOT_2$-substrate interaction between FPP **1** and the residues present at the active site of $BcBOT_2$ are visualized. Note: This is a 2D representation and the arrangement in 3D is more of a helical nature. The narrow cavity at the BcBOT2 -FPP binding pocket has the triad of Mg^{2+} ions and other hydrophilic (acidic and basic) residues like Asp, Arg and Lys at one end of the binding pocket, while the other hydrophobic residues are at the opposite end. Additionally, any orientation that favors a non-linear positioning in the cavity loses the vital interaction between the -OPP subgroup and the triad of Mg^{2+} ions. Without this interaction, the enzymatic reaction would not proceed further. Only after the reaction is complete the side will be open for cyclisation at the opposite end.

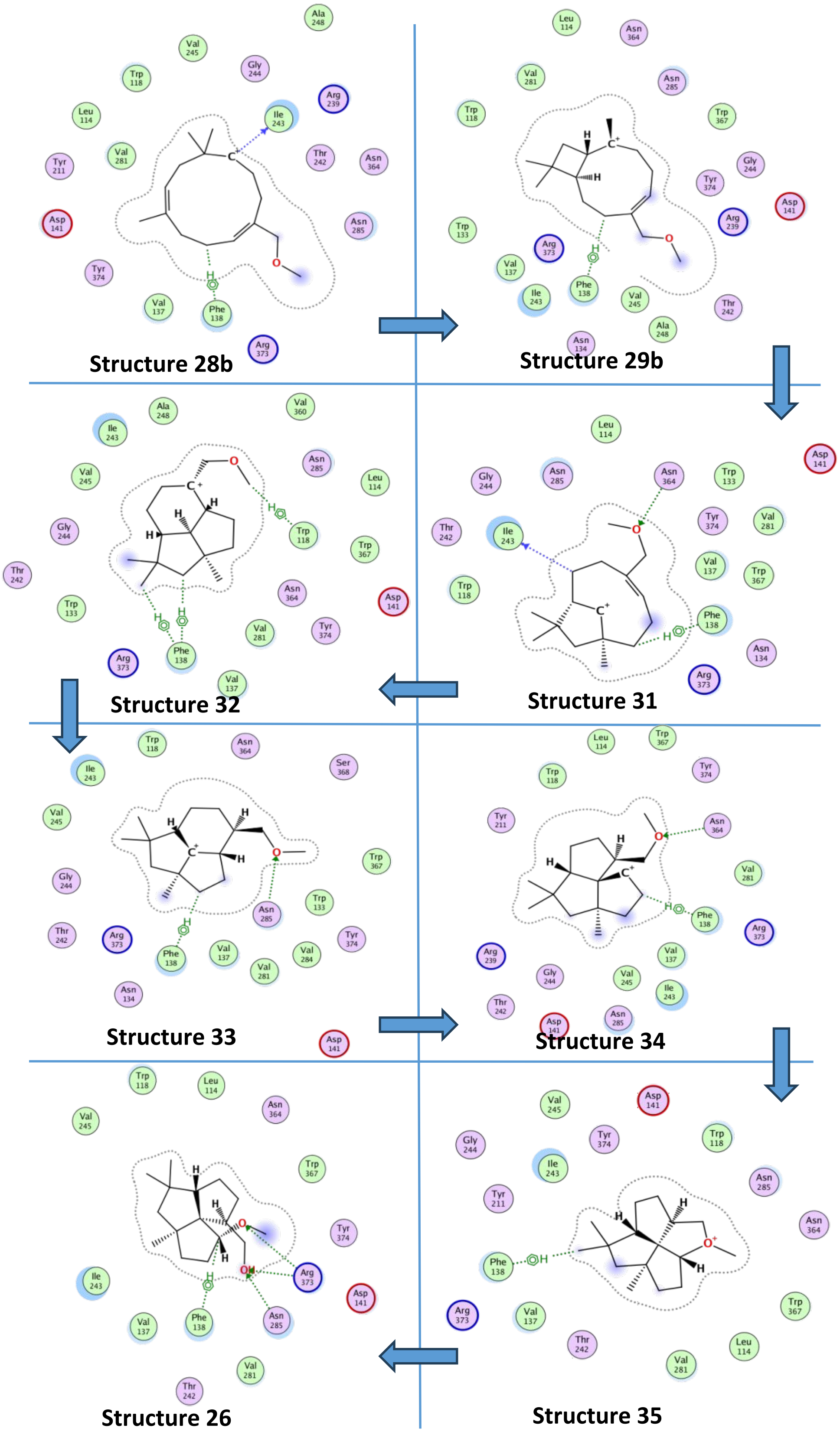

Figure S5: 2D representation of interactions observed between the top scoring catalytically competent pose of the reaction intermediates proposed for pathway c for the formation of **26** and the neighboring active site residues shown sequentially.

Table S2: Evaluation of the change in variant stability and affinity for **26** from the *in Silico* site saturation mutagenesis of positions As285 and Arg373. Variants are shaded on a gradient scale from red to blue, where red indicates lower stability and affinity towards compound **26**, while blue indicates higher stability and affinity. Stabilizing variants identified in the investigation are highlighted in a green box. The reported dAffinity is the conformer with the lowest binding free energy investigated from the 25 conformers of **26** while keeping the stereochemistry remains same.

mutation	dAffinity	dStability	mutation	dAffinity	dStability
**N285N**	0	0	**R373R**	0	0
**N285A**	0.192862	1.437169	**R373A**	0.996656	1.935293
**N285R**	**-0.45645**	**-0.41791**	**R373N**	0.750953	2.626298
**N285D**	-0.29295	1.220889	**R373D**	1.19175	2.828995
**N285C**	-0.1206	0.636643	**R373C**	0.657749	2.060768
**N285Q**	-0.09948	1.138822	**R373Q**	0.960653	2.159169
**N285E**	-0.46947	1.215011	**R373E**	1.042217	3.023234
**N285G**	0.099366	1.287113	**R373G**	0.639826	2.960276
**N285H**	-0.17488	1.223737	**R373H**	0.853496	2.605712
**N285I**	-0.21463	0.576197	**R373I**	1.099725	1.595639
**N285L**	-0.43254	0.535027	**R373L**	1.062045	2.378316
**N285K**	-0.05837	0.664321	**R373K**	0.696134	2.164401
**N285M**	-0.46043	0.374447	**R373M**	0.878305	2.288105
**N285F**	**-0.35643**	**-0.48051**	**R373F**	0.961261	2.165577
**N285P**	-0.00763	1.184305	**R373P**	1.070976	2.807532
**N285S**	0.033259	0.625649	**R373S**	1.063345	2.292498
**N285T**	0.058994	0.365981	**R373T**	1.3601	2.405527
**N285W**	**-0.77344**	**-0.40892**	**R373W**	0.216158	1.501527
**N285Y**	**-0.45032**	**-0.18786**	**R373Y**	0.993815	2.070112
**N285V**	0.11455	0.613379	**R373V**	1.103809	1.752644

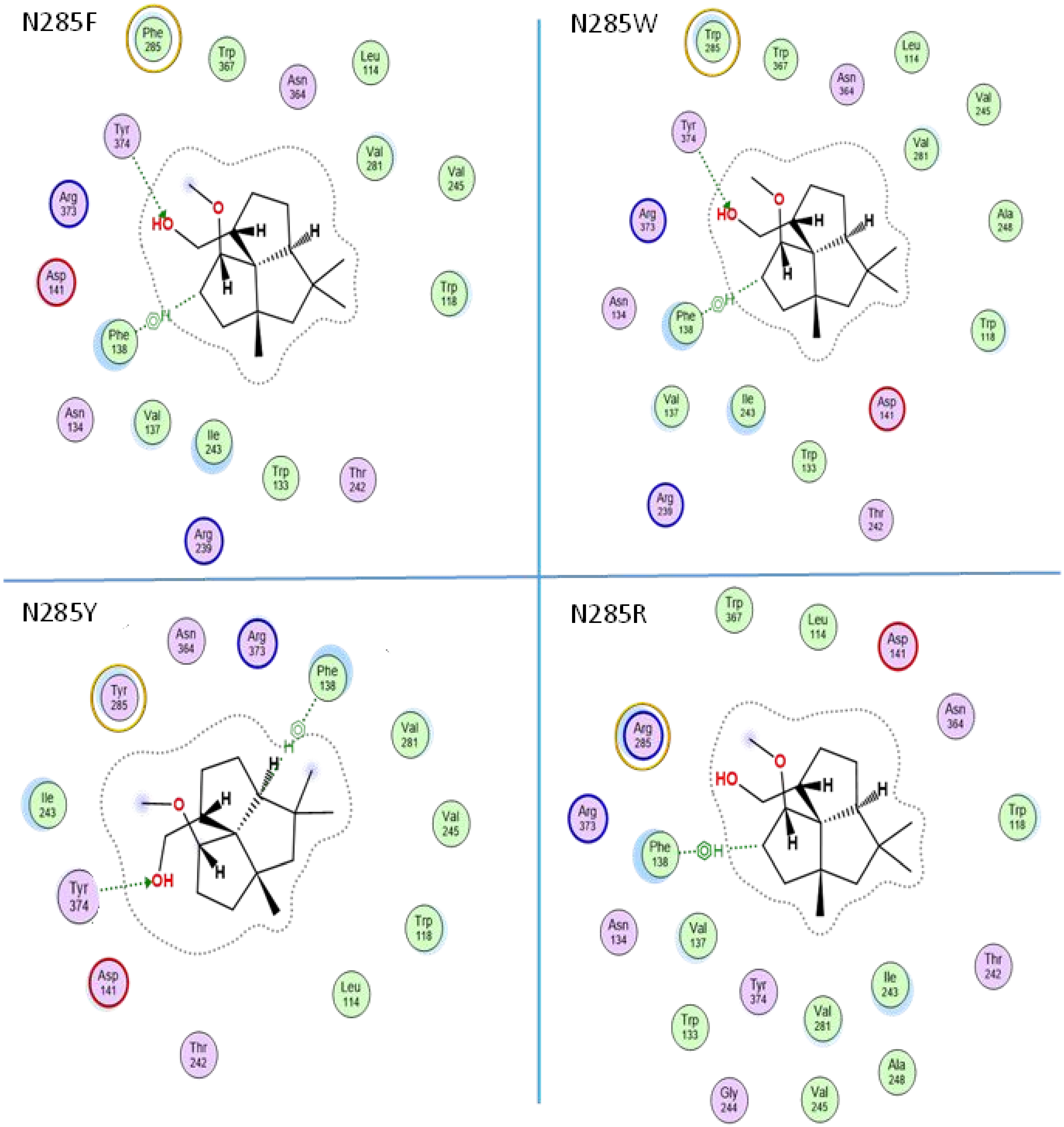

Figure S6: 2D representation of interactions observed between the **26** in the stable variants of Asn285- N285F, N285W, N285Y and N285R with the active site residues. The substituted residues in each of the variants are highlighted in yellow circle.

5. Xray analysis of 25 and 26

5.1 Xray analysis of 25

Experimental. Single colorless needle-shaped crystals of **25** were crystallized by evaporation from n-pentane. A suitable crystal 0.91×0.23×0.10 mm^3 was selected and mounted on a 18 mm mounted CryoLoop (20 micron, 0.2 - 0.3 mm, Hampton Research) on an XtaLAB AFC12 (RINC): Kappa single diffractometer. The crystal was kept at a steady T = 100.00(10) K during data collection. The structure was solved with the ShelXT[S10] structure solution program using the Intrinsic Phasing solution method and by using **Olex2**[S11] as the graphical interface. The model was refined with version 2019/3 of ShelXL 2019/3[S12] using Least Squares minimization. All non-hydrogen atoms were refined anisotropically. Hydrogen atom positions were calculated geometrically and refined using the riding model. Most hydrogen atom positions were calculated geometrically and refined using the riding model, but some hydrogen atoms were refined freely.

Crystal Data. $C_{16}H_{28}O_2$, M_r = 252.38, orthorhombic, $P2_12_12_1$ (No. 19), a = 6.80138(17) Å, b = 12.1469(3) Å, c = 17.9880(4) Å, $\alpha = \beta = \gamma = 90°$, V = 1486.10(6) Å^3, T = 100.00(10) K, Z = 4, Z' = 1, μ(Cu K$_\alpha$) = 0.556, 22734 reflections measured, 3176 unique (R_{int} = 0.0481) which were used in all calculations. The final wR_2 was 0.0860 (all data) and R_1 was 0.0329 (I > 2(I)).

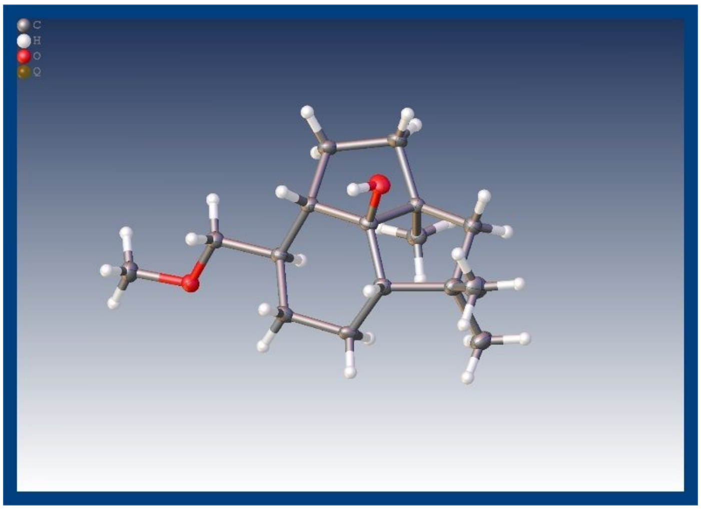

CDCC 2335733 contain the supplementary crystallographic data for this structure. These data can be obtained free of charge via www.ccdc.cam.ac.uk/structures/

5.2 Xray analysis of 26

Experimental. Single colorless needle-shaped crystals of **26** were obtained by evaporation from n-pentane. A suitable crystal 0.85×0.15×0.10 mm3 was selected and mounted on a 18 mm mounted CryoLoop (20 micron, 0.2 - 0.3 mm, Hampton Research) on an XtaLAB AFC12 (RINC): Kappa single diffractometer. The crystal was kept at a steady T = 100(2) K during data collection. The structure was solved with the ShelXT[S10] structure solution program using the Intrinsic Phasing solution method and by using **Olex2**[S11] as the graphical interface. The model was refined with version 2019/3 of ShelXL 2019/3[S12] using Least Squares minimization. All non-hydrogen atoms were refined anisotropically. Hydrogen atom positions were calculated geometrically and refined using the riding model. Most hydrogen atom positions were calculated geometrically and refined using the riding model, but some hydrogen atoms were refined freely.

Crystal Data. $C_{16}H_{28}O_2$, M_r = 252.38, orthorhombic, $P2_12_12_1$ (No. 19), a = 8.65260(10) Å, b = 13.3873(2) Å, c = 51.7237(7) Å, $a = b = g = 90°$, V = 5991.41(14) Å^3, T = 100(2) K, Z = 16, Z' = 4,

m(CuK$_a$) = 0.551, 62438 reflections measured, 12710 unique (R_{int} = 0.0337) which were used in all calculations. The final wR_2 was 0.0981 (all data) and R_1 was 0.0372 (I > 2(I)).

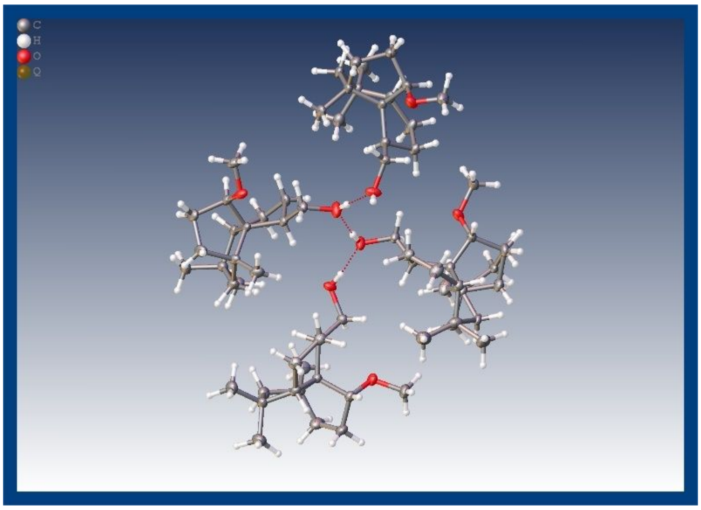

CDCC2335779 contain the supplementary crystallographic data for this structure. These data can be obtained free of charge via www.ccdc.cam.ac.uk/structures/

6. Supporting Information References

[S1] C. Oberhauser, V. Harms, K. Seidel, B. Schröder, K. Ekramzadeh, S. Beutel, S. Winkler, L. Lauterbach, J. S. Dickschat, A. Kirschning, *Angew. Chem. Int. Ed.* **2018**, *57*, 11802–11806.
[S2] N. S. George, K. E. Anderson, A. G. M. Barrett, *European J. Org. Chem.* **2013**, 7604–7610.
[S3] J. Rinkel, P. Rabe, P. Garbeva, J. S. Dickschat, *Angew. Chem. Int. Ed.* **2016**, *55*, 13593–13596.
[S4] S. Sommer, M. Kühn, H. Waldmann, *Adv. Synth. Catal.* **2008**, *350*, 1736–1750.
[S5] D. J. Miller, F. Yu, D. W. Knight, R. K. Allemann, *Org. Biomol. Chem.* **2009**, *7*, 962–975.
[S6] A. Gromov, Studies towards the Total Synthesis of Branimycin, Universität Wien, **2009**.
[S7] Chemical Computing Group, I., Molecular operating environment (MOE). **2016**, Chemical Computing Group Inc. Montreal, QC, Canada.
[S8] H. Bergwerf, *MolView,* **2018**. Available from: https://buildmedia.readthedocs.org/media/pdf/molview/latest/molview.pdf.
[S9] V. Nikolaiczyk, J. Irwan, T. Nguyen, J. Fohrer, P. Elbers, P. Schrank, M. D. Davari, A. Kirschning, Rational reprogramming of the sesquiterpene synthase BcBOT2 yields new terpenes with presilphiperfolane skeleton, *Catal. Sci. Technol.* **2023**, ***13***, 233 – 244.
[S10] Sheldrick, G. M. *Acta Cryst.* **2015**, *C71*, 3–8.
[S11] O.V. Dolomanov and L.J. Bourhis and R.J. Gildea and J.A.K. Howard and H. Puschmann, Olex2: A complete structure solution, refinement and analysis program, *J. Appl. Cryst.*, (2009), **42**, 339-341.
[S12] J. Lübben, C. M. Wandtke, C. B. Hübschle, M. Ruf, G. M. Sheldrick, B. Dittrich, *Acta Cryst.* **2019**, A*75*, 50–62.

Attachments
A. GC chromatograms and MS spectra of new terpenoids

Product **23** (t_r = 9.863 min, RI = 1591)

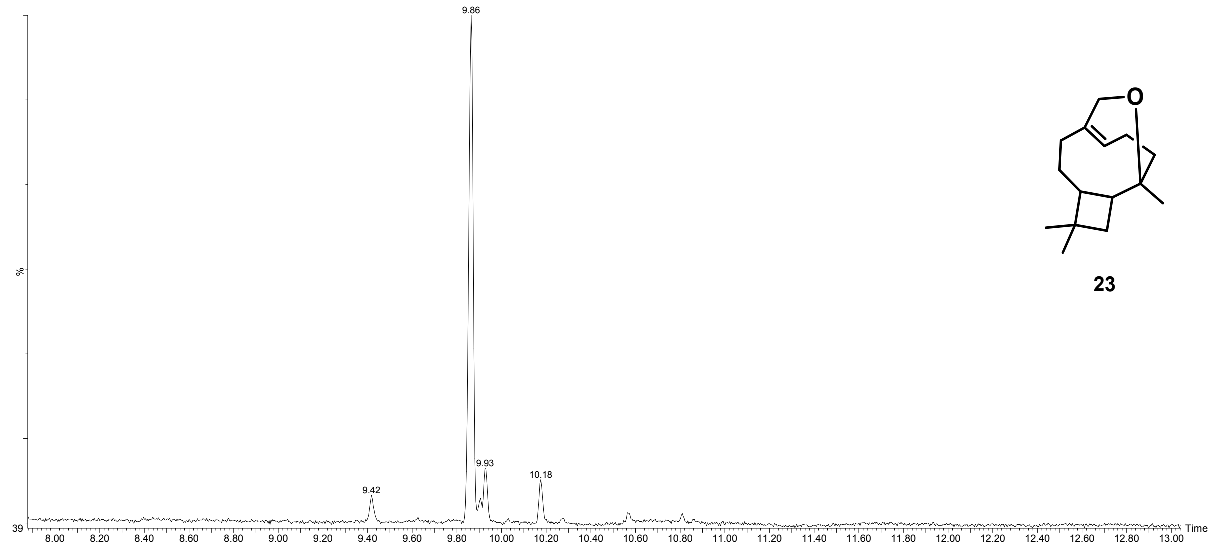

23

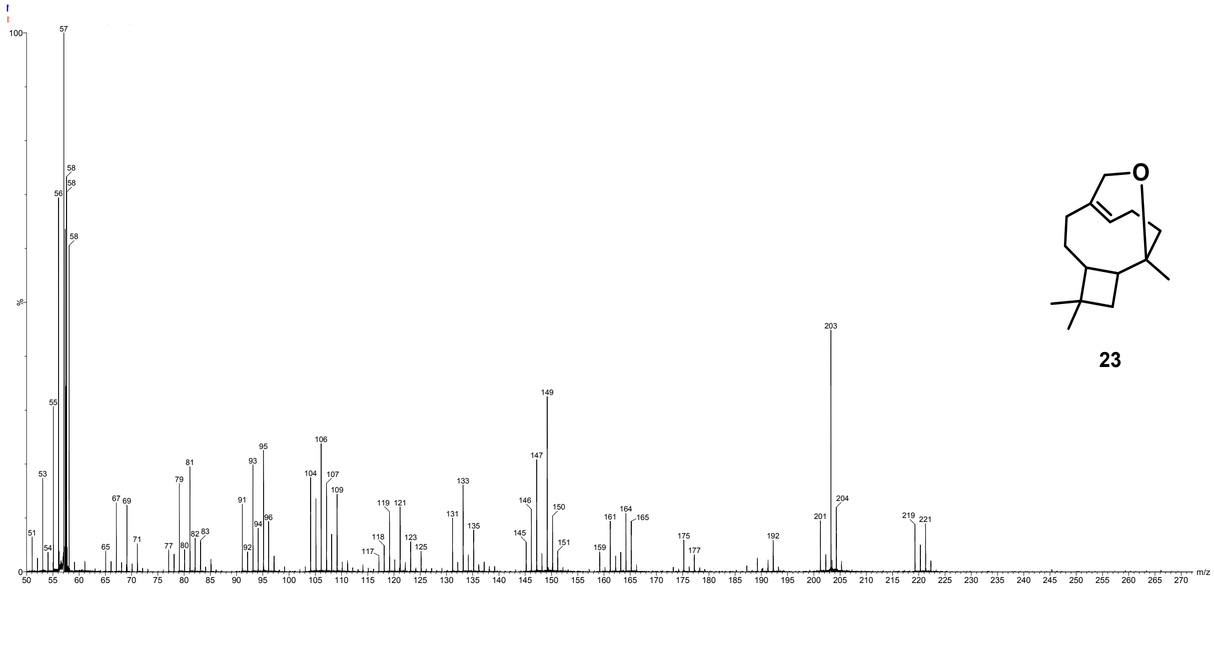

23

Product **24** (t_r =10.297 min , RI = 1639)

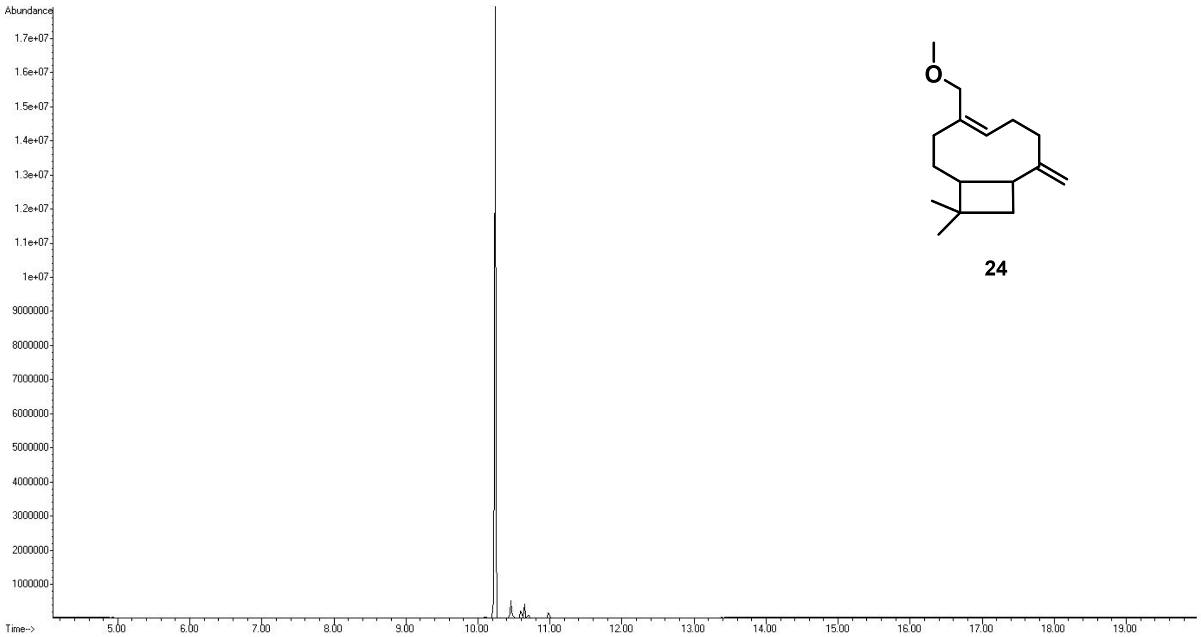

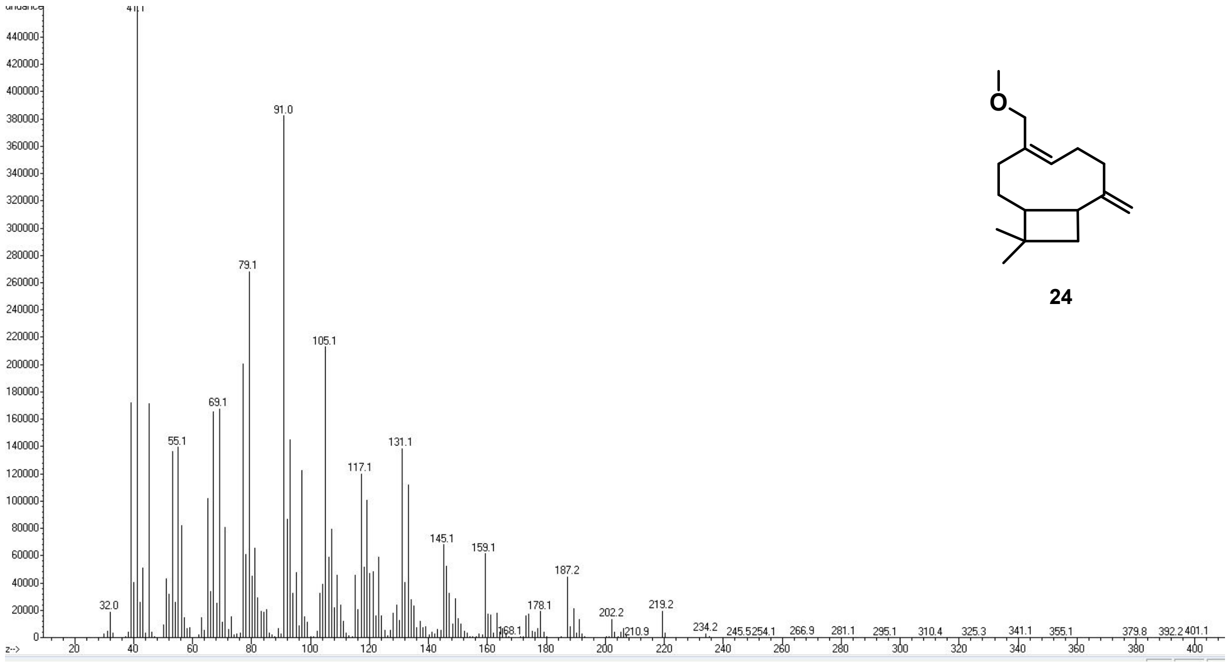

Product **25** (t_r = 11.305 min, RI = 1864):

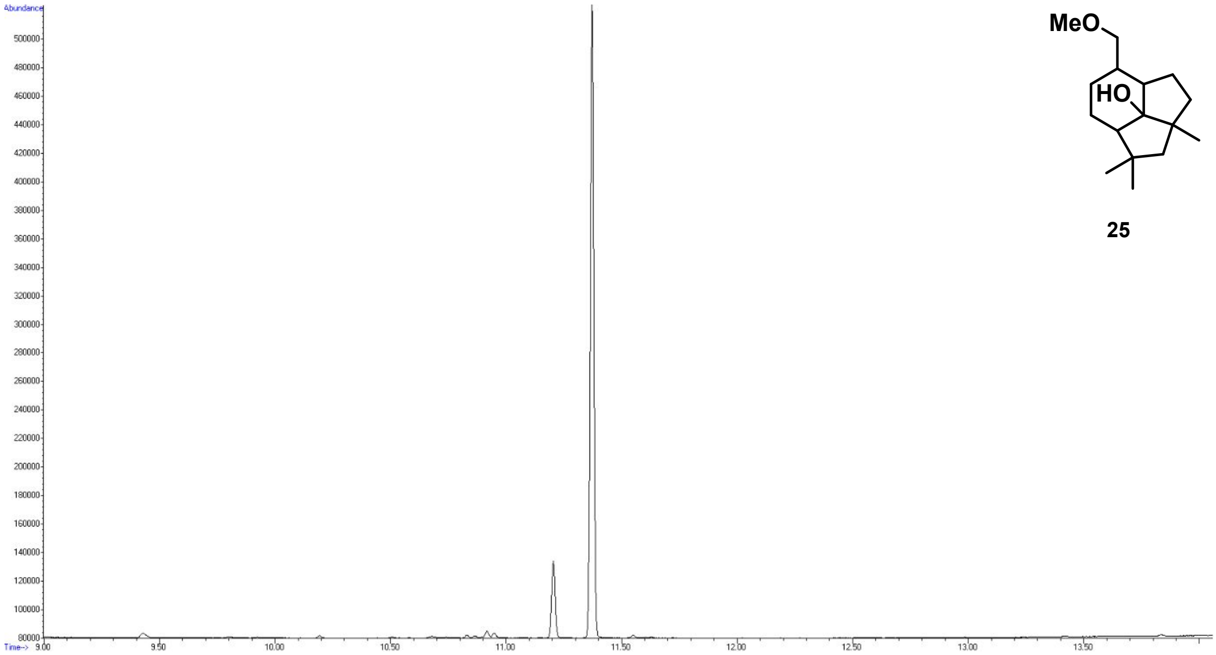

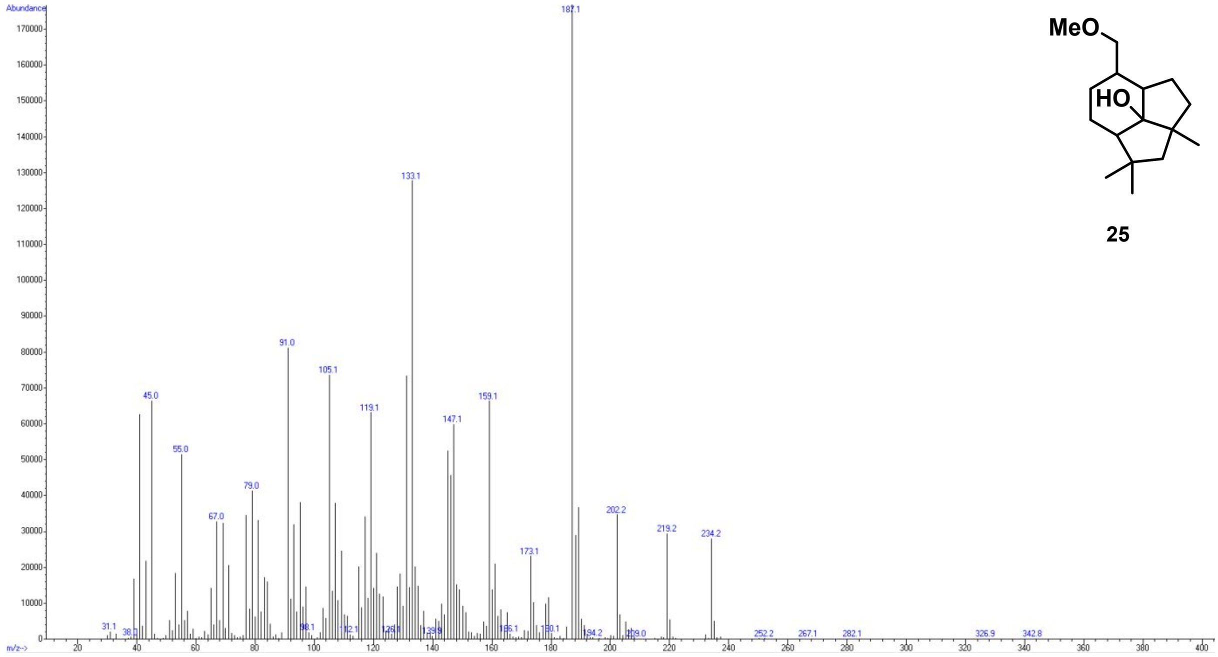

Product **26** (t_r = 11.155 min, RI = 1832)

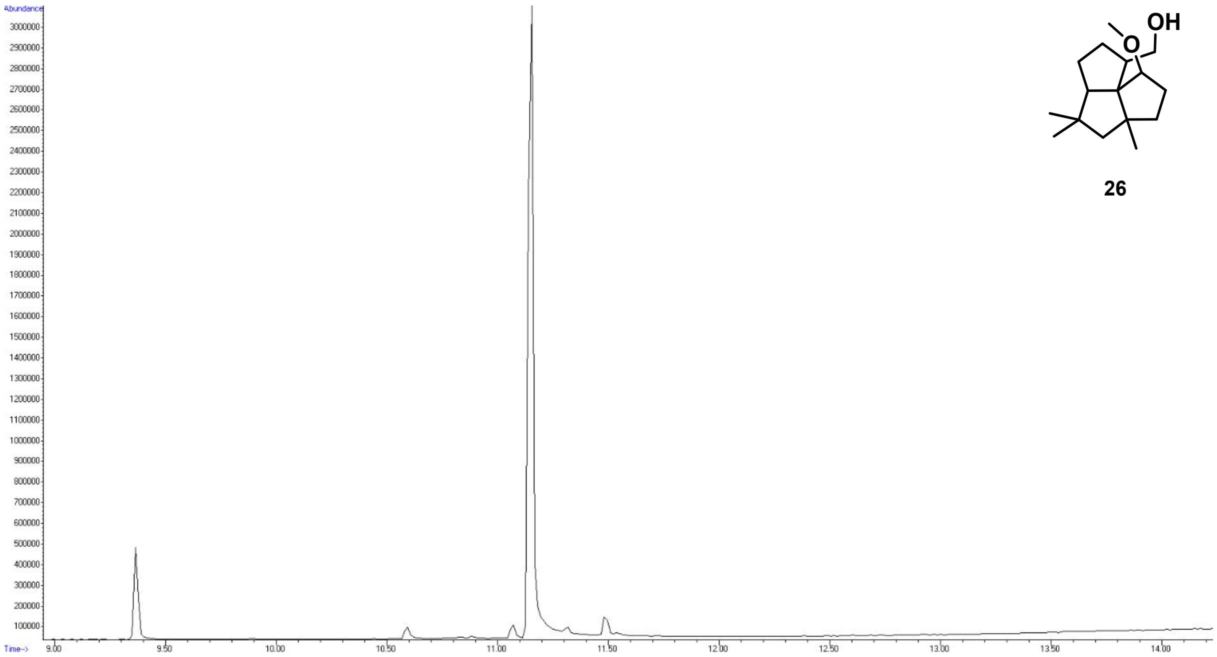

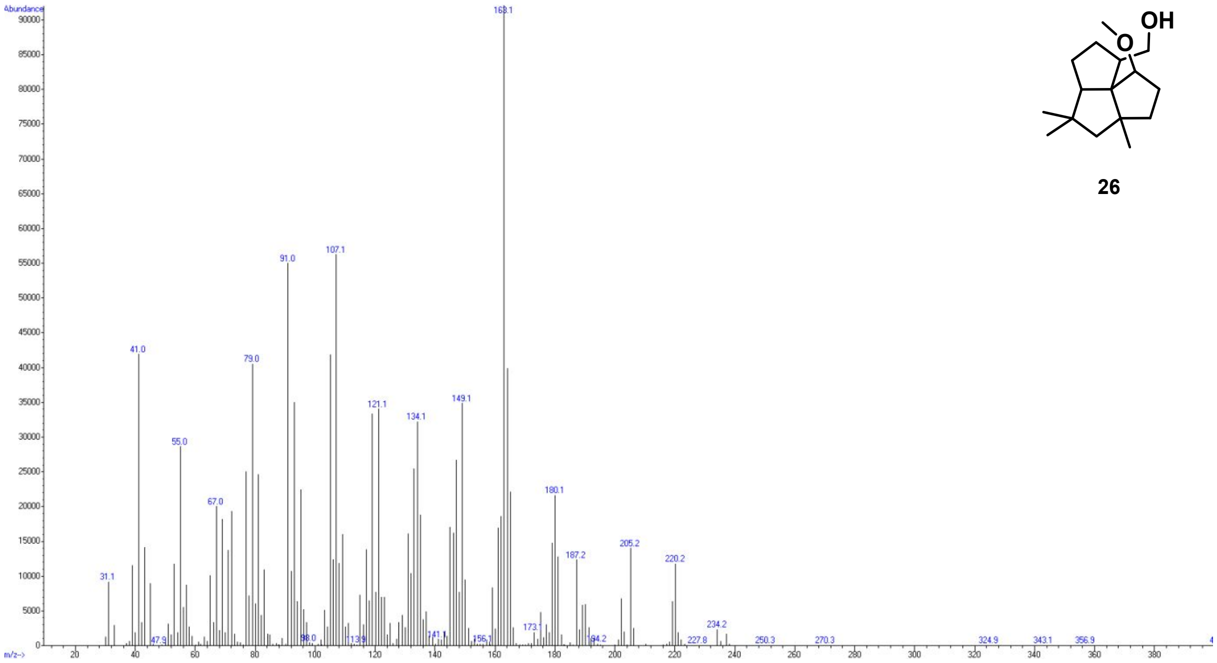

Product **40** and **41**(t_r = 10.207 min, RI = 1641, t_r = 10.424 min, RI = 1688)

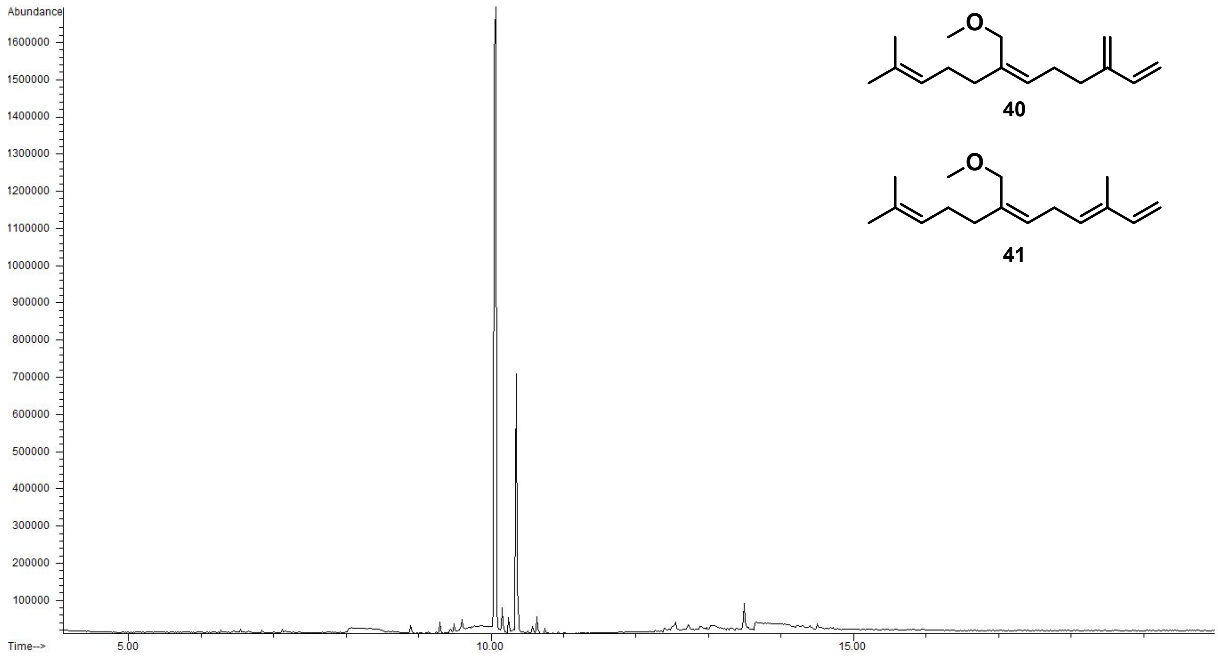

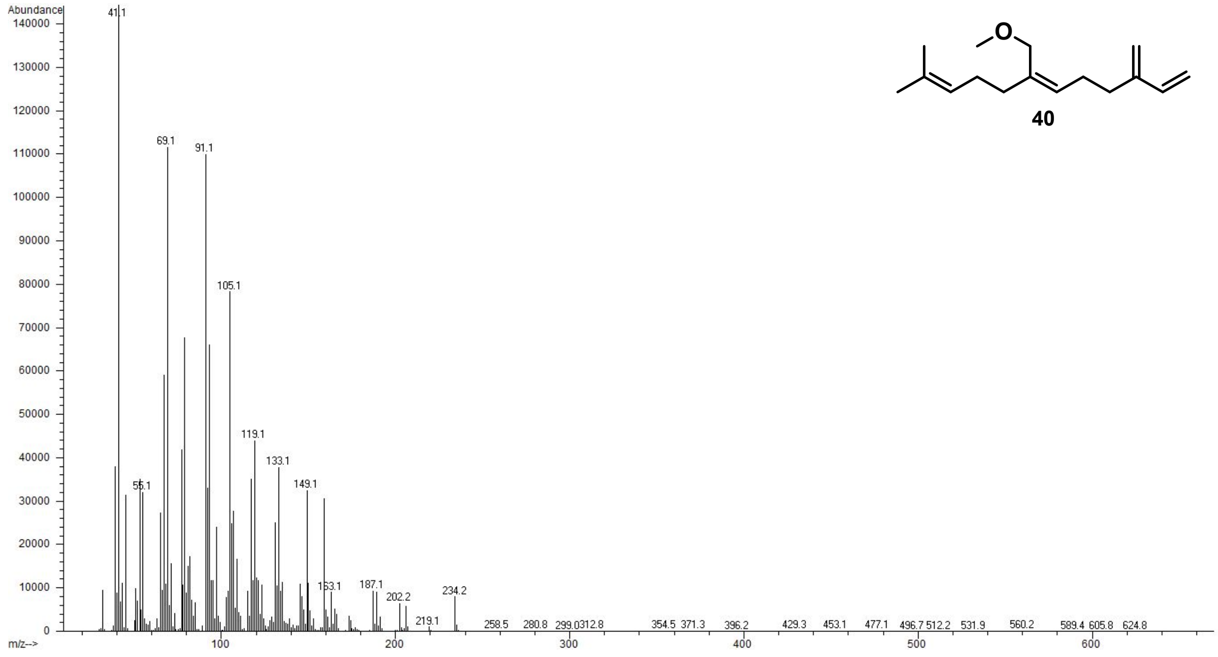

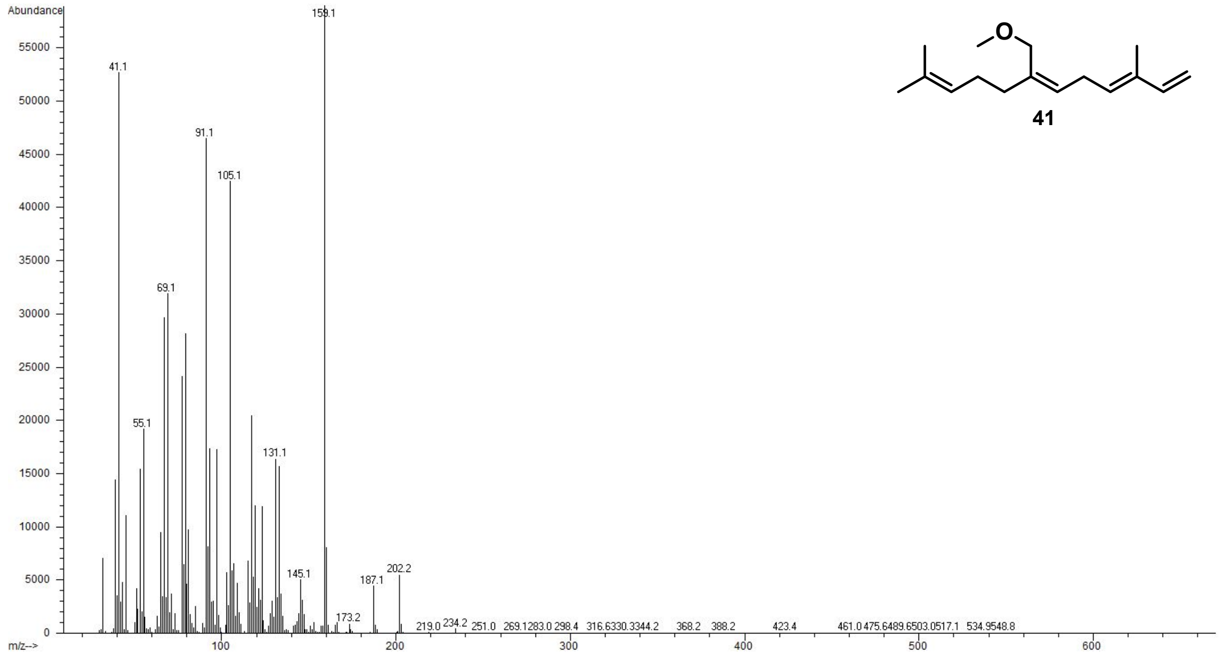
Abundance
55000
50000
45000
40000
35000
30000
25000
20000
15000
10000
5000
0
m/z-->
41.1
55.1
69.1
91.1
105.1
131.1
145.1
159.1
173.2
187.1
202.2
219.0 234.2 251.0 269.1283.0 298.4 316.6330.3344.2 368.2 388.2 423.4 461.0475.6489.6503.0517.1 534.9548.8
100
200
300
400
500
600
O

41

B. Copies NMR spectra of new terpenoids

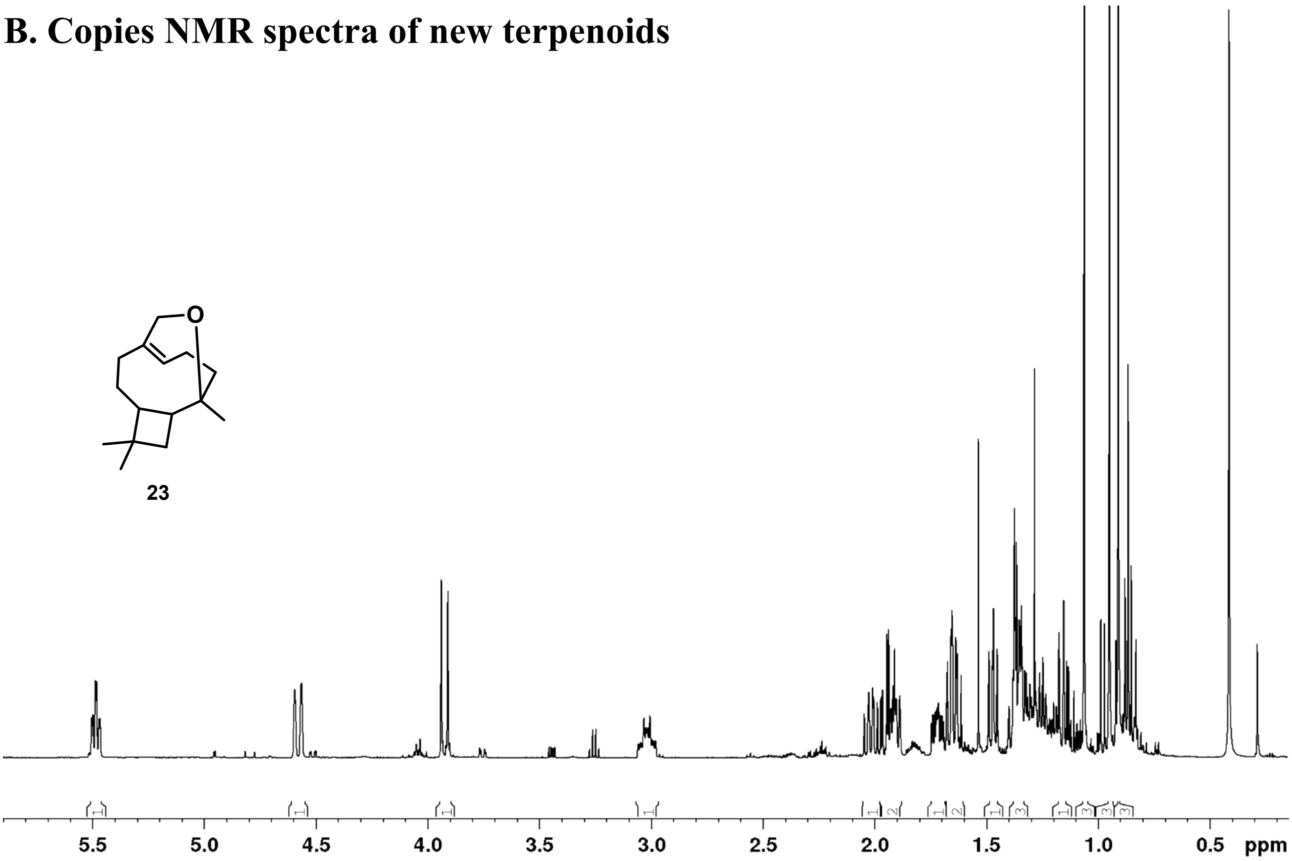

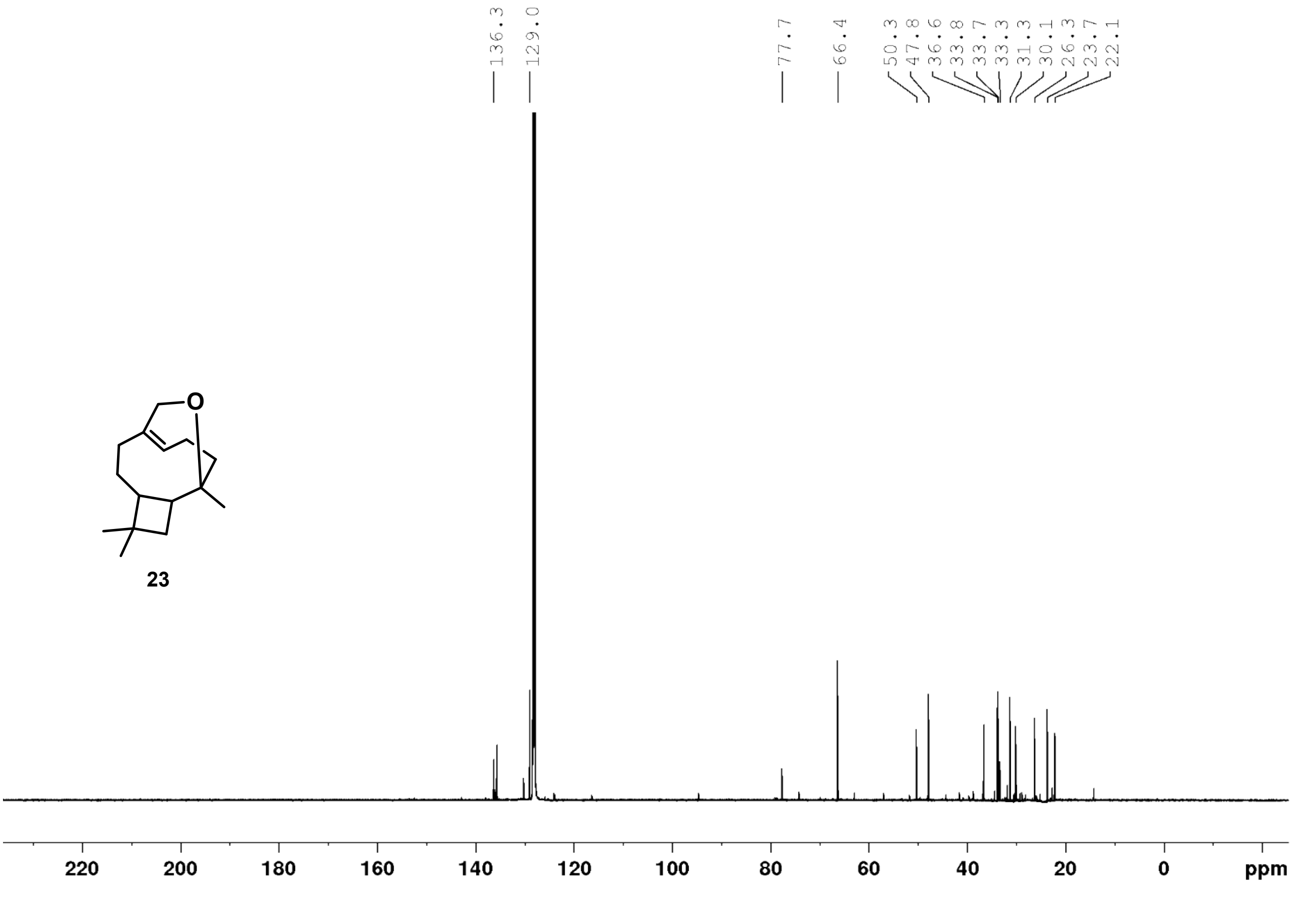

HSQC

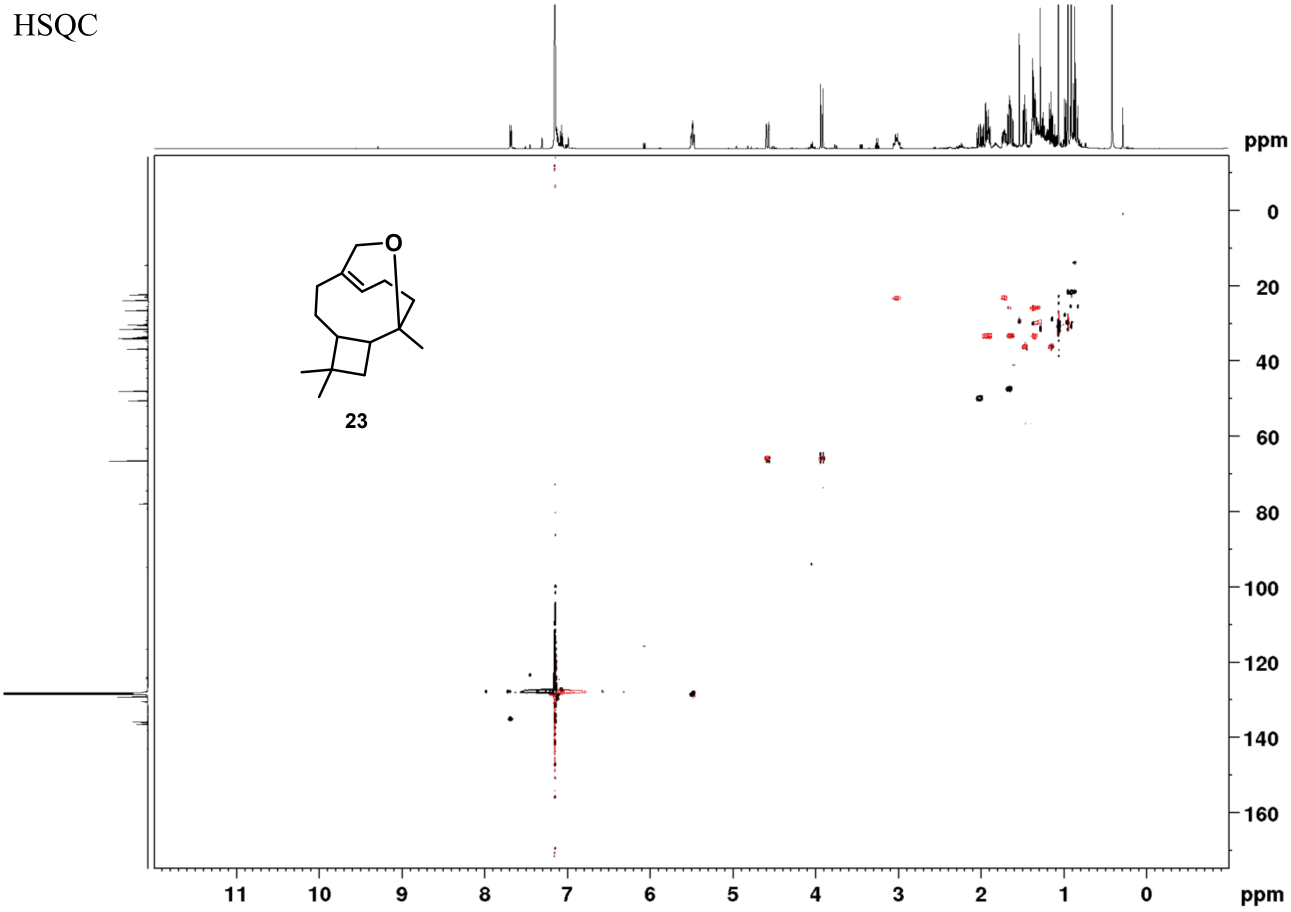

HMBC

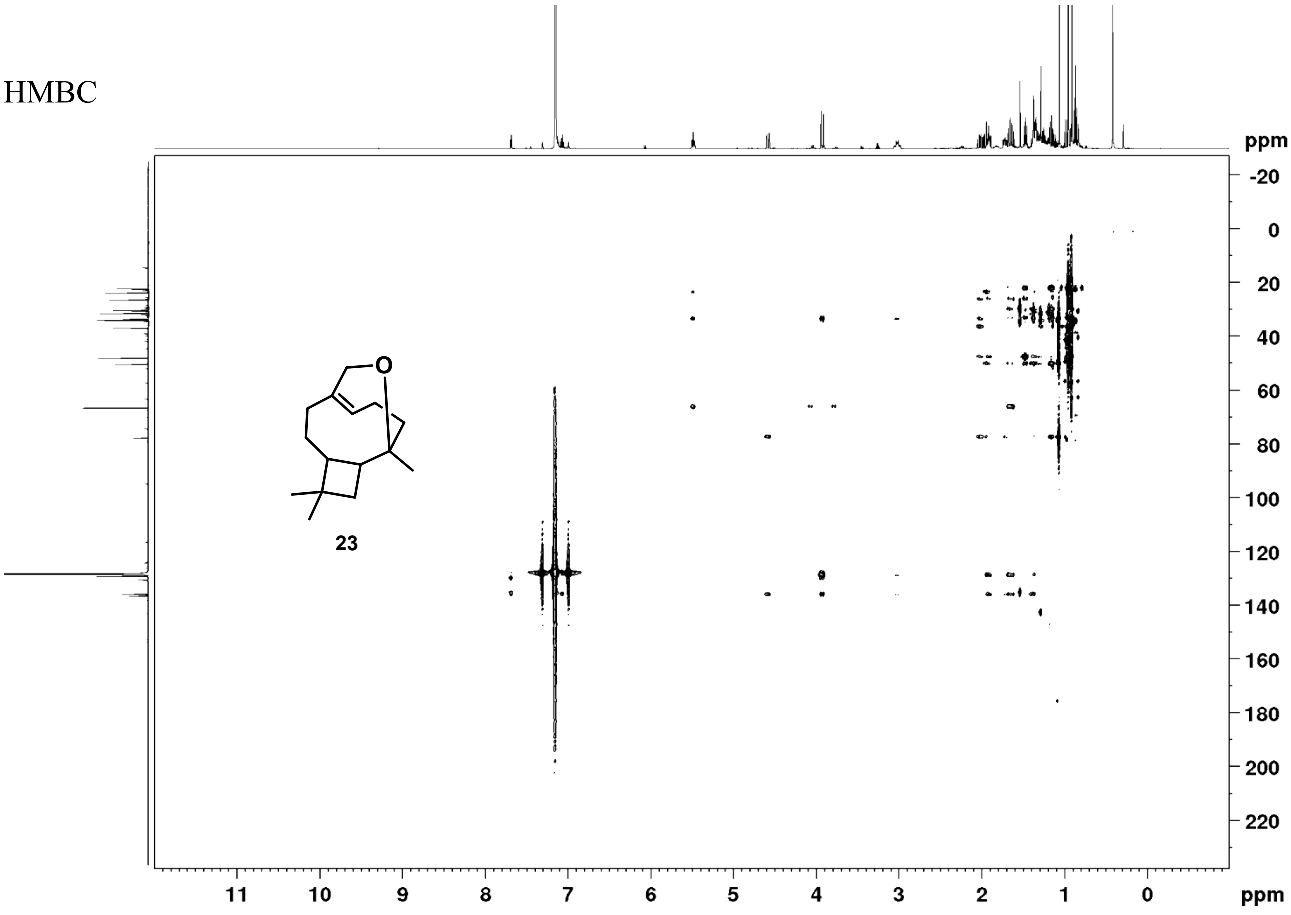

COSY

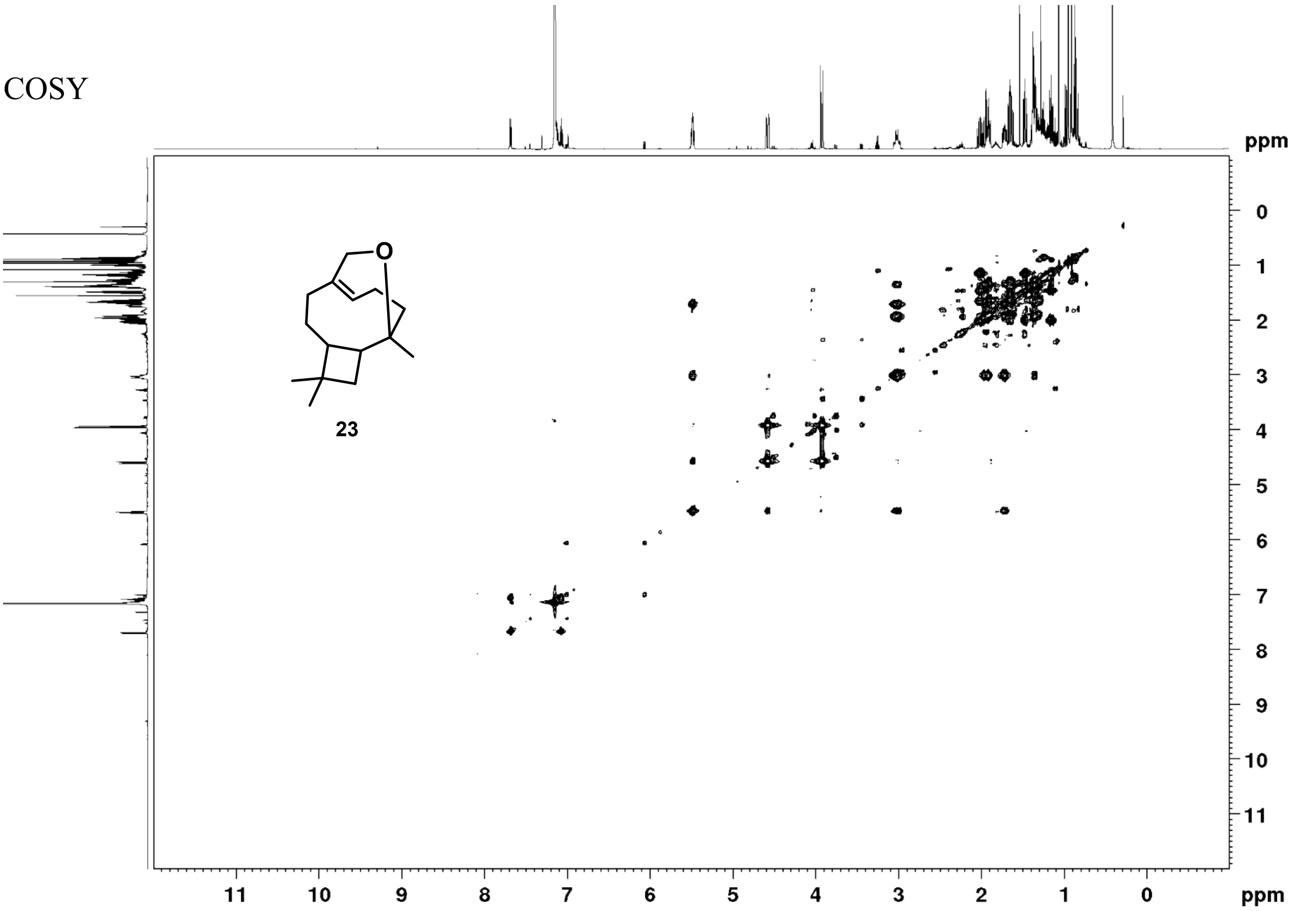

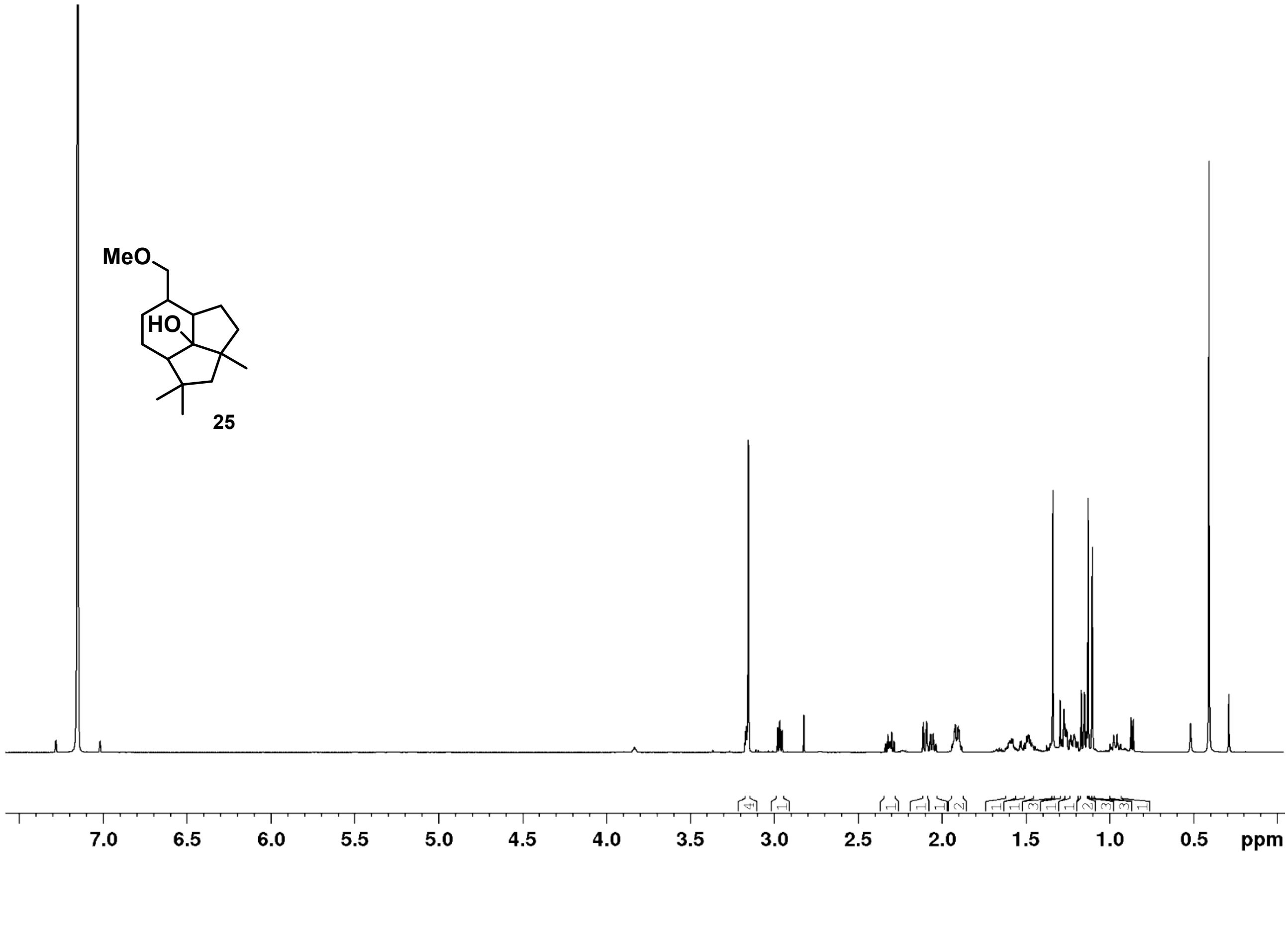

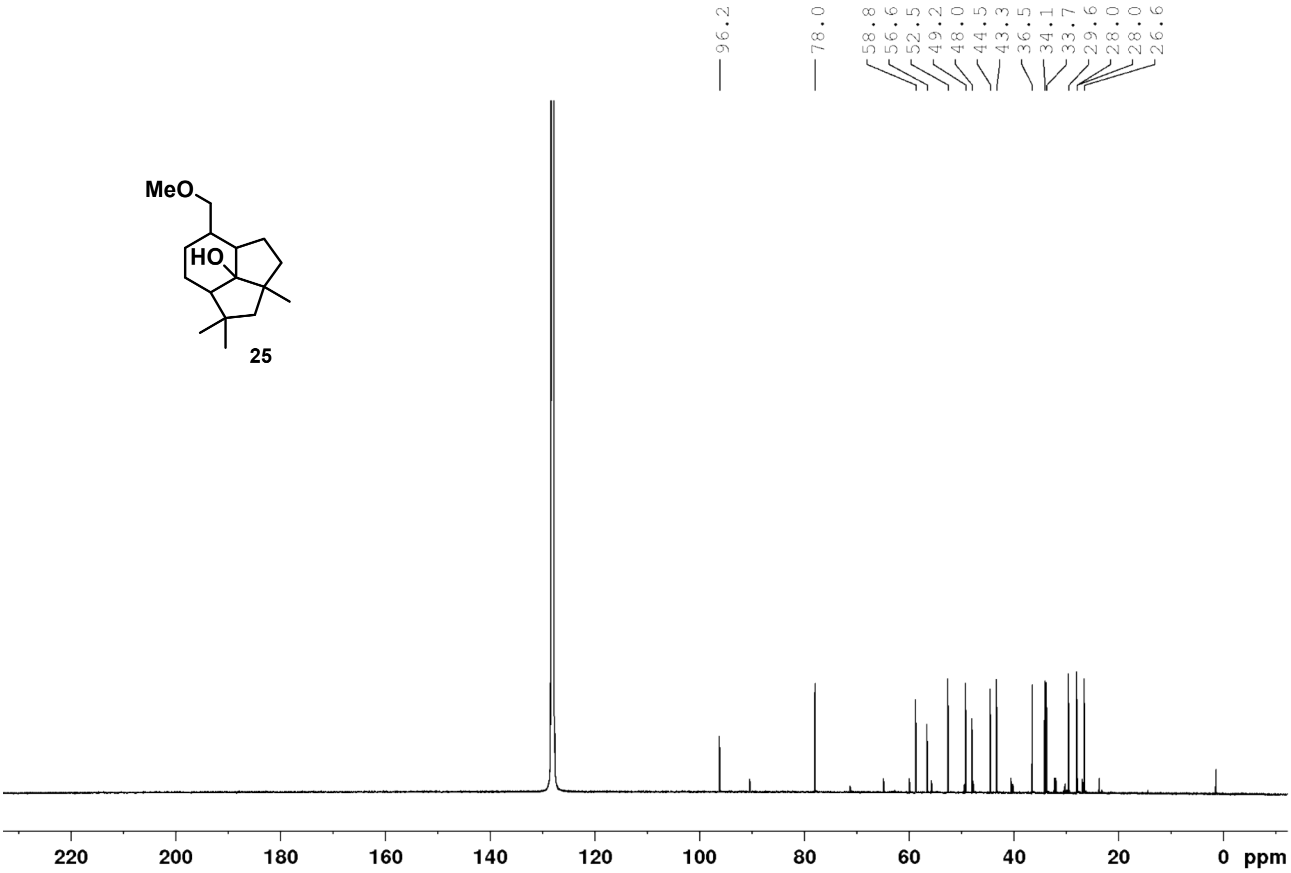
96.2
78.0
58.8
56.6
52.5
49.2
48.0
44.5
43.3
36.5
34.1
33.7
29.6
28.0
28.0
26.6
MeO
HO
25
220
200
180
160
140
120
100
80
60
40
20
0
ppm

HSQC

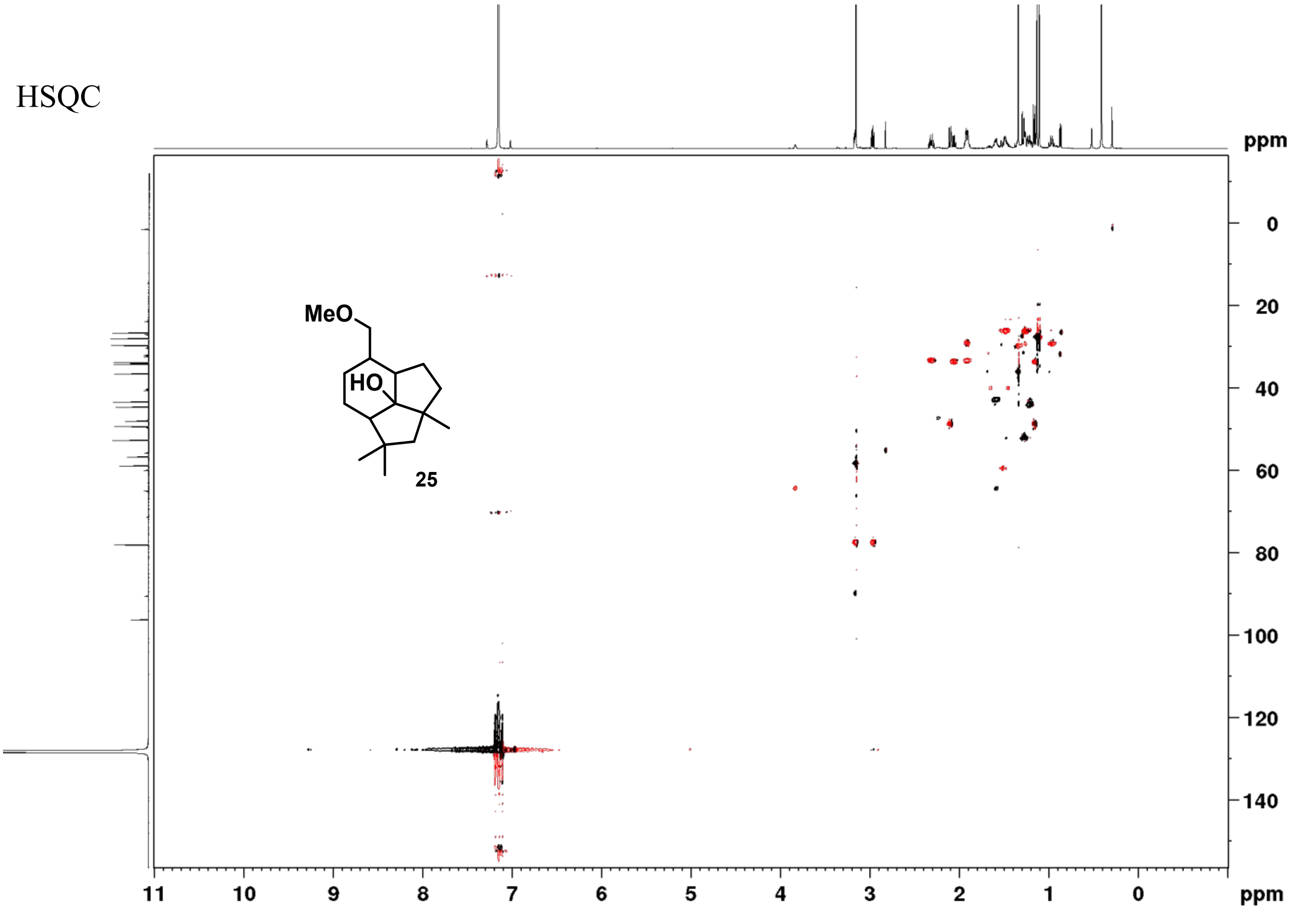
ppm
MeO
HO
25
0
20
40
60
80
100
120
140
11
10
9
8
7
6
5
4
3
2
1
0
ppm

HMBC

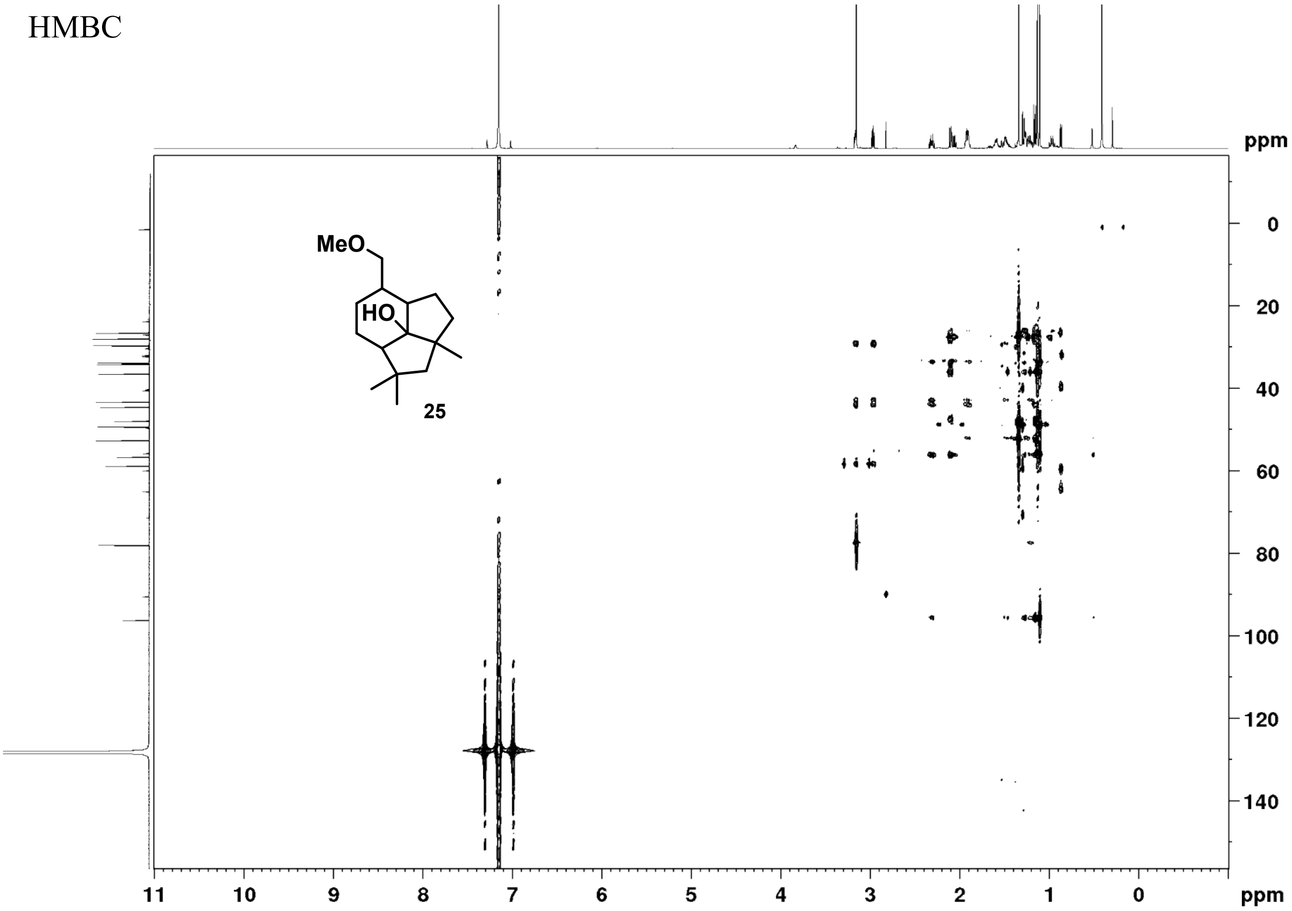
MeO
HO
25
ppm
0
20
40
60
80
100
120
140
11
10
9
8
7
6
5
4
3
2
1
0

COSY

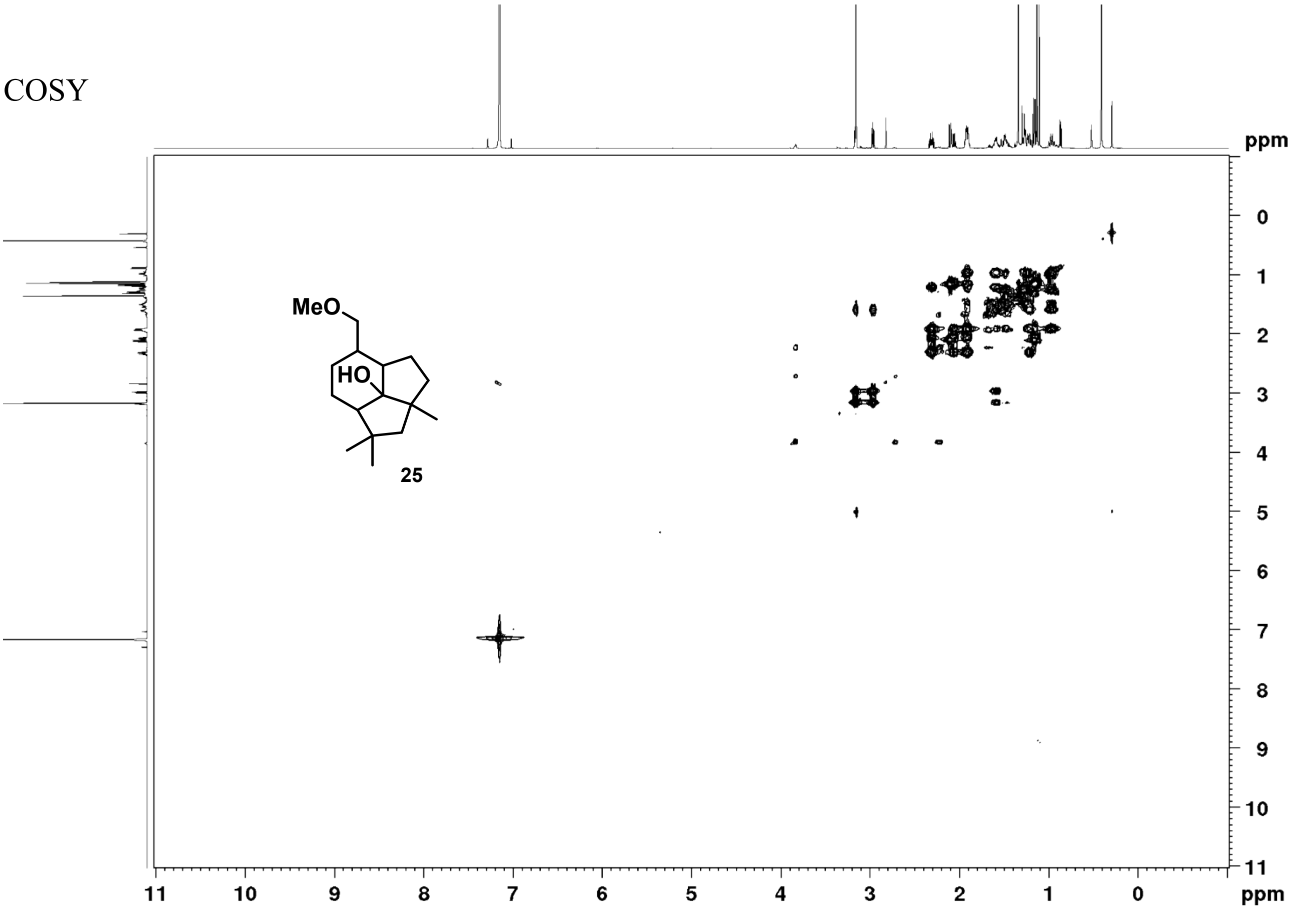
MeO
HO
25
ppm
0
1
2
3
4
5
6
7
8
9
10
11

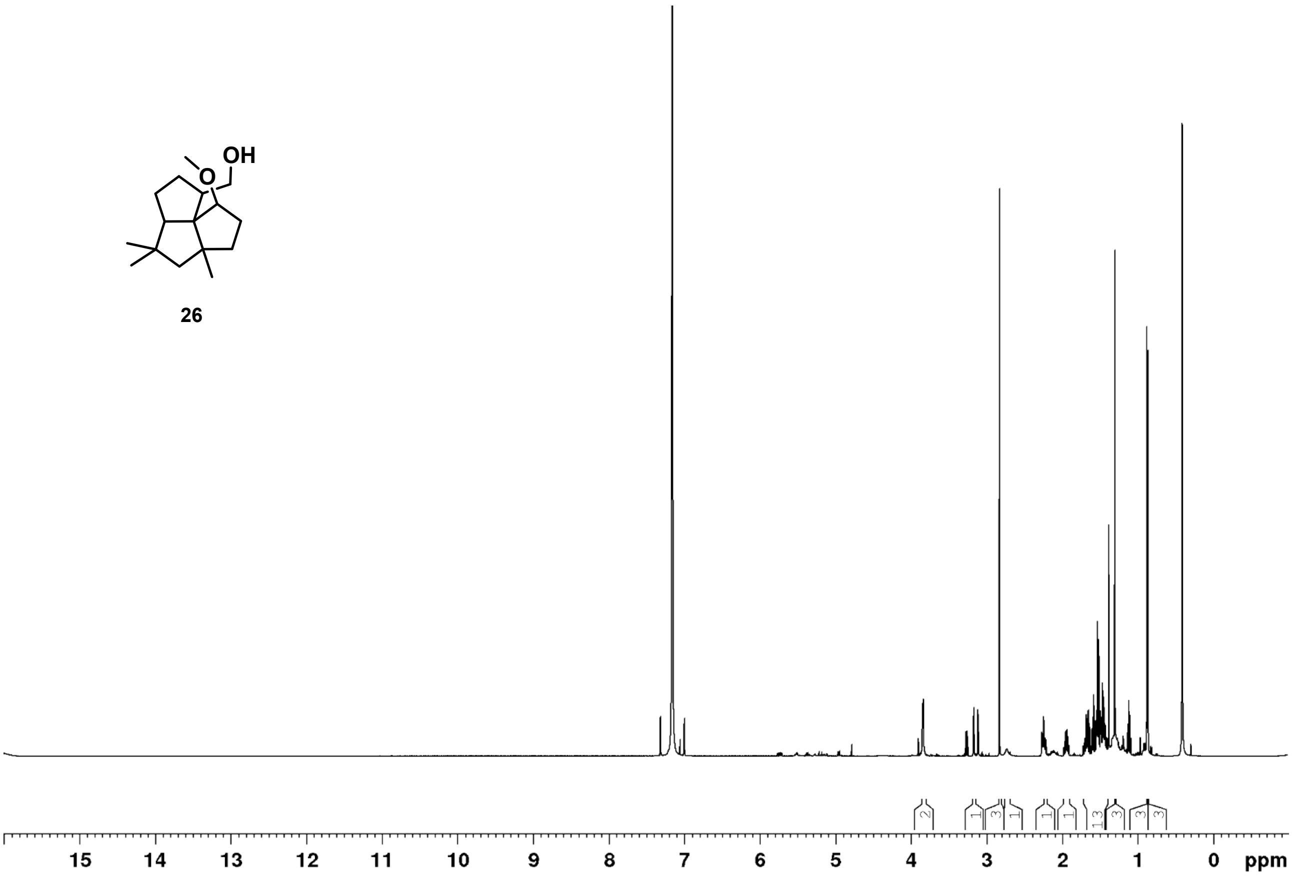
OH
O
26
2
1
3
1
1
1
13
3
3
3
15
14
13
12
11
10
9
8
7
6
5
4
3
2
1
0
ppm

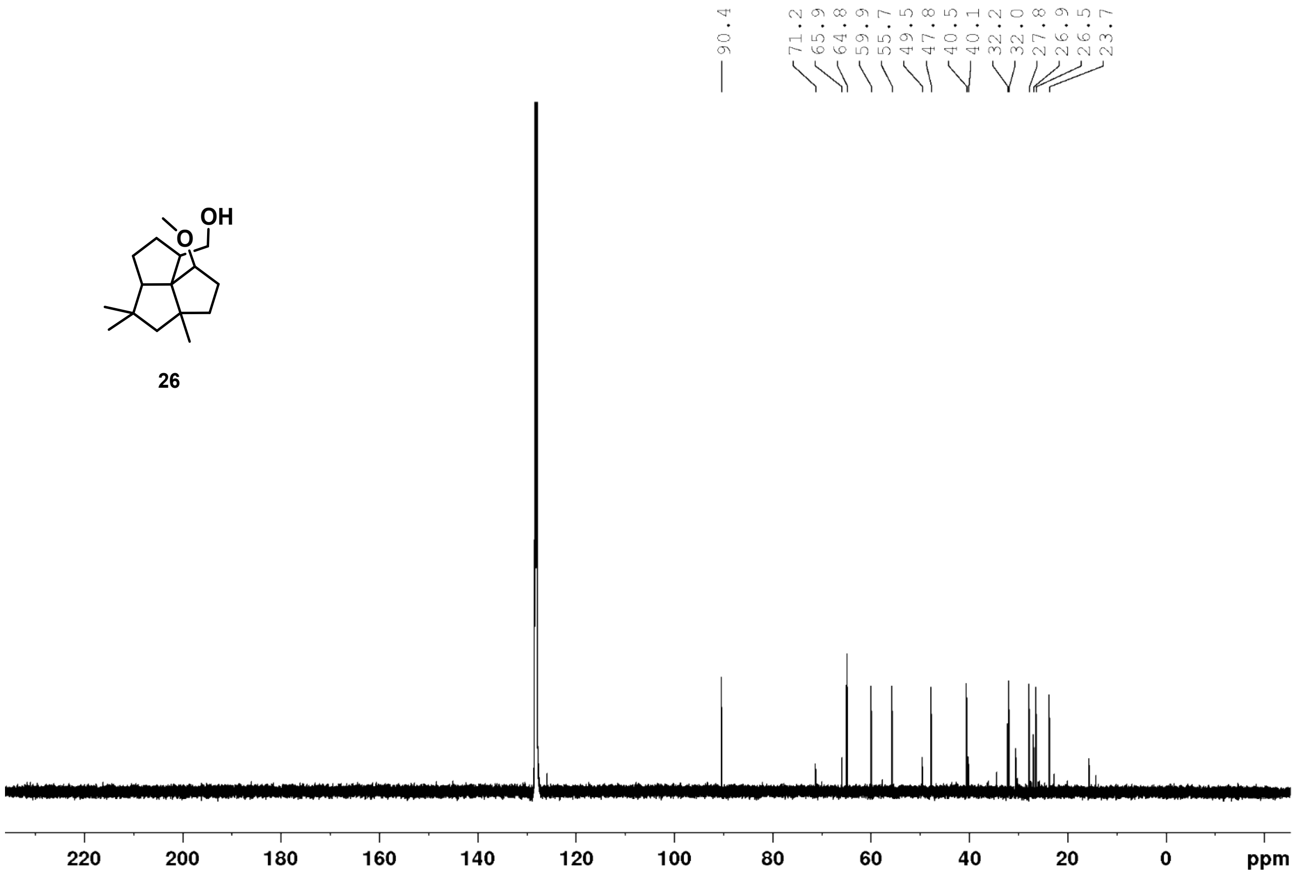
90.4
71.2
65.9
64.8
59.9
55.7
49.5
47.8
40.5
40.1
32.2
32.0
27.8
26.9
26.5
23.7
OH
O
26
220
200
180
160
140
120
100
80
60
40
20
0
ppm

HSQC

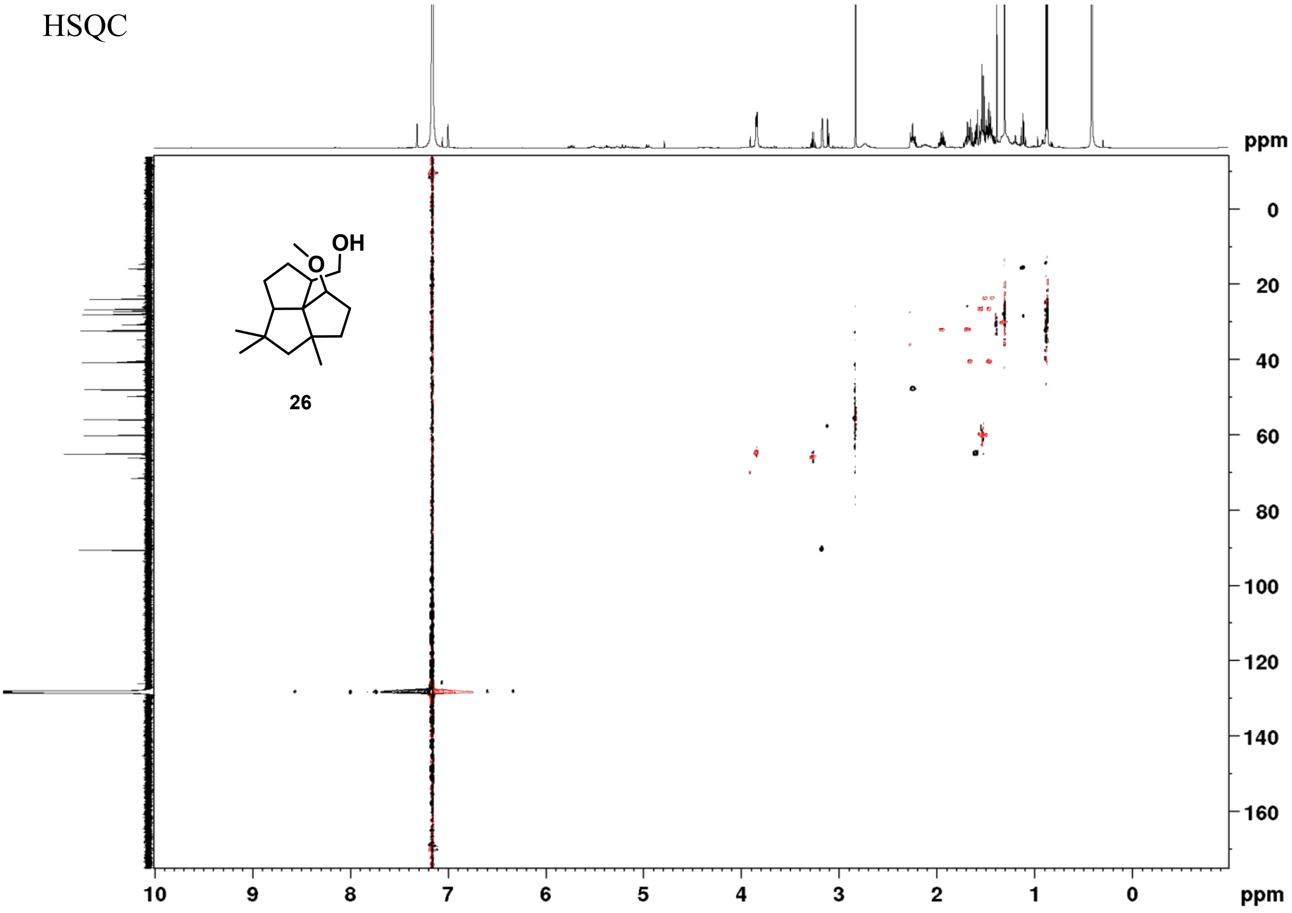
OH
O
26
ppm
0
20
40
60
80
100
120
140
160
10
9
8
7
6
5
4
3
2
1
0
ppm

HMBC

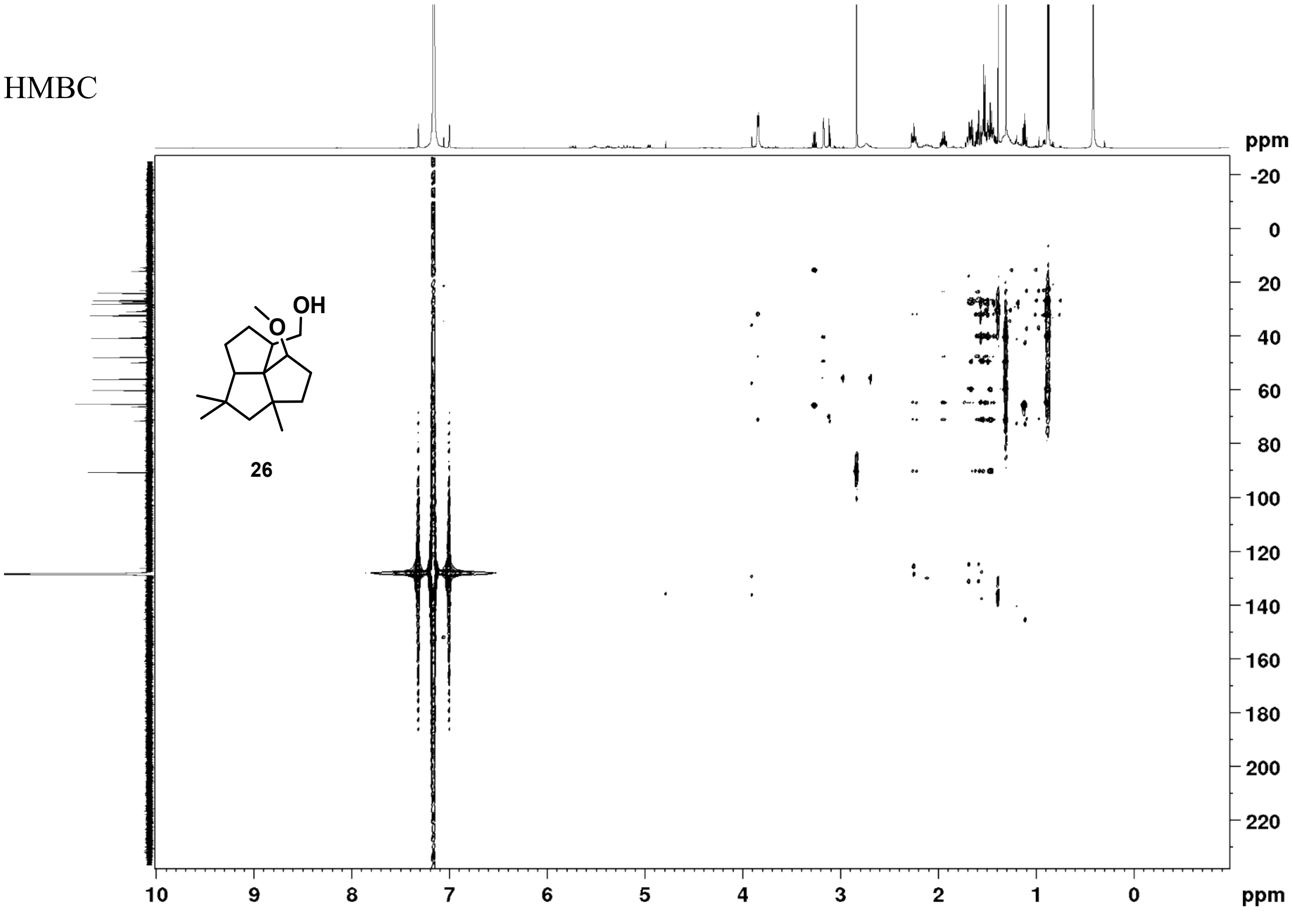
OH
O
26
ppm
-20
0
20
40
60
80
100
120
140
160
180
200
220
10
9
8
7
6
5
4
3
2
1
0
ppm

COSY

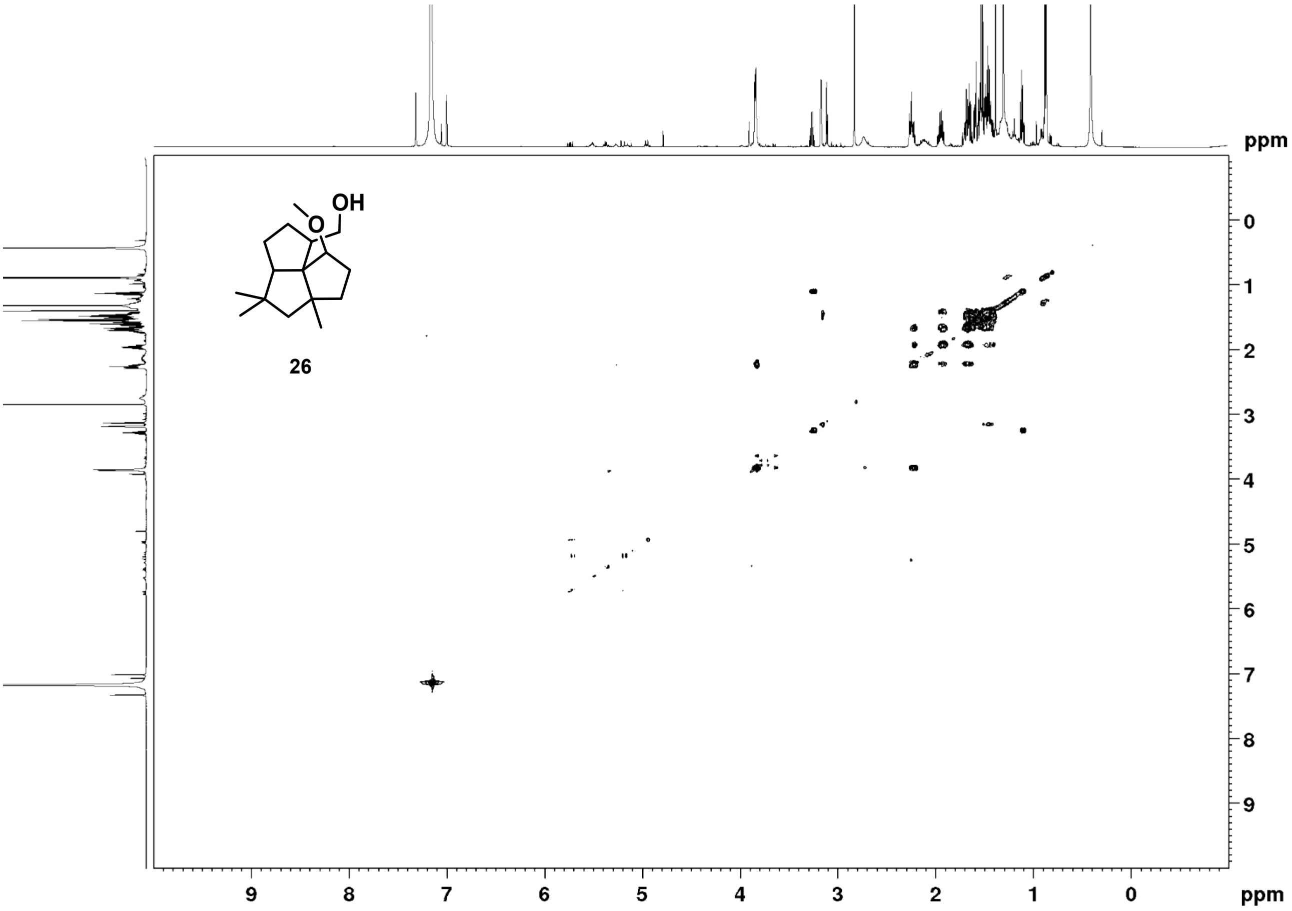

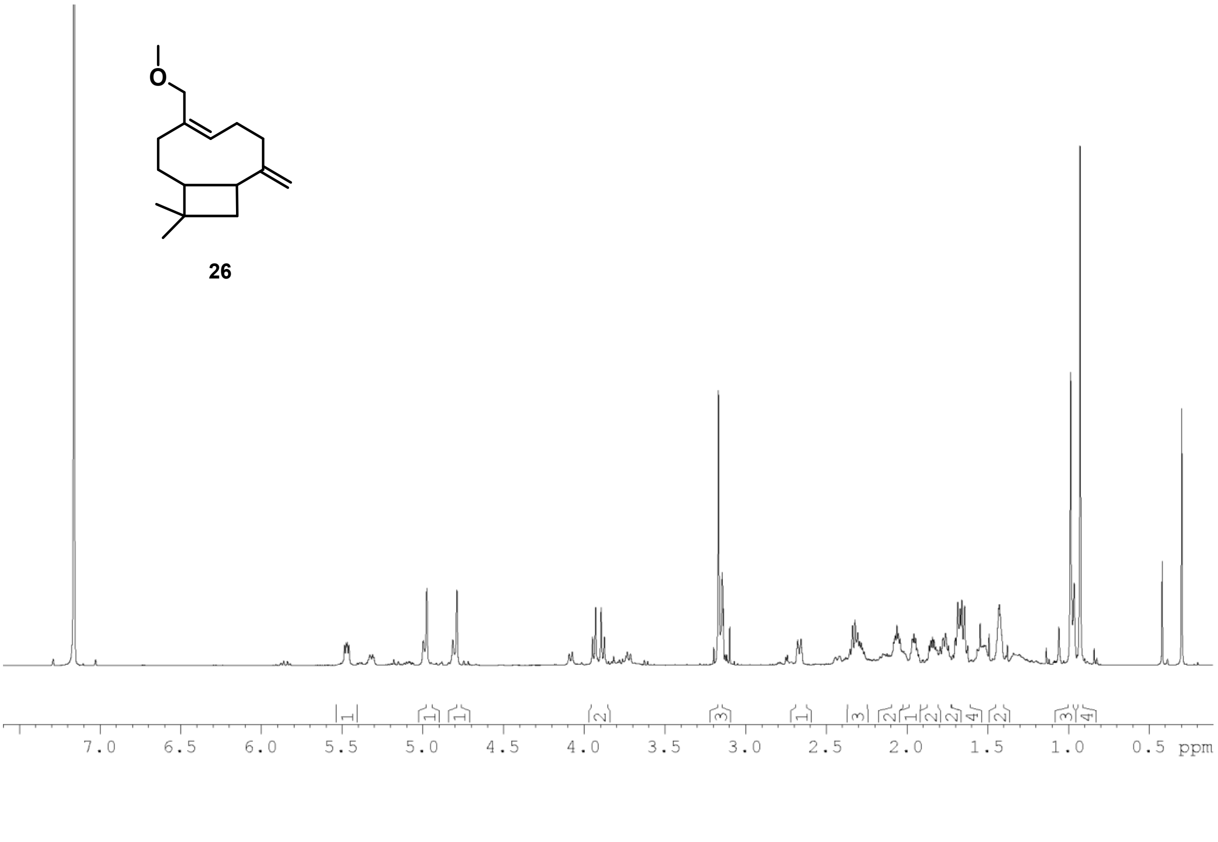

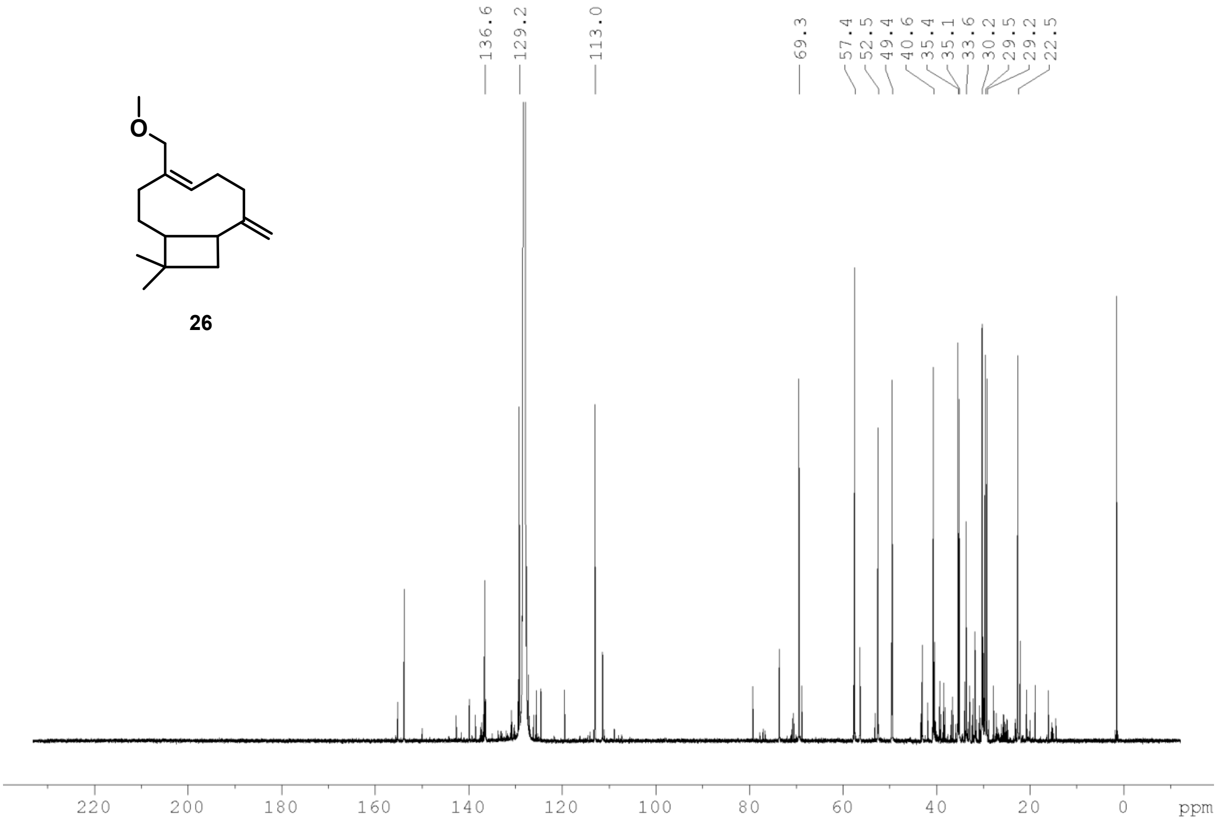
136.6
129.2
113.0
69.3
57.4
52.5
49.4
40.6
35.4
35.1
33.6
30.2
29.5
29.2
22.5
O
26
220
200
180
160
140
120
100
80
60
40
20
0
ppm

HSQC

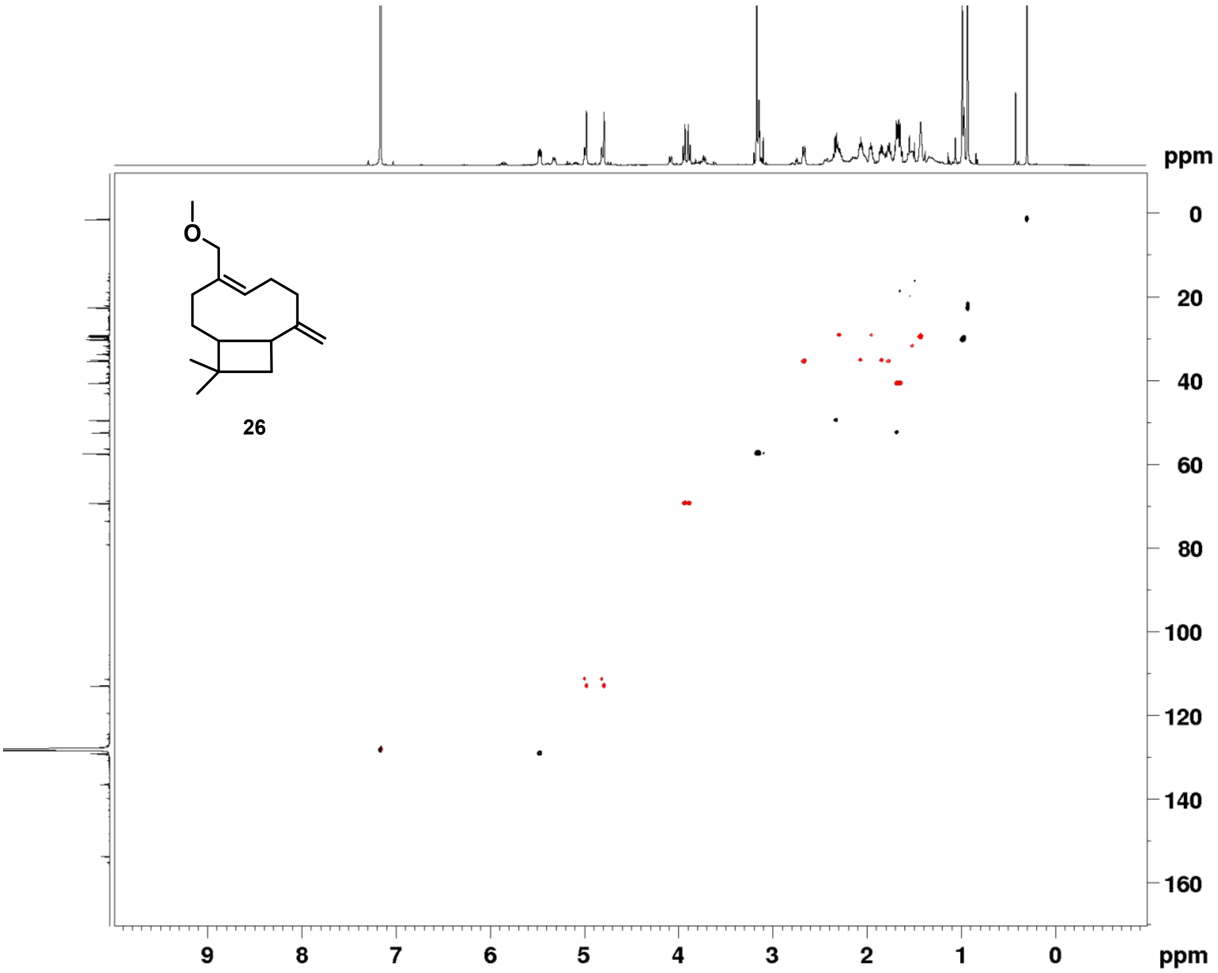
O
26
ppm
0
20
40
60
80
100
120
140
160
9
8
7
6
5
4
3
2
1
0
ppm

HMBC

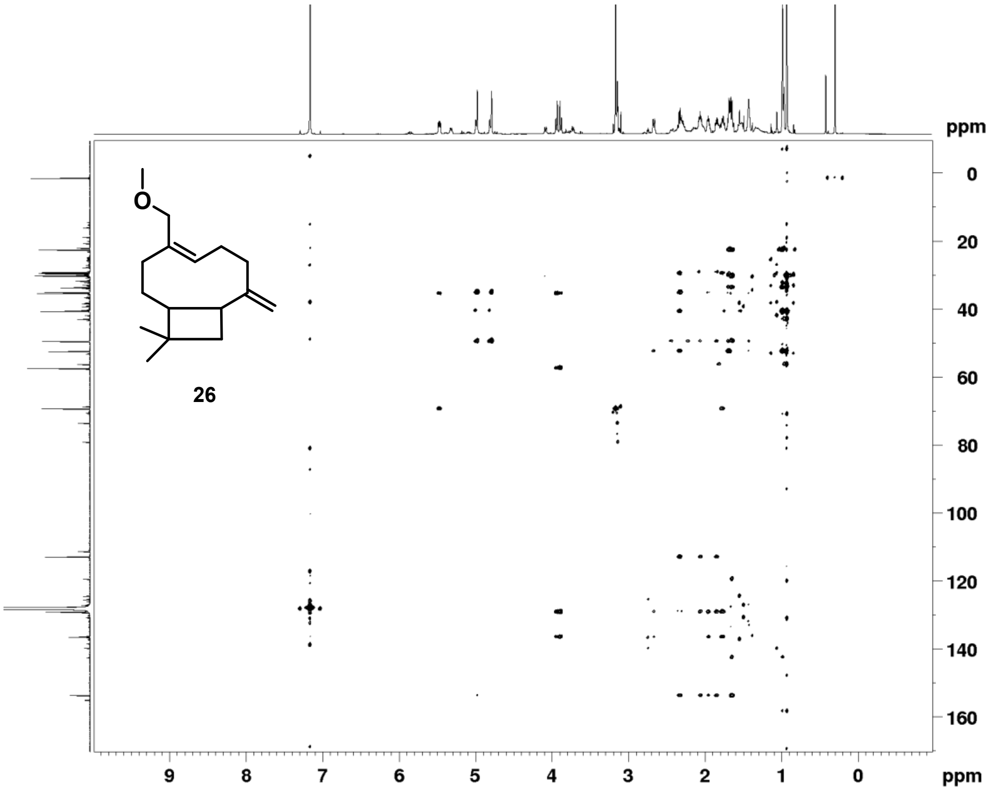
O
26
ppm
0
20
40
60
80
100
120
140
160
9
8
7
6
5
4
3
2
1
0
ppm

COSY

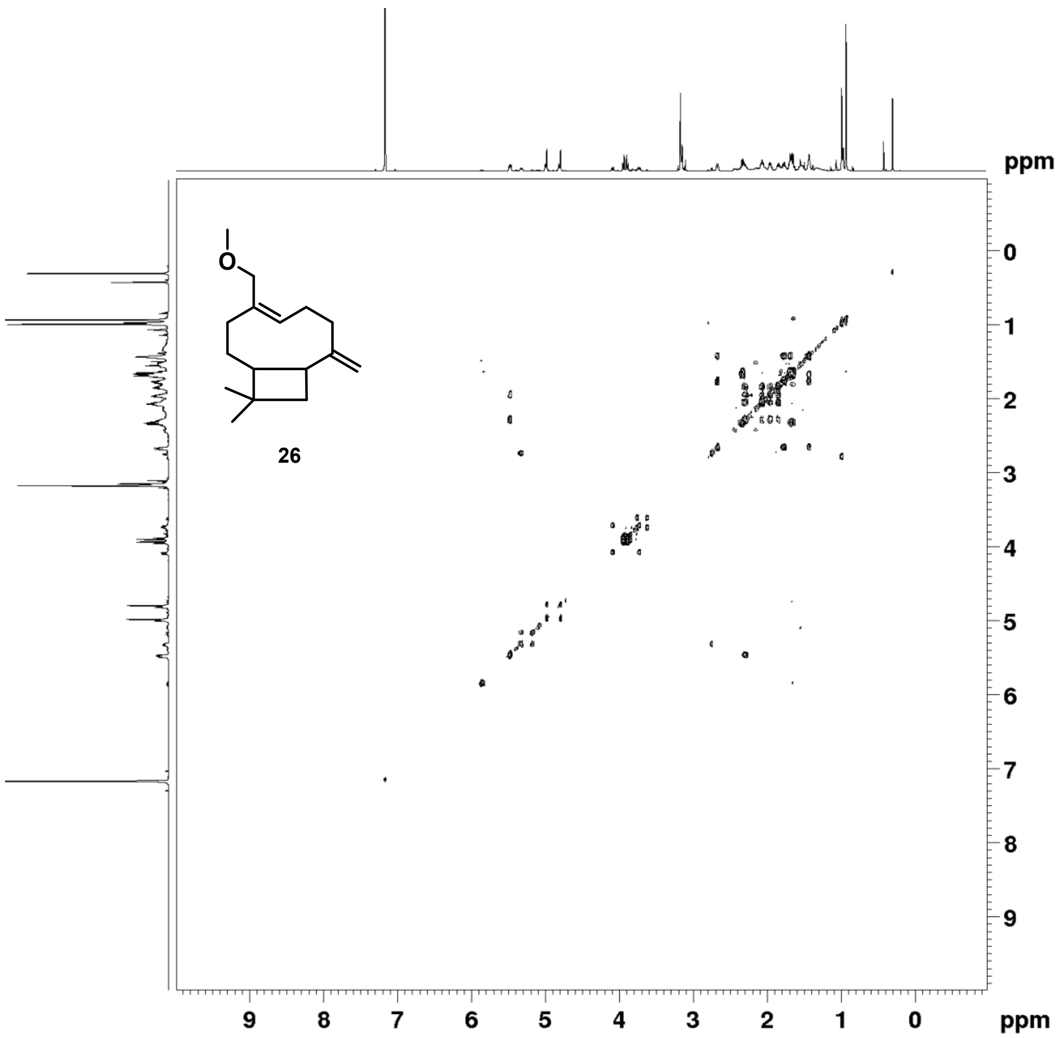
26
ppm
ppm

^{1}H

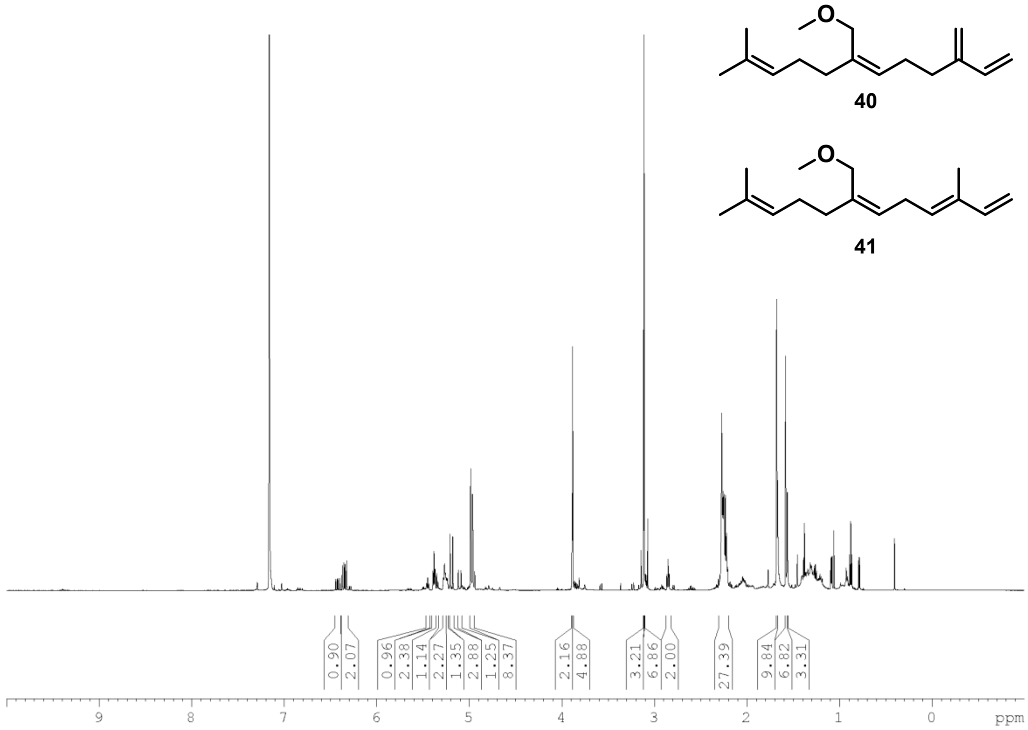
40
41
0.90
2.07
0.96
2.38
1.14
2.27
1.35
2.88
1.25
8.37
2.16
4.88
3.21
6.86
2.00
27.39
9.84
6.82
3.31
ppm

^{13}C

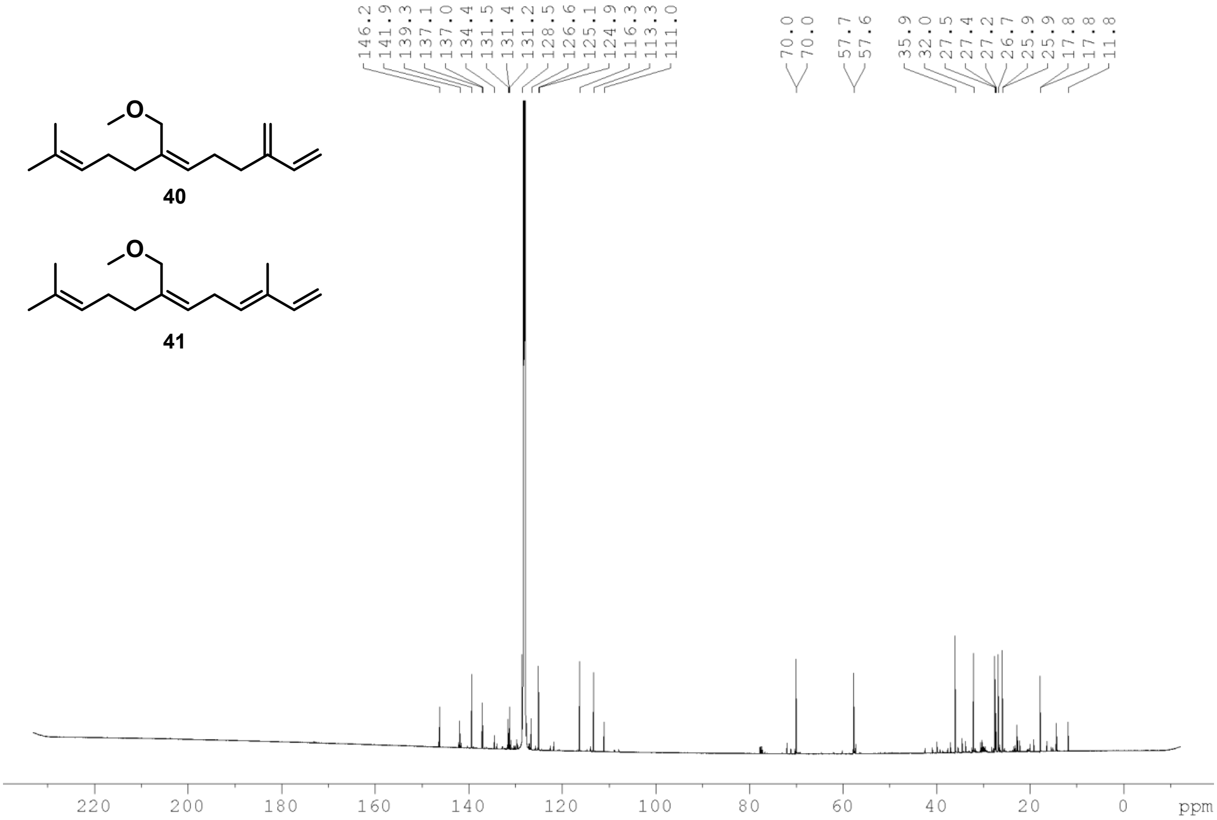

146.2
141.9
139.3
137.1
137.0
134.4
131.5
131.4
131.2
128.5
126.6
125.1
124.9
116.3
113.3
111.0
70.0
70.0
57.7
57.6
35.9
32.0
27.5
27.4
27.2
26.7
25.9
25.9
17.8
17.8
11.8
O
40
O
41
220 200 180 160 140 120 100 80 60 40 20 0 ppm

HSQC

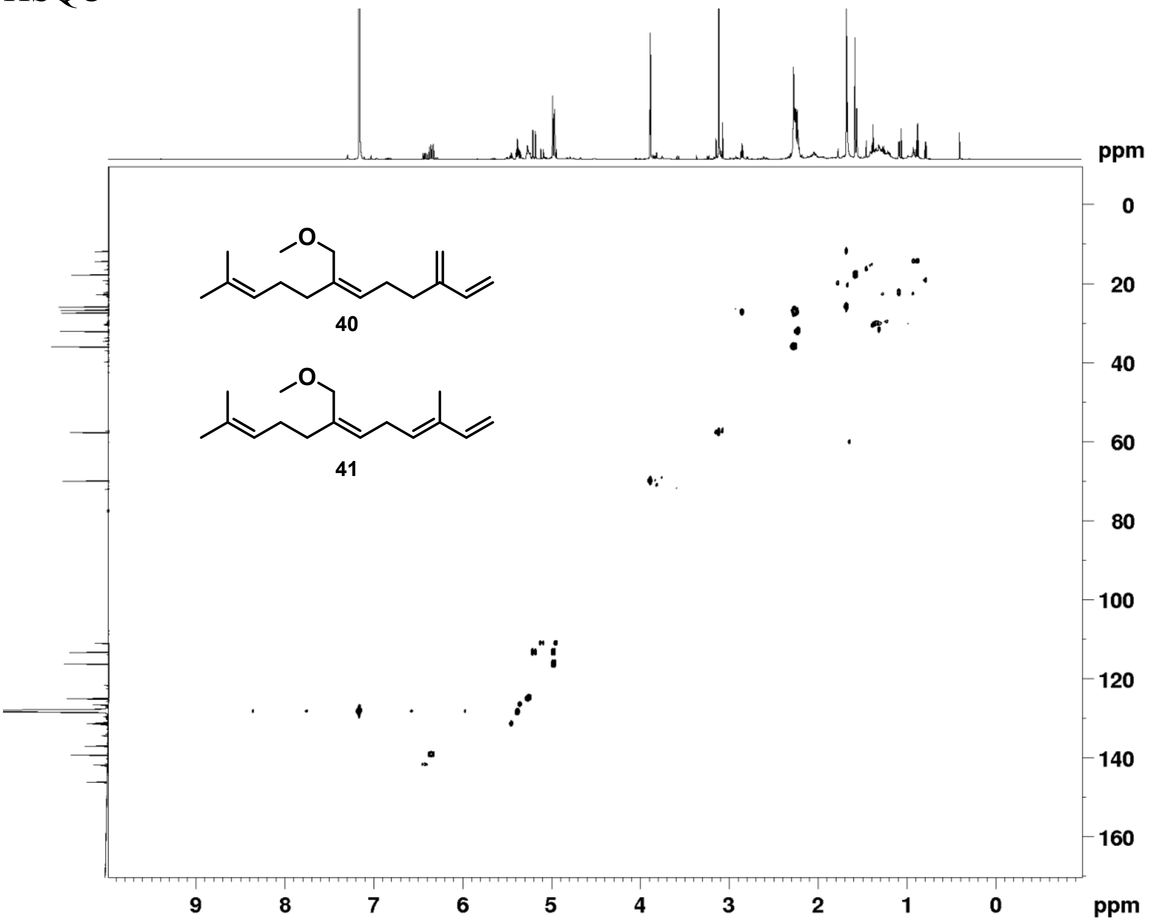

O
40
O
41
ppm
0 20 40 60 80 100 120 140 160
9 8 7 6 5 4 3 2 1 0
ppm

HMBC

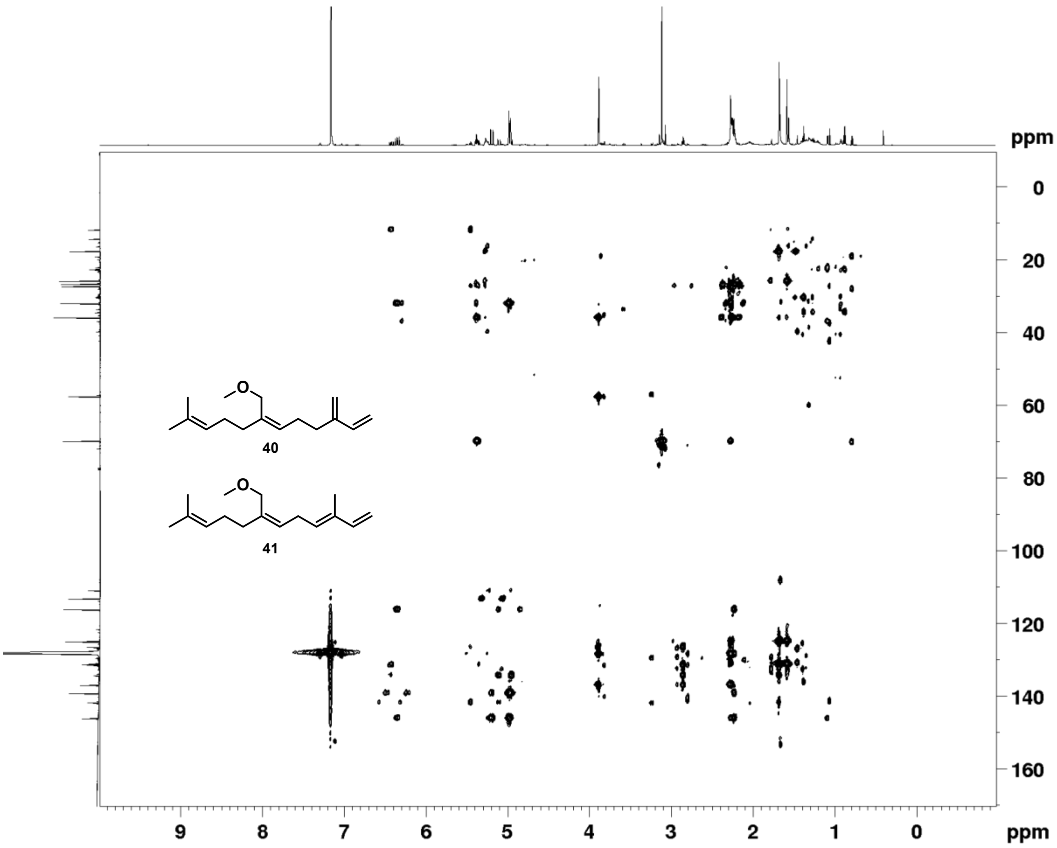

40
41
ppm
0
20
40
60
80
100
120
140
160
9
8
7
6
5
4
3
2
1
0
ppm

COSY

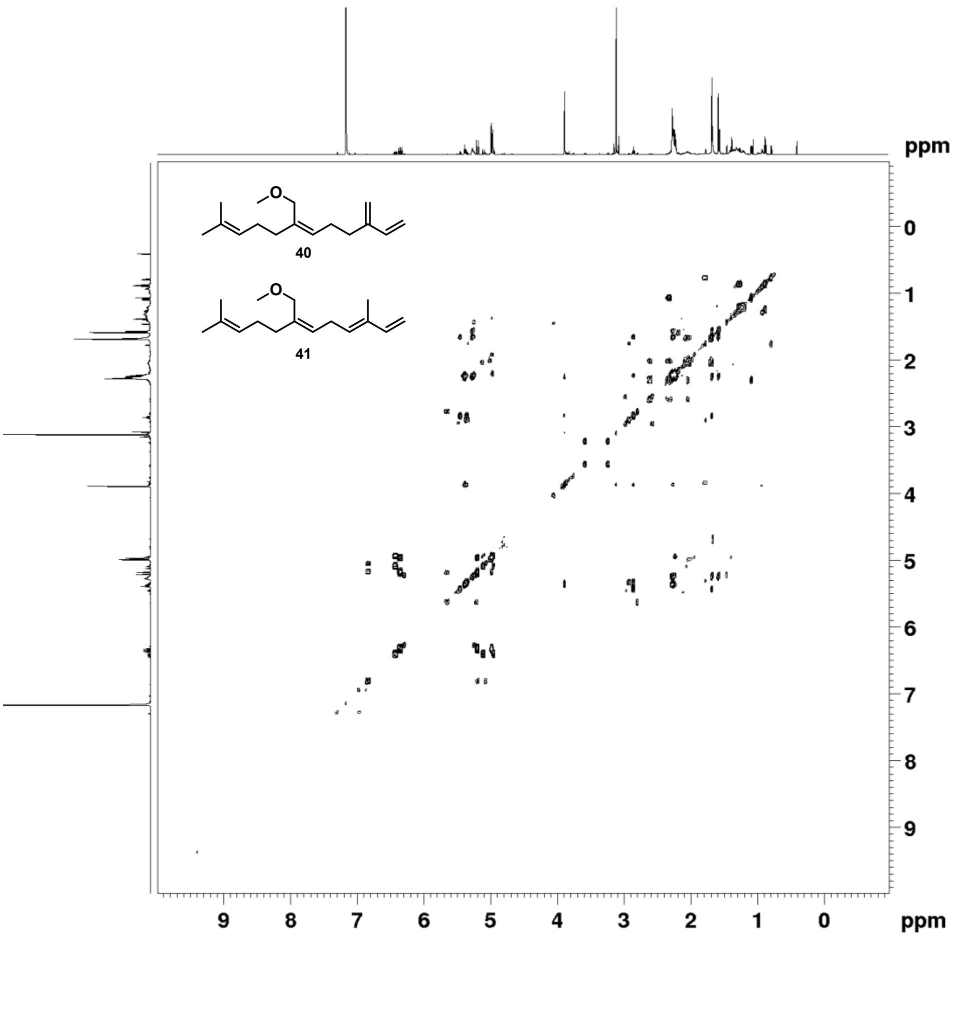

40
41
ppm
0
1
2
3
4
5
6
7
8
9
9
8
7
6
5
4
3
2
1
0
ppm

C. Copies of ^{1}H- and ^{13}C-NMR spectra (chemical synthesis)

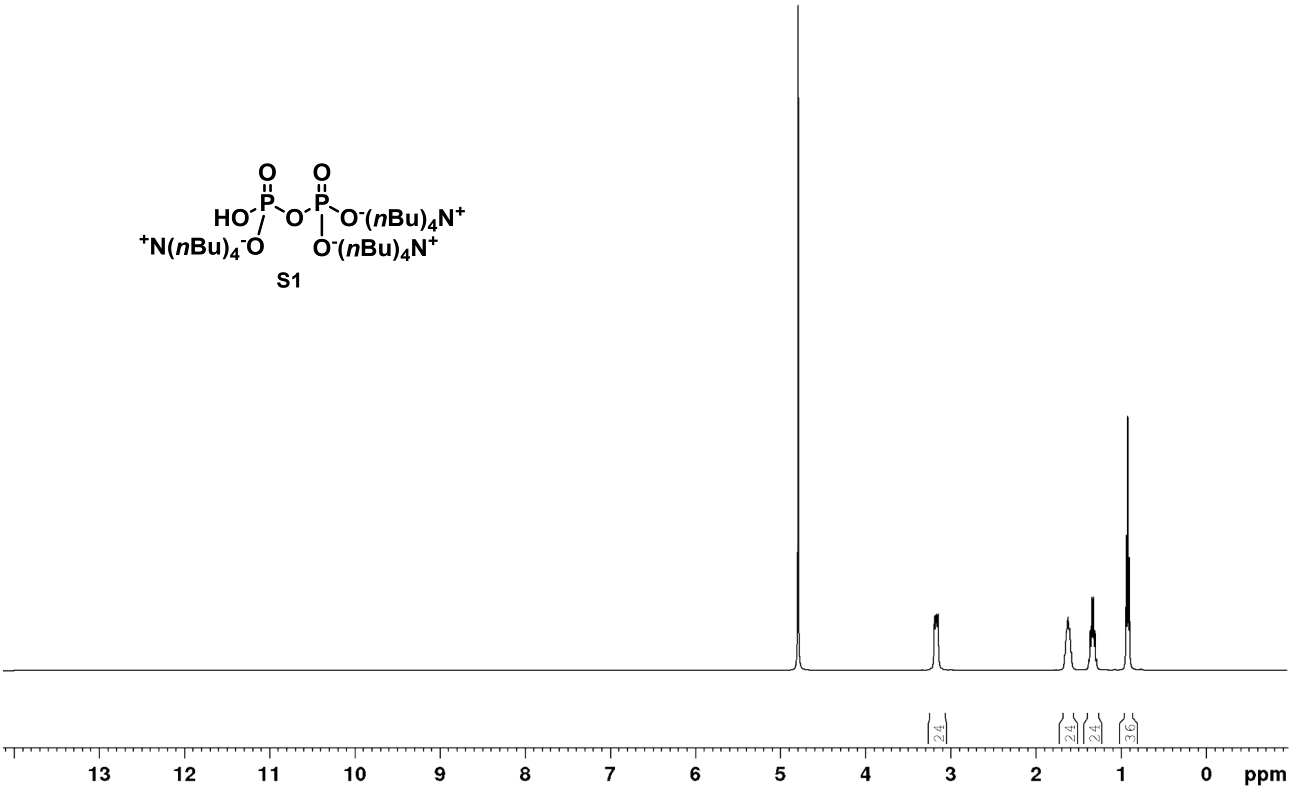

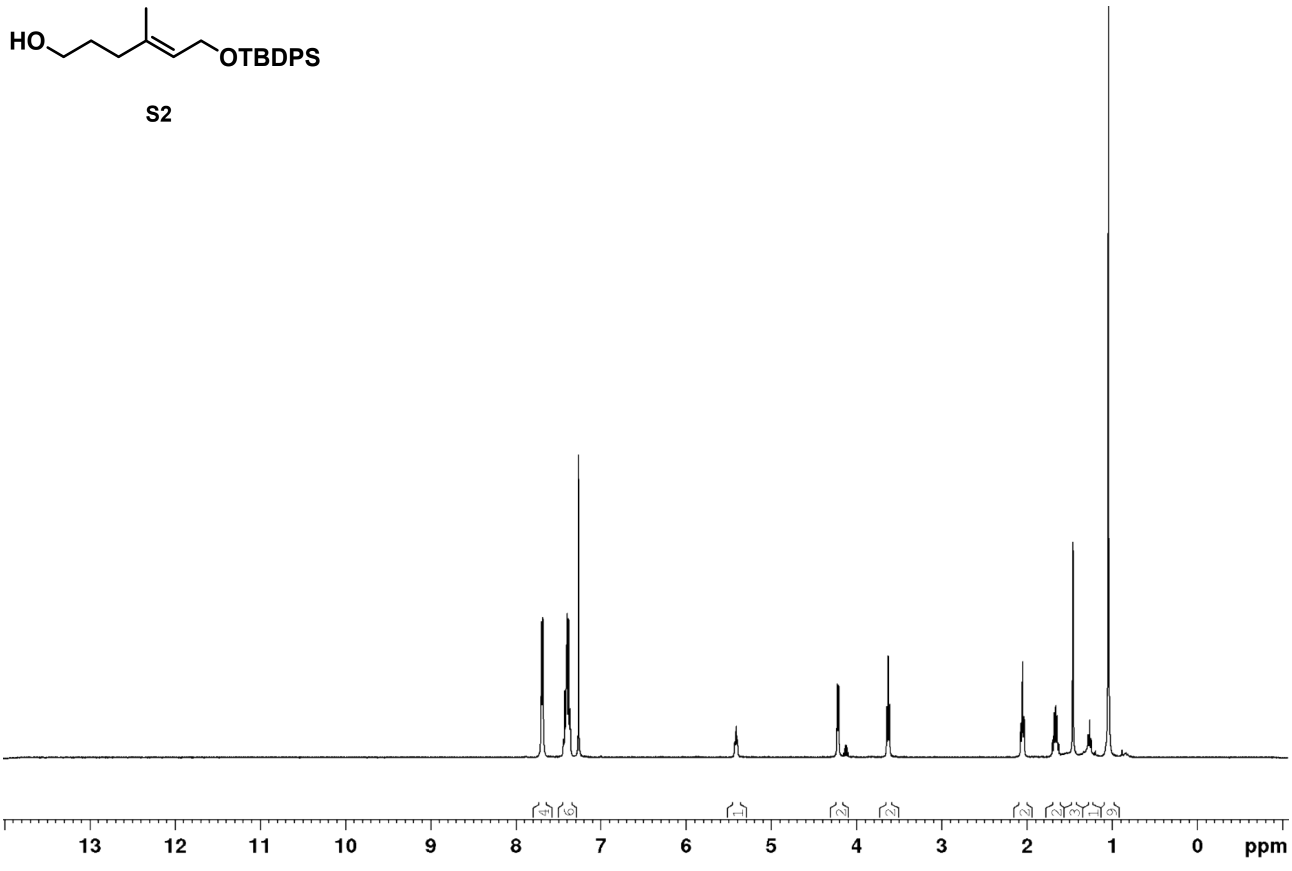

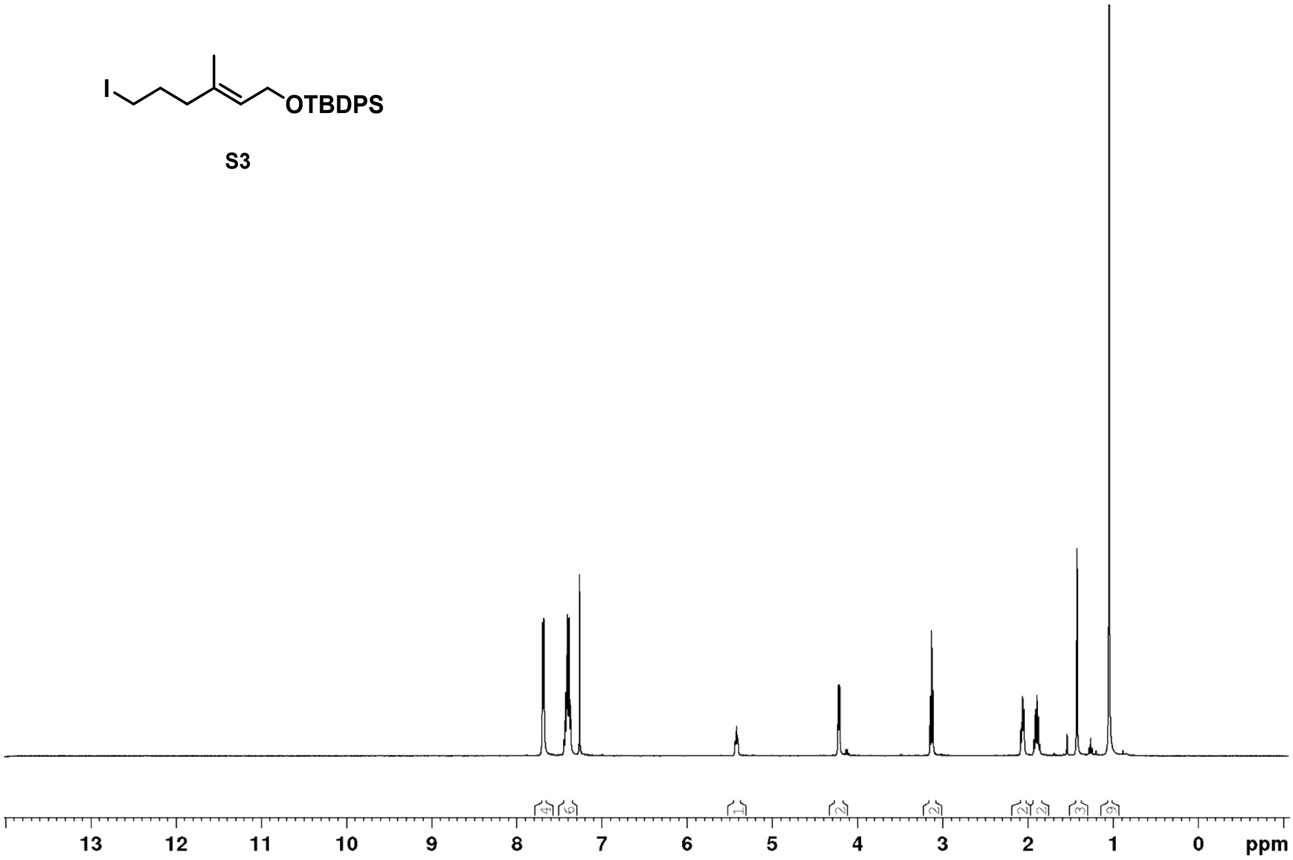
I
OTBDPS
13 12 11 10 9 8 7 6 5 4 3 2 1 0 ppm

S3

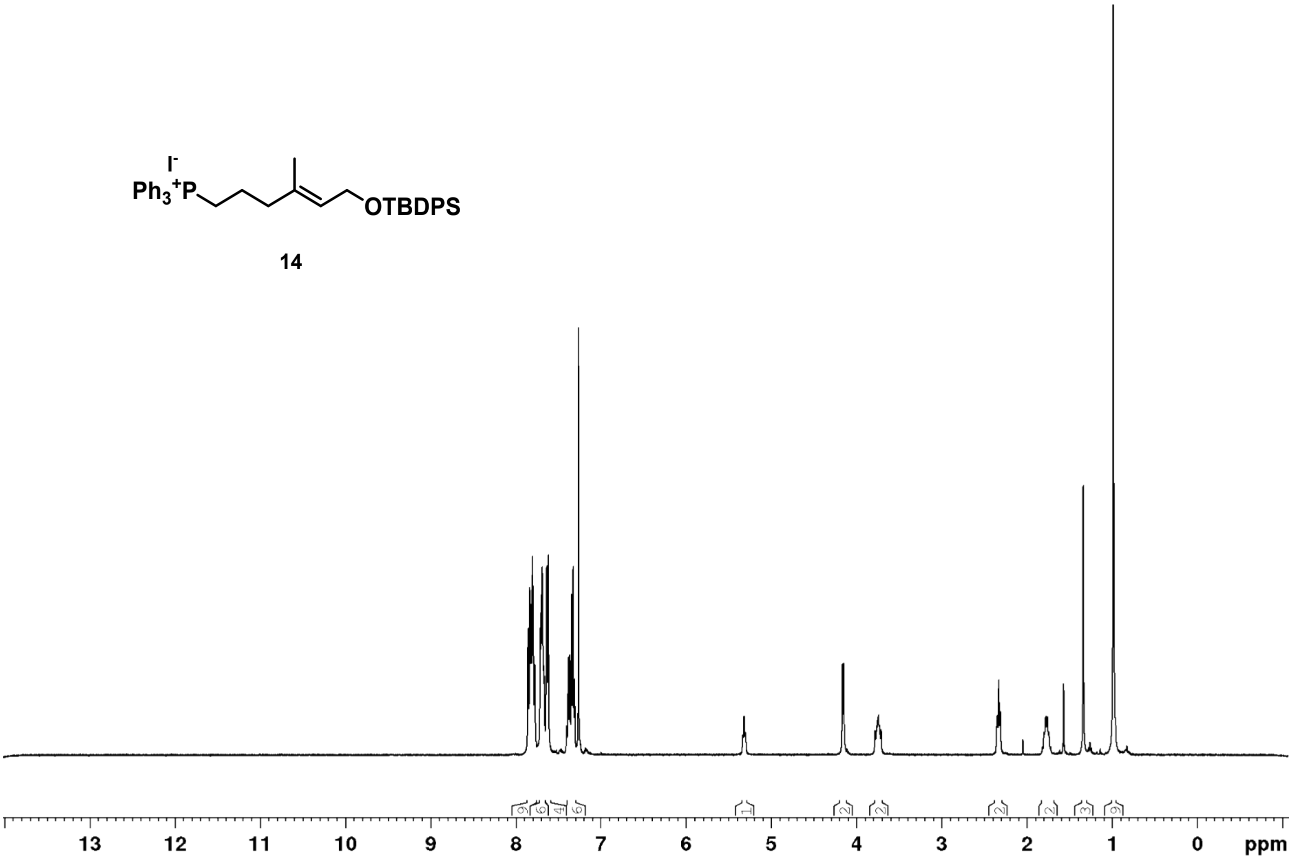
I-
Ph3+P
OTBDPS
13 12 11 10 9 8 7 6 5 4 3 2 1 0 ppm

14

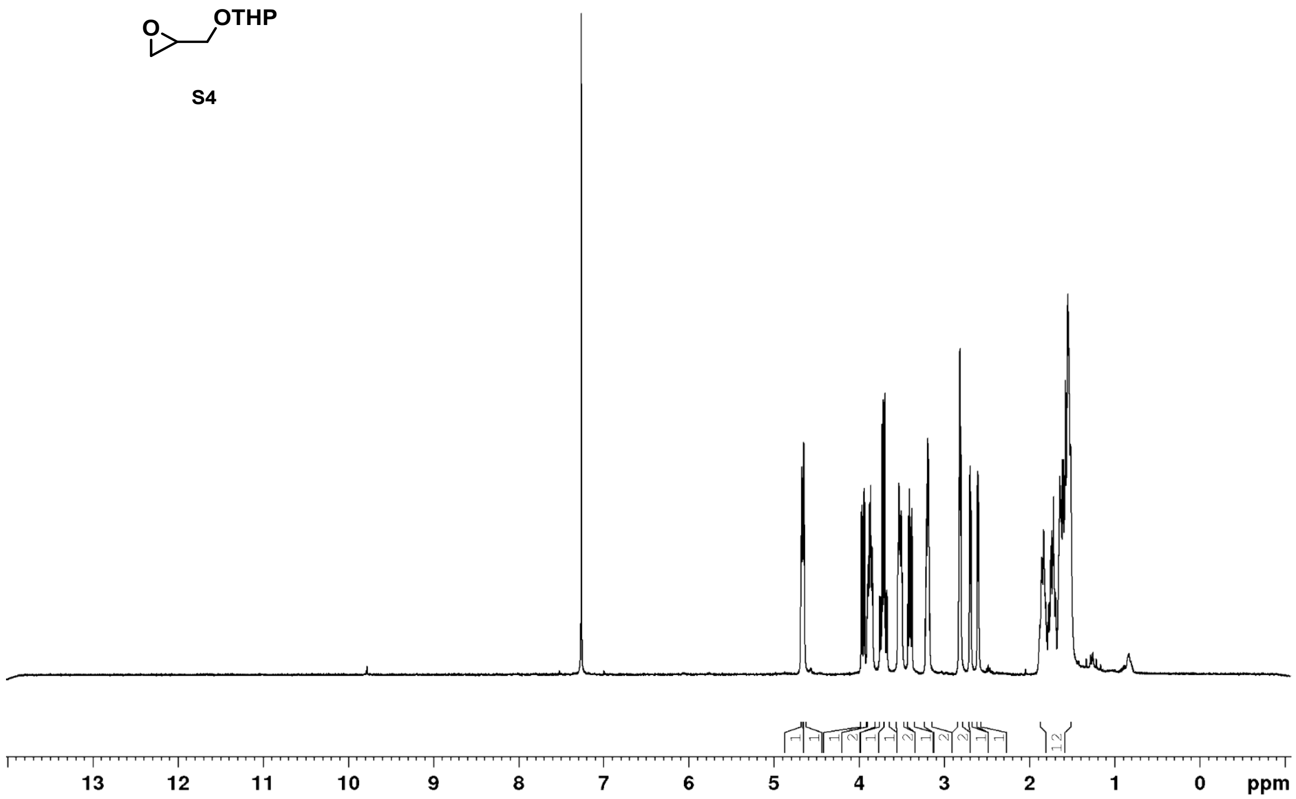
OTHP
S4
ppm

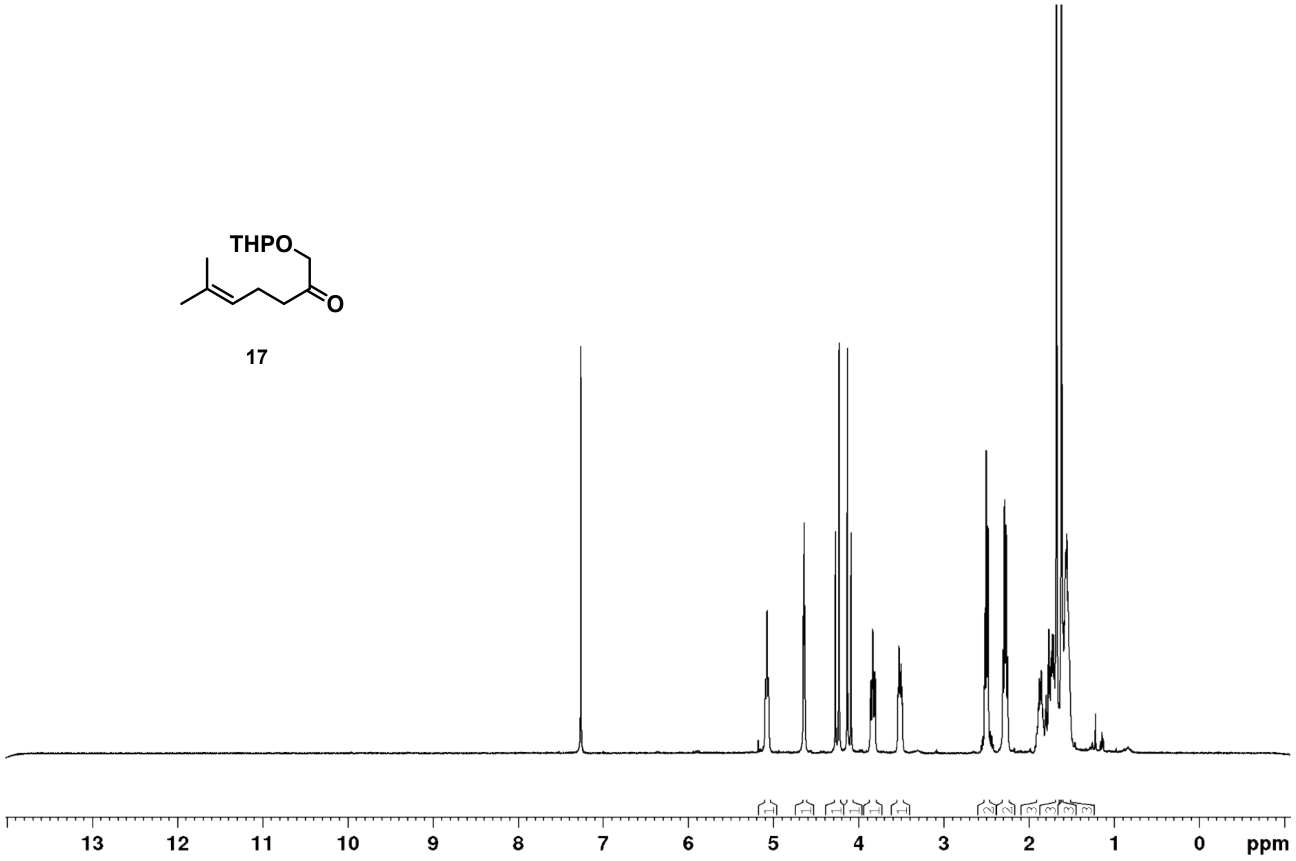
THPO
O
17
ppm

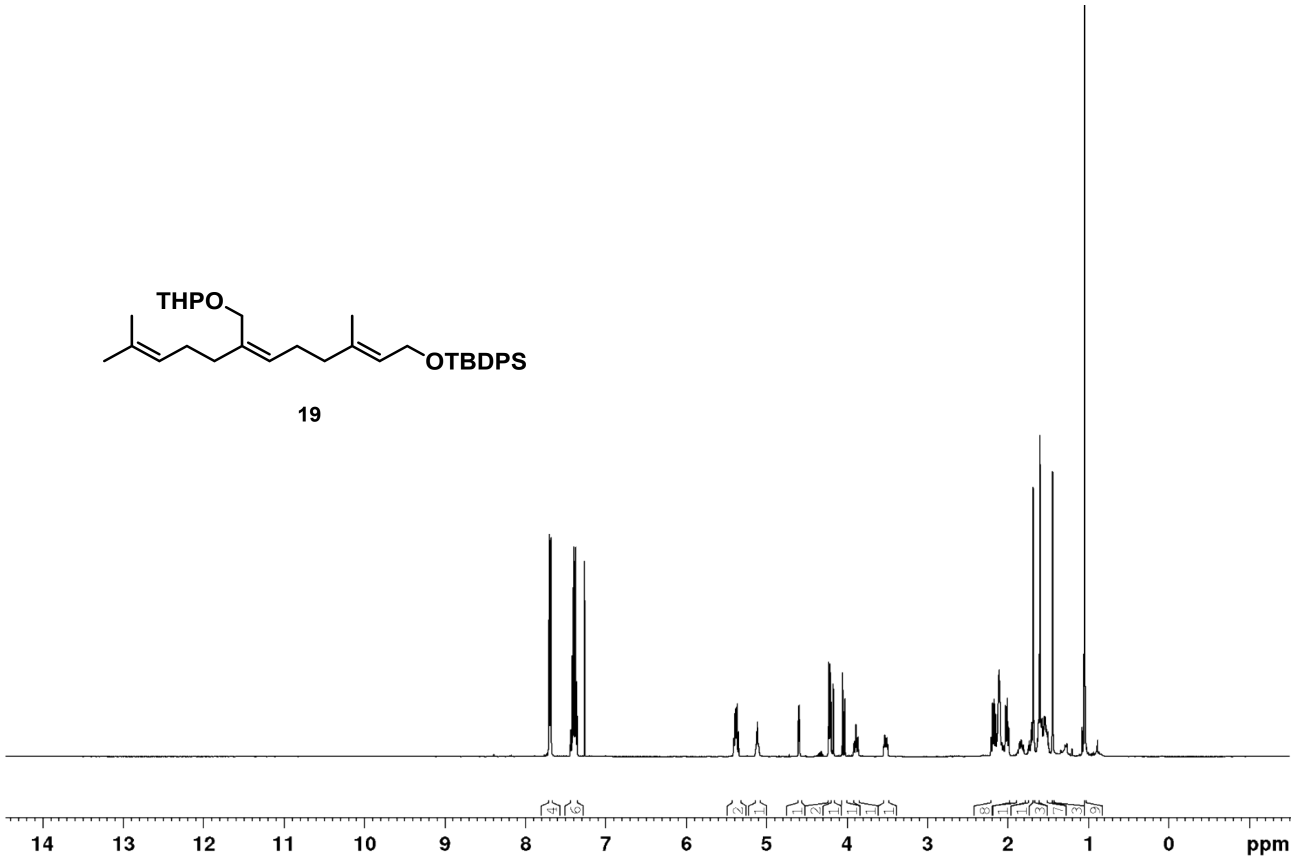
THPO
OTBDPS
19
4 6 2 1 1 2 1 1 1 8 1 1 3 7 3 9
14 13 12 11 10 9 8 7 6 5 4 3 2 1 0 ppm

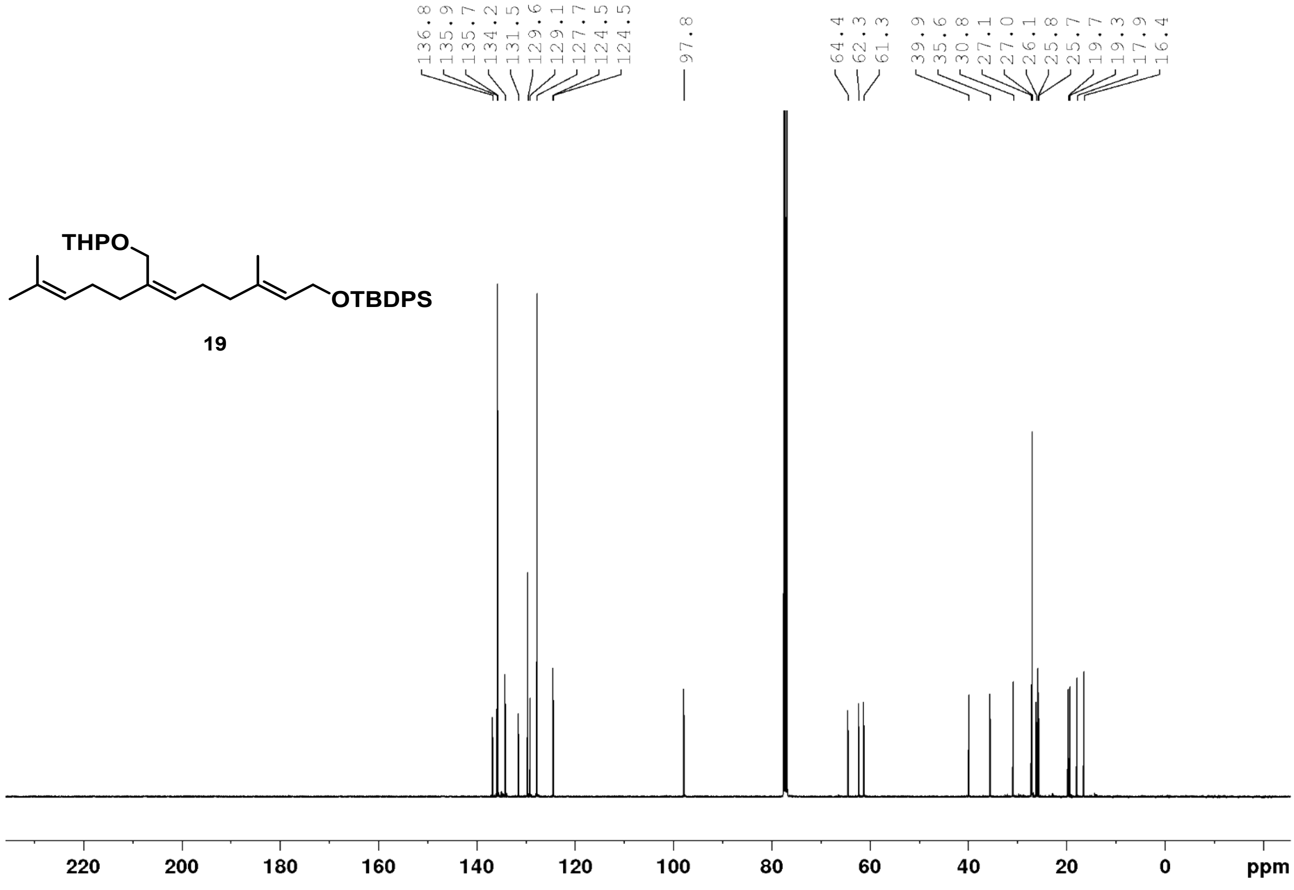
136.8 135.9 135.7 134.2 131.5 129.6 129.1 127.7 124.5 124.5
97.8
64.4 62.3 61.3
39.9 35.6 30.8 27.1 27.0 26.1 25.8 25.7 19.7 19.3 17.9 16.4
THPO
OTBDPS
19
220 200 180 160 140 120 100 80 60 40 20 0 ppm

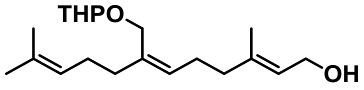
THPO
OH

S6

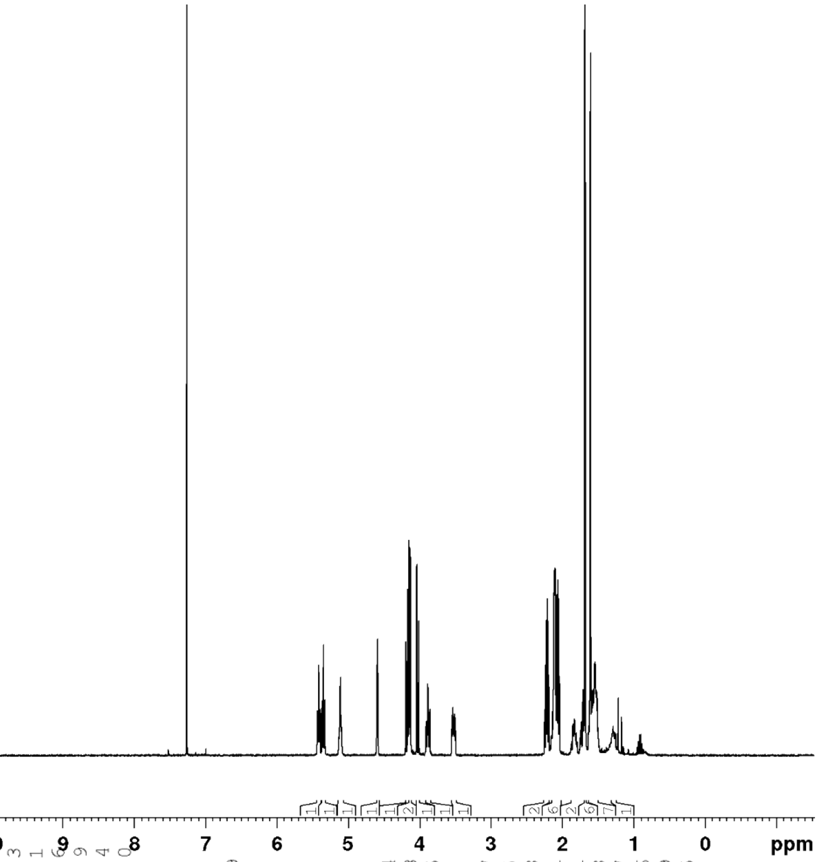
16 15 14 13 12 11 10 9 8 7 6 5 4 3 2 1 0 ppm

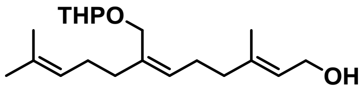
THPO
OH

S6

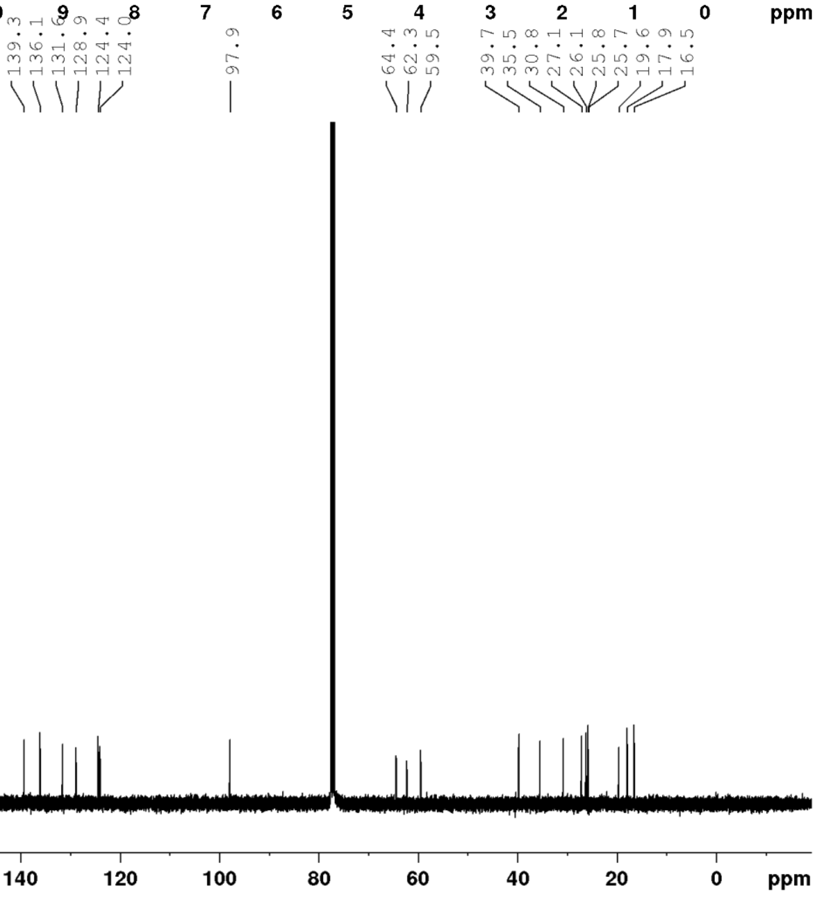
139.3
136.1
131.6
128.9
124.4
124.0
97.9
64.4
62.3
59.5
39.7
35.5
30.8
27.1
26.1
25.8
25.7
19.6
17.9
16.5
220 200 180 160 140 120 100 80 60 40 20 0 ppm

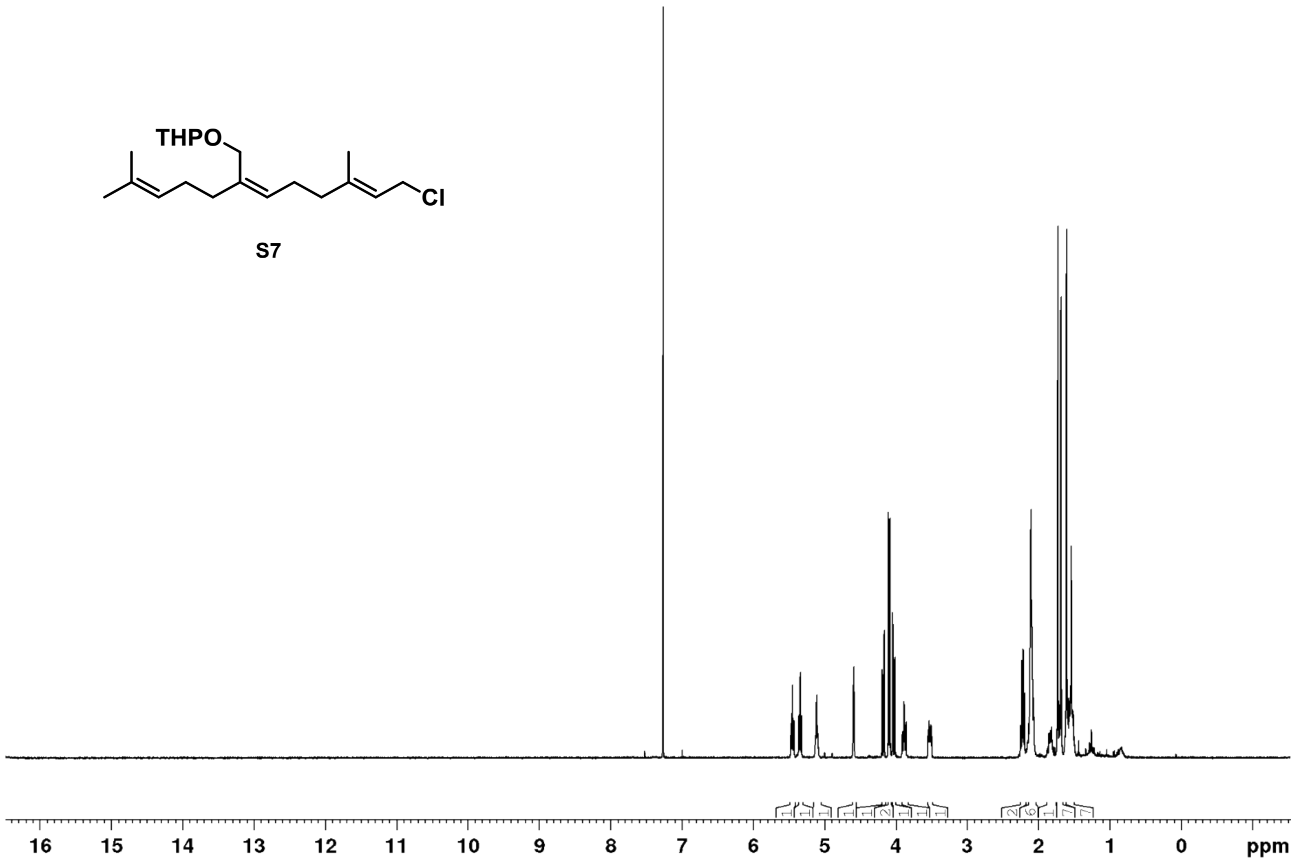
THPO
Cl
S7
16
15
14
13
12
11
10
9
8
7
6
5
4
3
2
1
0
ppm

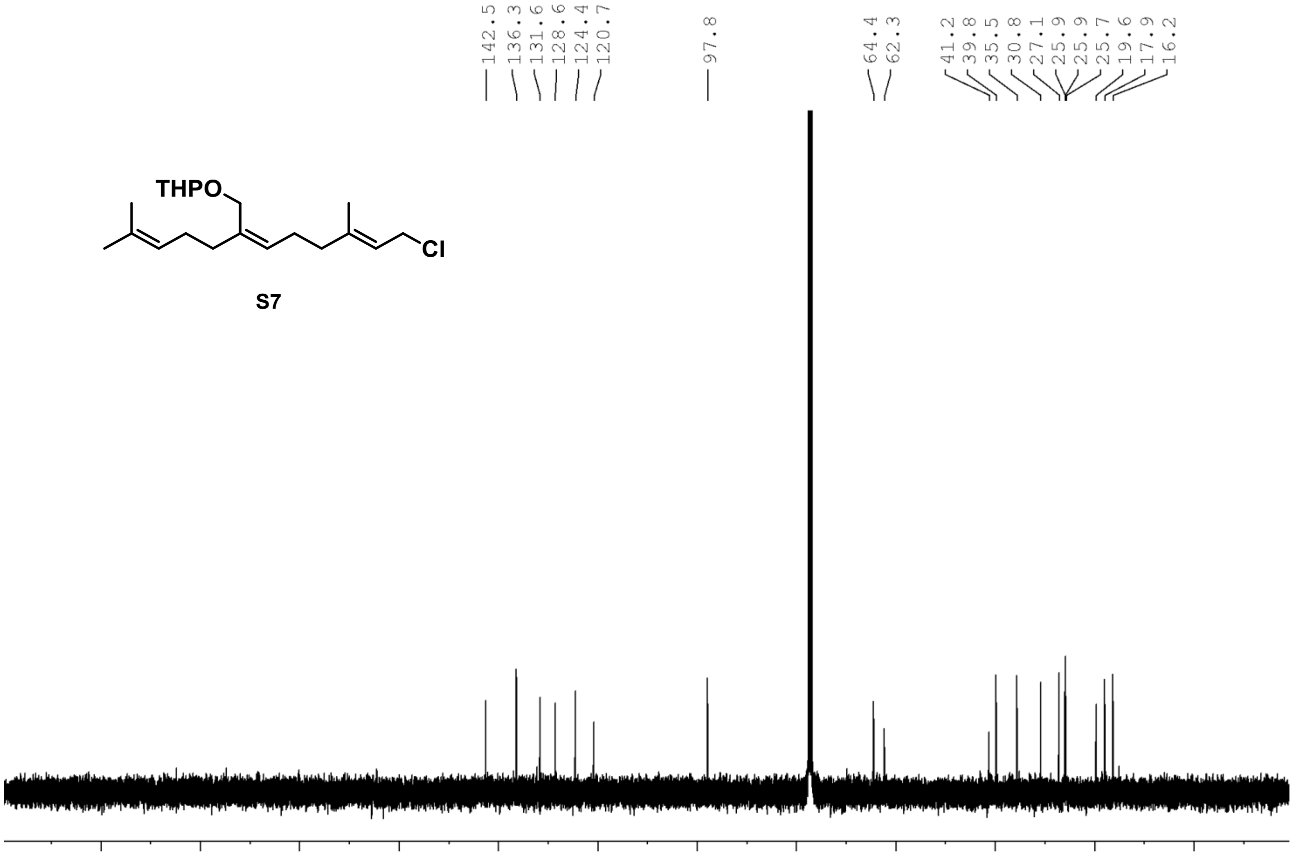
142.5
136.3
131.6
128.6
124.4
120.7
97.8
64.4
62.3
41.2
39.8
35.5
30.8
27.1
25.9
25.9
25.7
19.6
17.9
16.2
THPO
Cl
S7

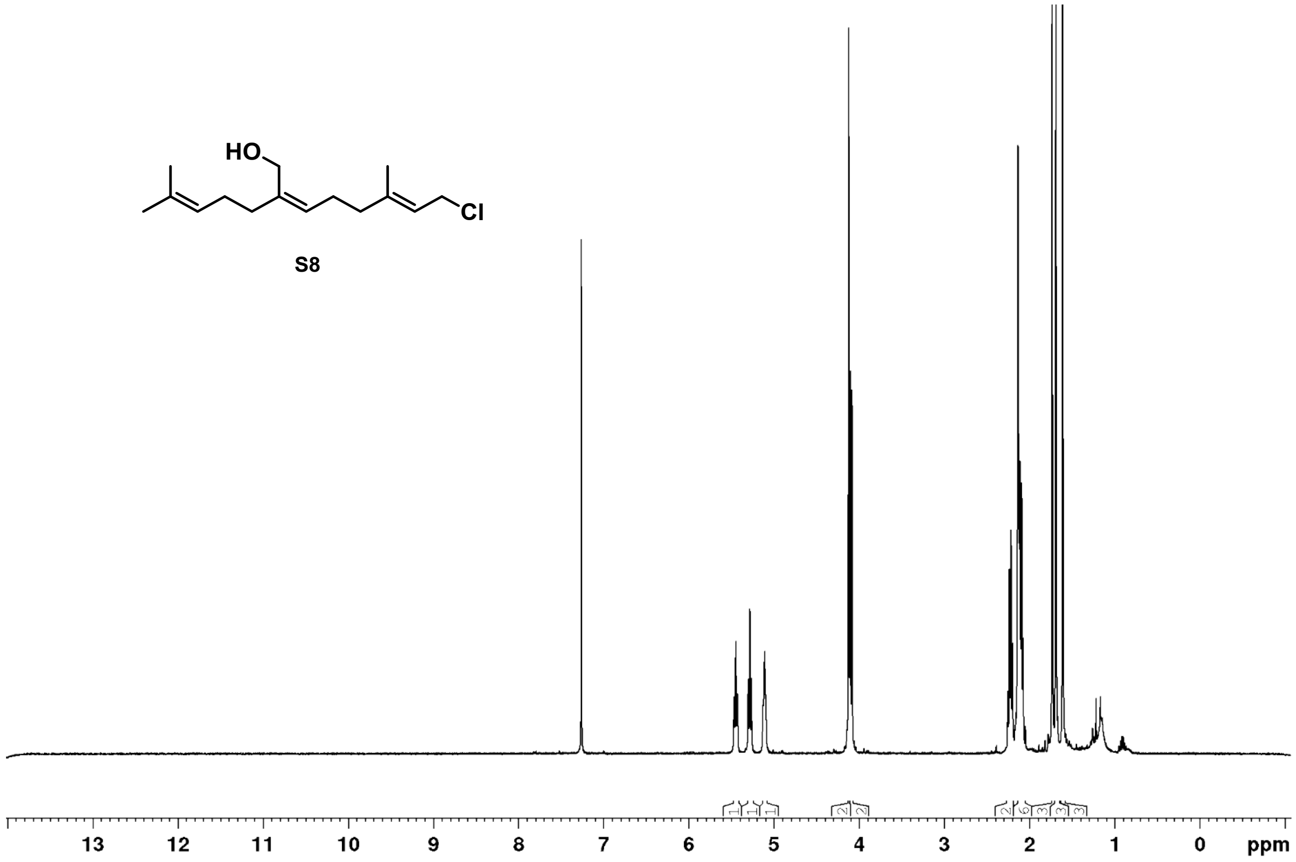
HO
Cl
S8
13 12 11 10 9 8 7 6 5 4 3 2 1 0 ppm

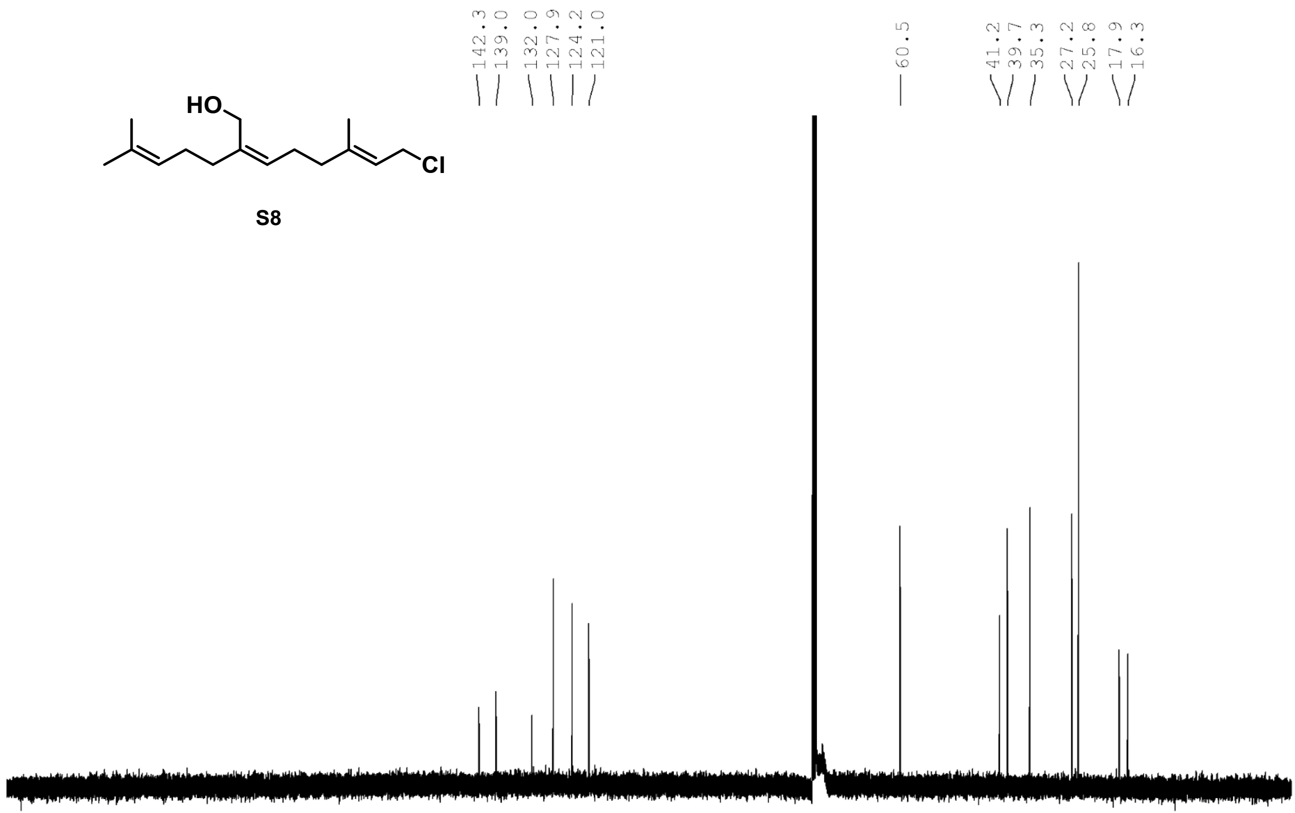
142.3
139.0
132.0
127.9
124.2
121.0
60.5
41.2
39.7
35.3
27.2
25.8
17.9
16.3
HO
Cl
S8

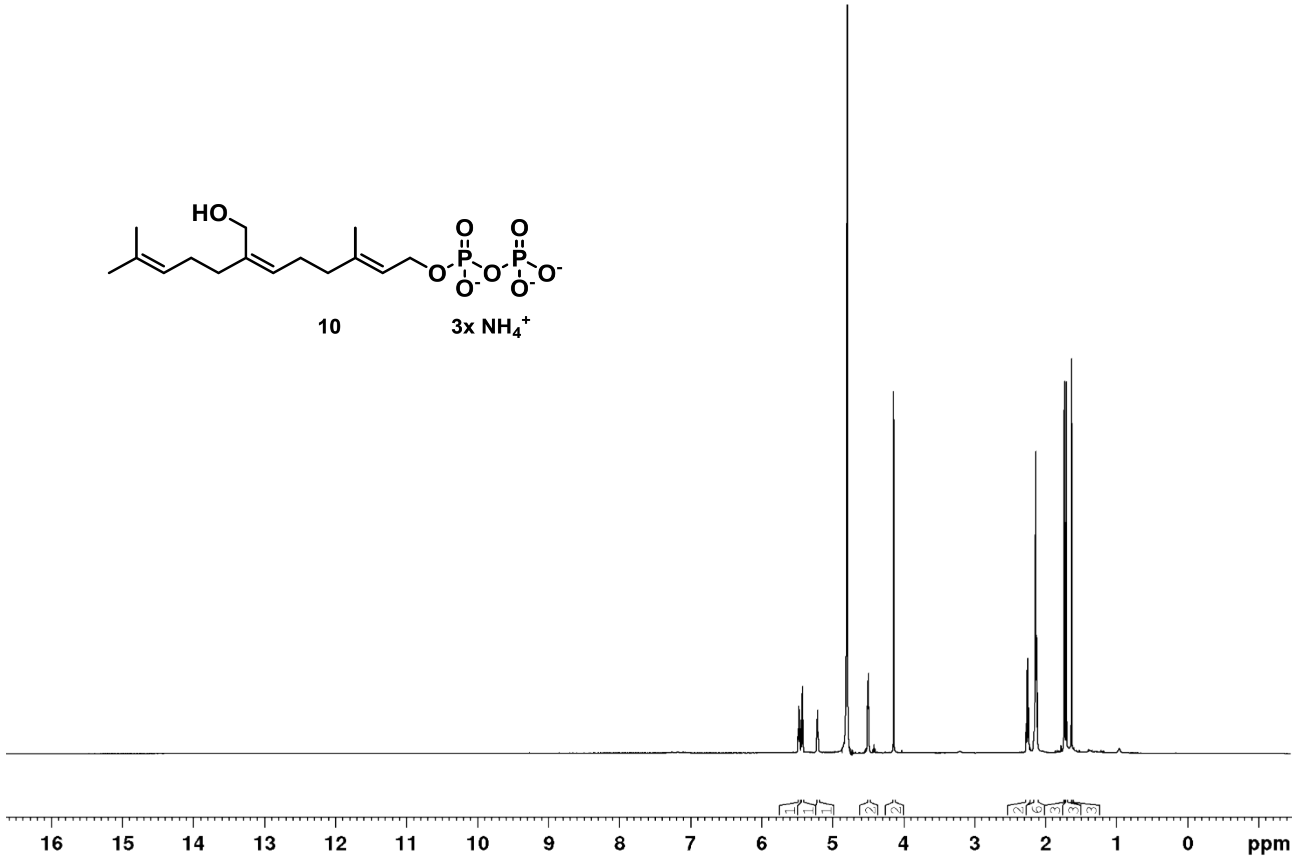
10
3x NH4+
16 15 14 13 12 11 10 9 8 7 6 5 4 3 2 1 0 ppm

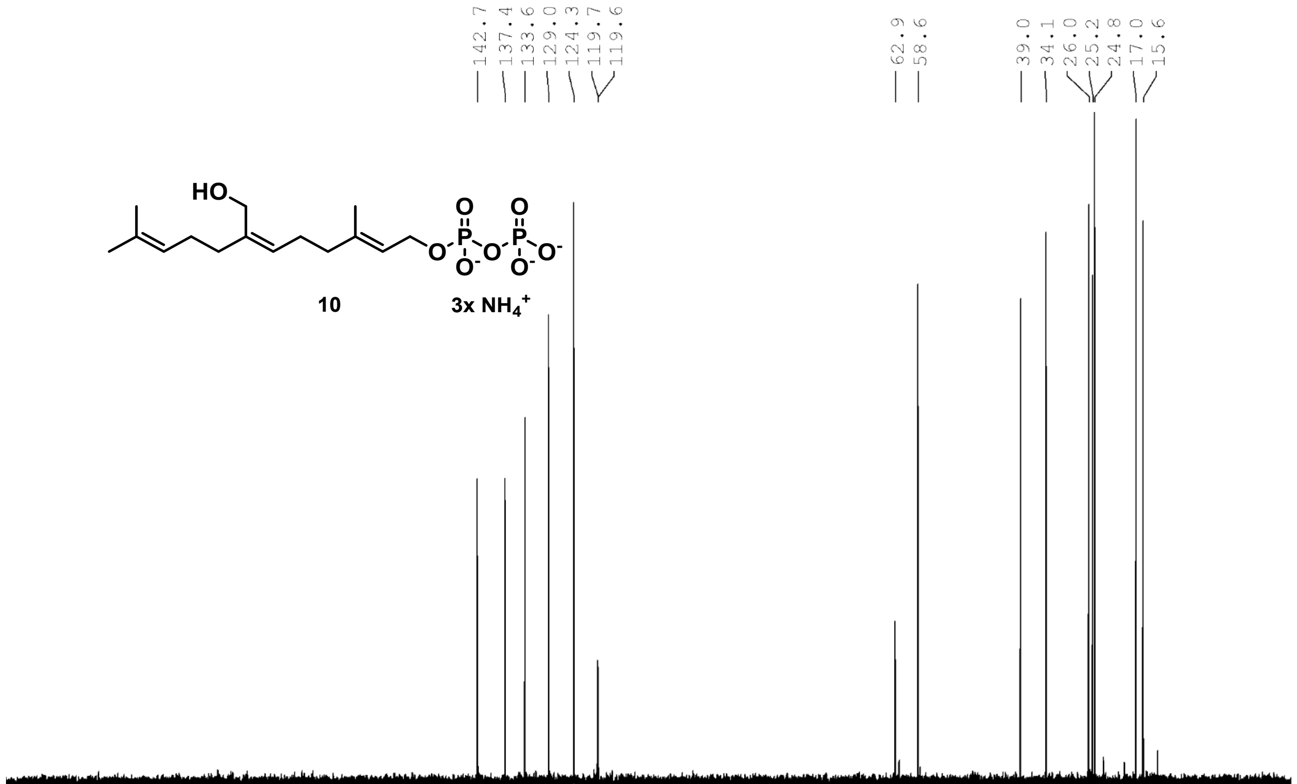
142.7
137.4
133.6
129.0
124.3
119.7
119.6
62.9
58.6
39.0
34.1
26.0
25.2
24.8
17.0
15.6
10
3x NH4+

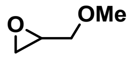

S9

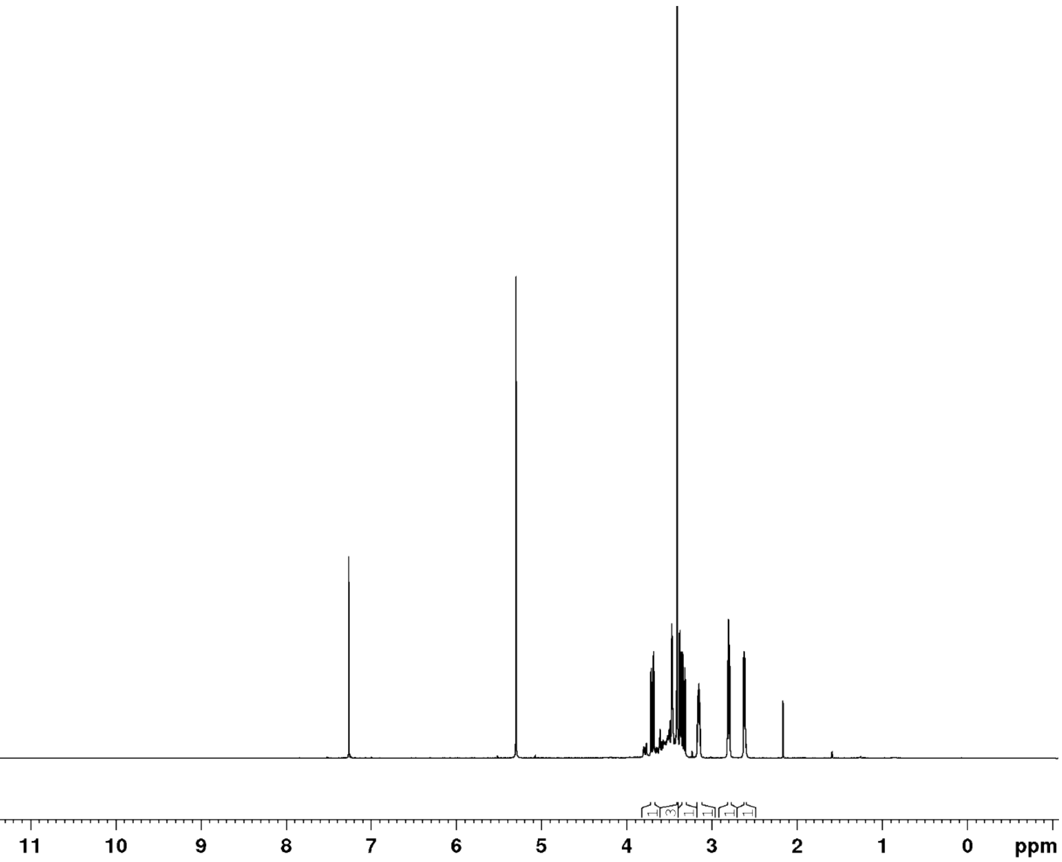

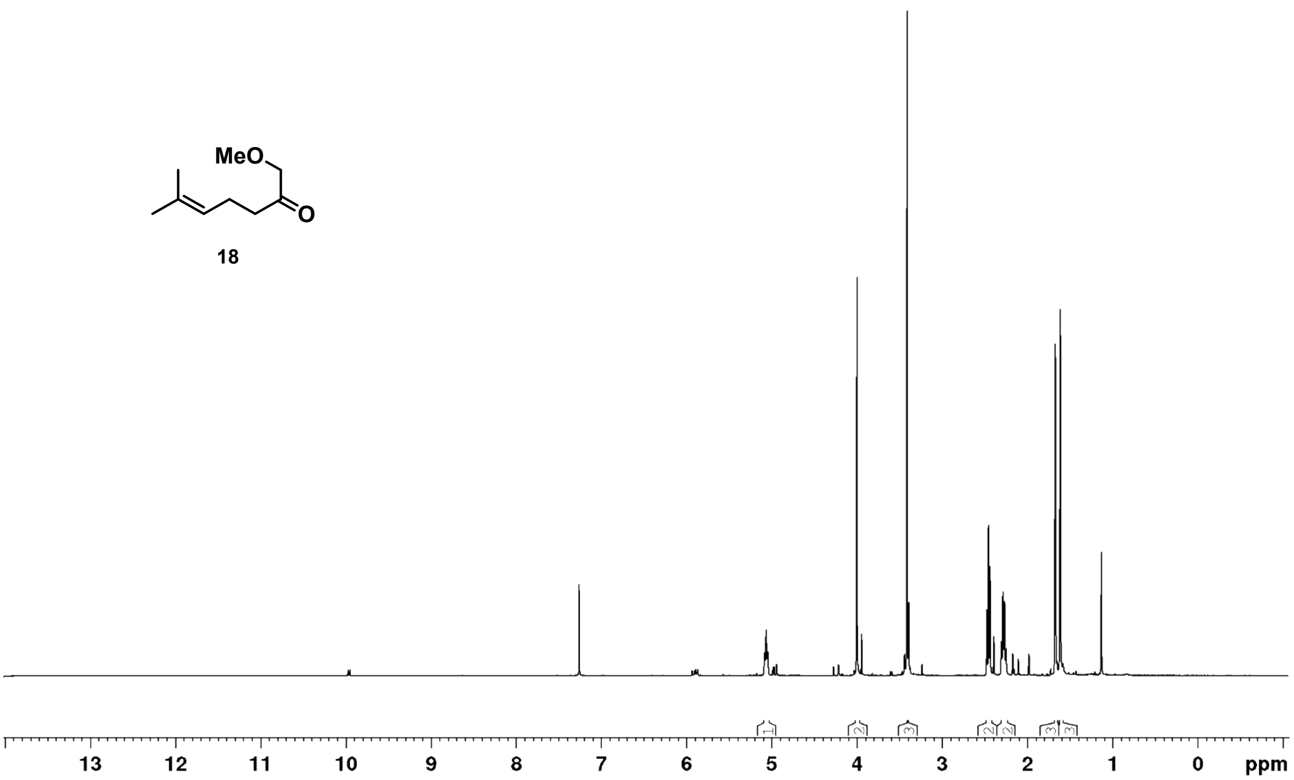
MeO
O
18
13
12
11
10
9
8
7
6
5
4
3
2
1
0
ppm

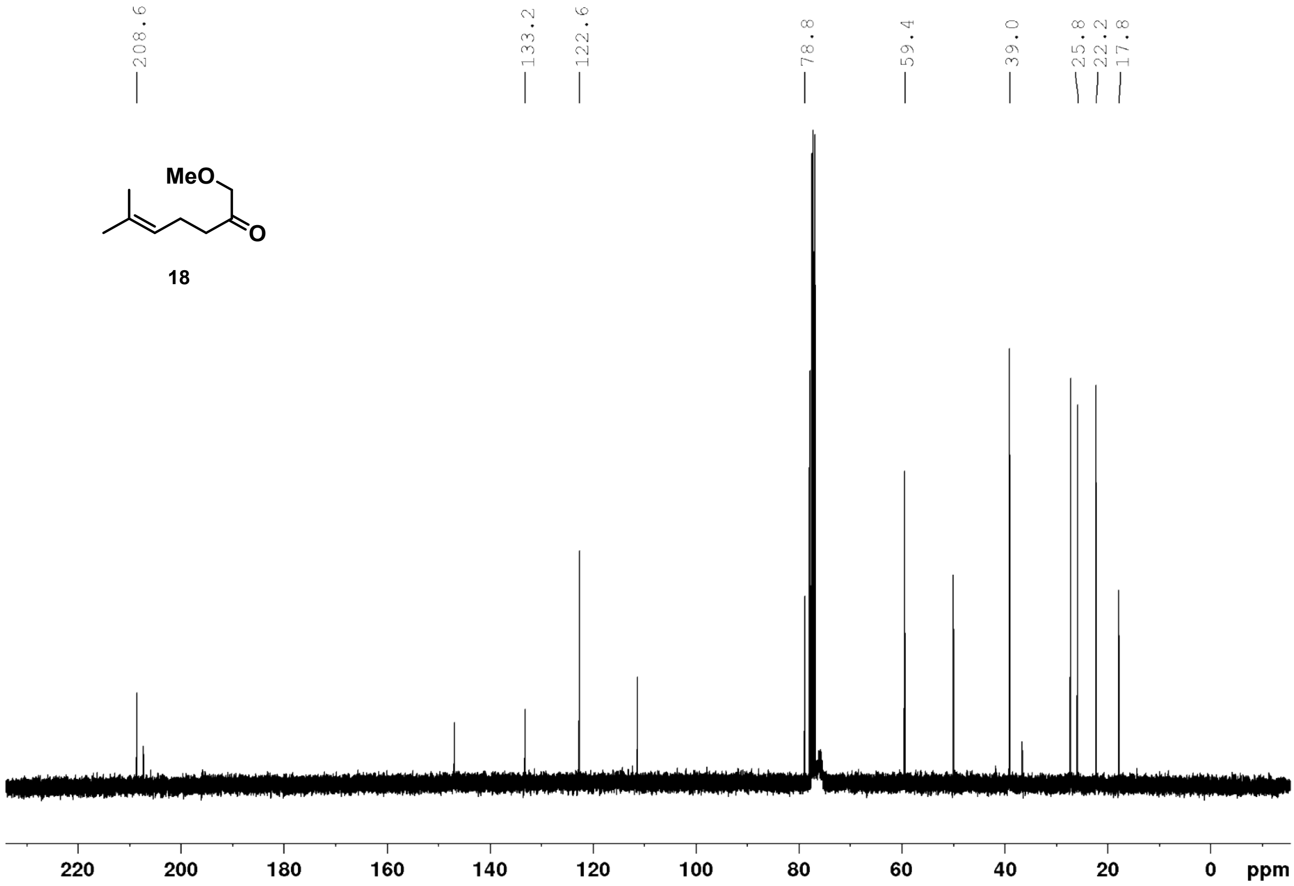
208.6
133.2
122.6
78.8
59.4
39.0
25.8
22.2
17.8
MeO
O
18
220
200
180
160
140
120
100
80
60
40
20
0
ppm

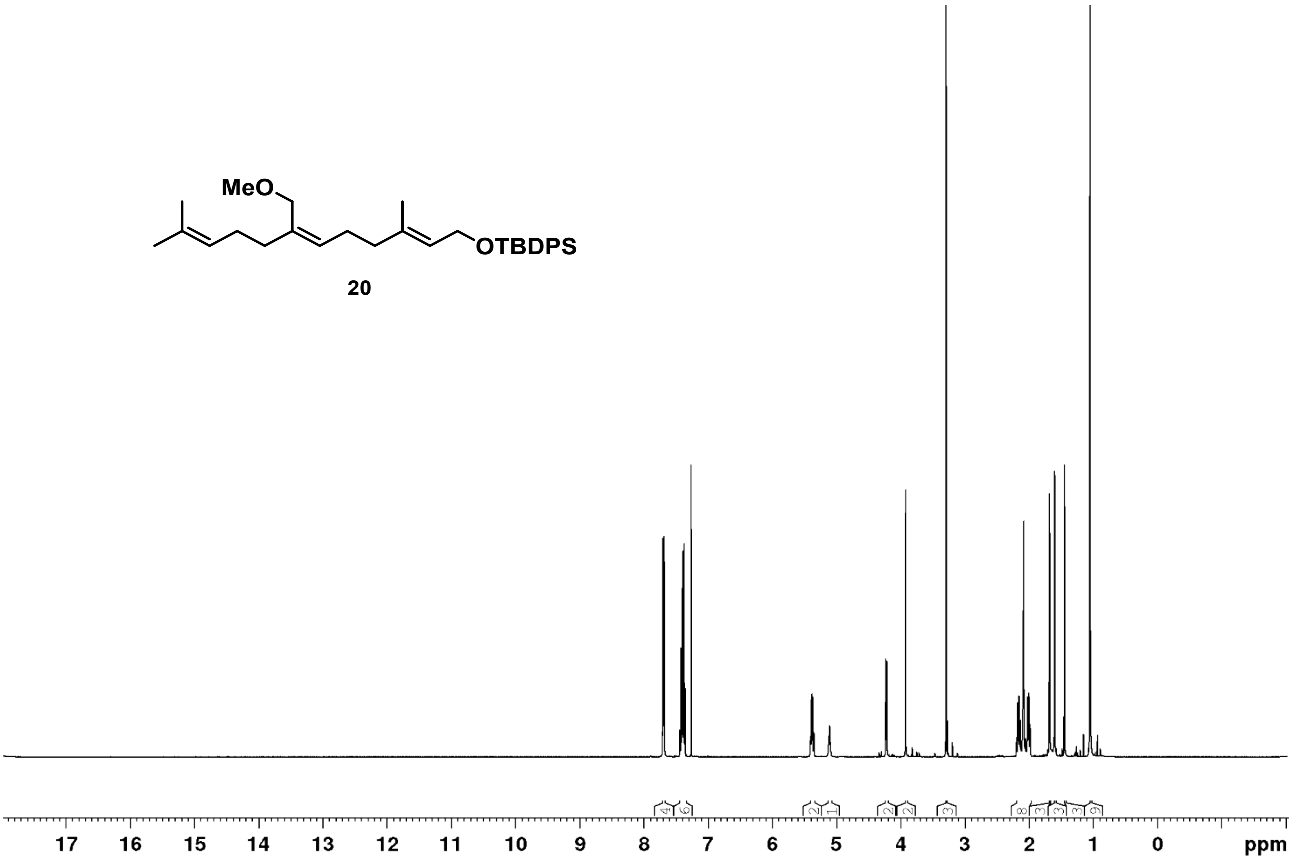
MeO
OTBDPS
20
4 6 2 1 2 2 3 8 3 3 3 9
17 16 15 14 13 12 11 10 9 8 7 6 5 4 3 2 1 0 ppm

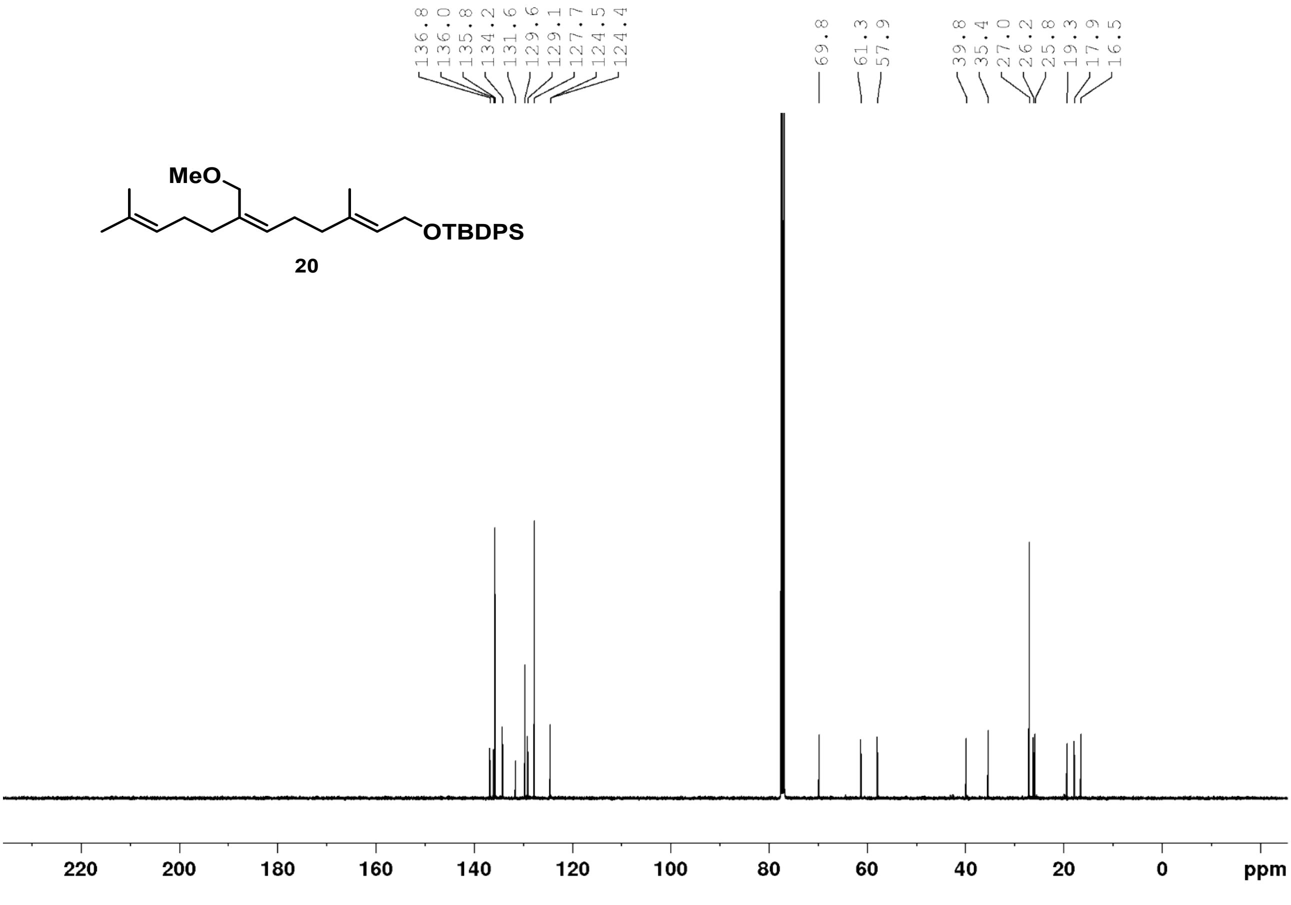
136.8 136.0 135.8 134.2 131.6 129.6 129.1 127.7 124.5 124.4
69.8 61.3 57.9
39.8 35.4 27.0 26.2 25.8 19.3 17.9 16.5
MeO
OTBDPS
20
220 200 180 160 140 120 100 80 60 40 20 0 ppm

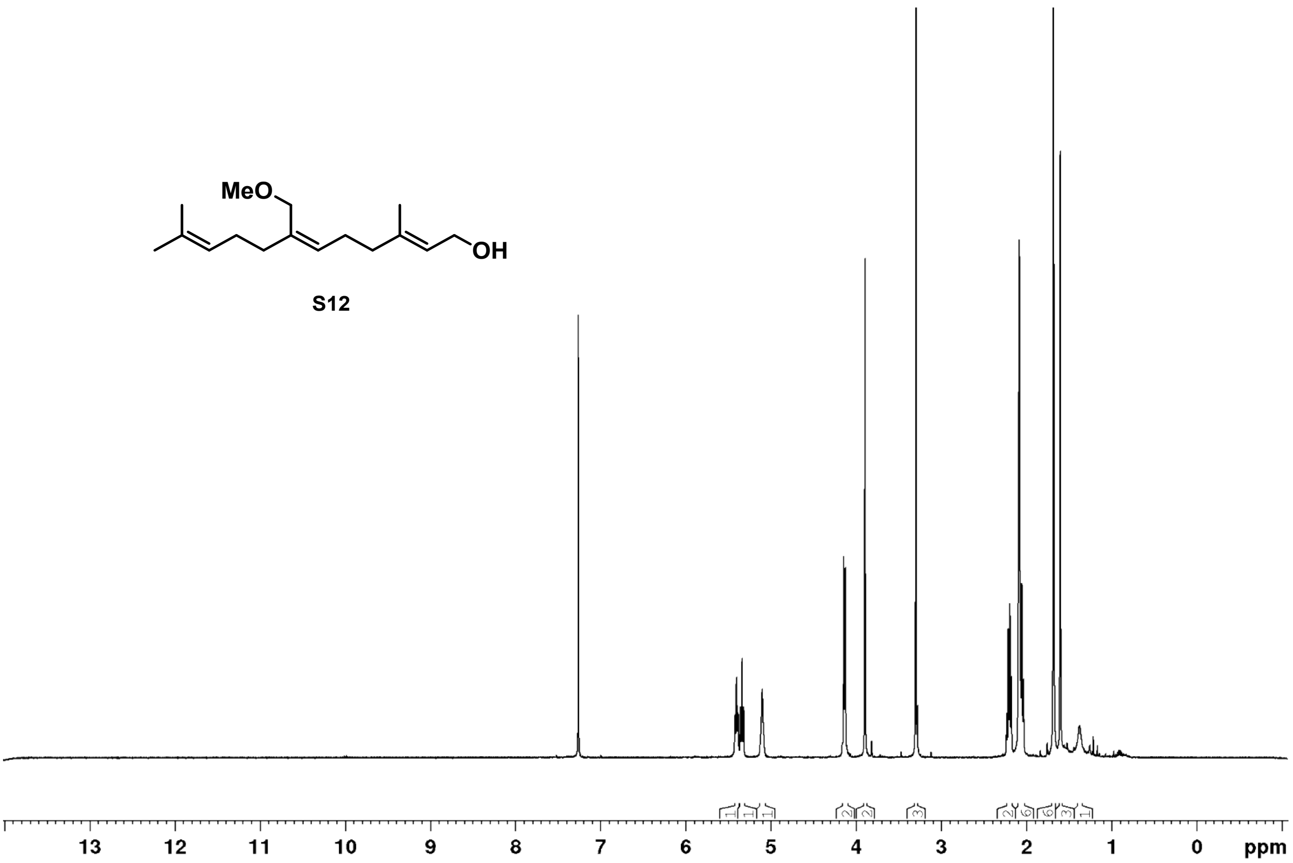
MeO
OH
S12
13 12 11 10 9 8 7 6 5 4 3 2 1 0 ppm

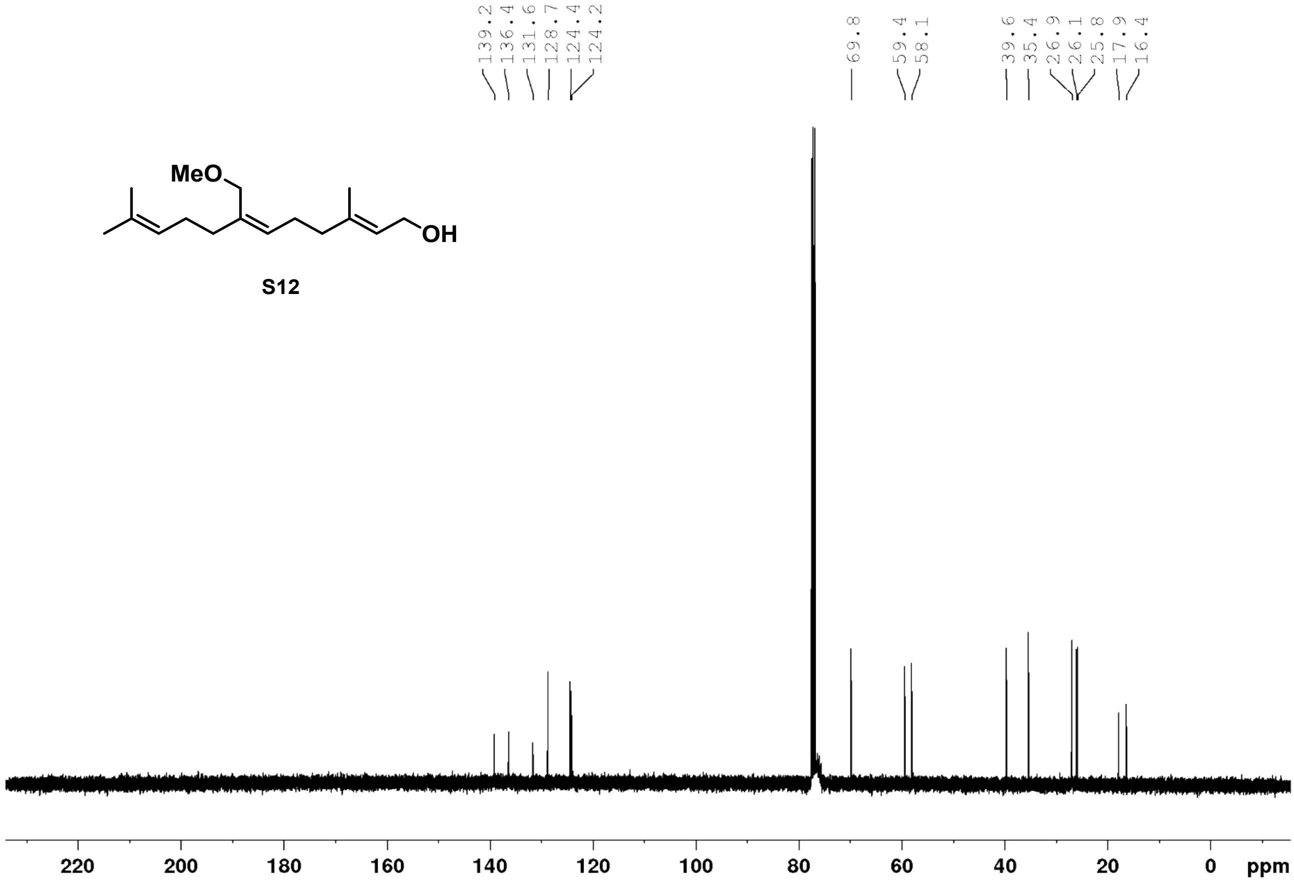
139.2
136.4
131.6
128.7
124.4
124.2
69.8
59.4
58.1
39.6
35.4
26.9
26.1
25.8
17.9
16.4
MeO
OH
S12
220 200 180 160 140 120 100 80 60 40 20 0 ppm

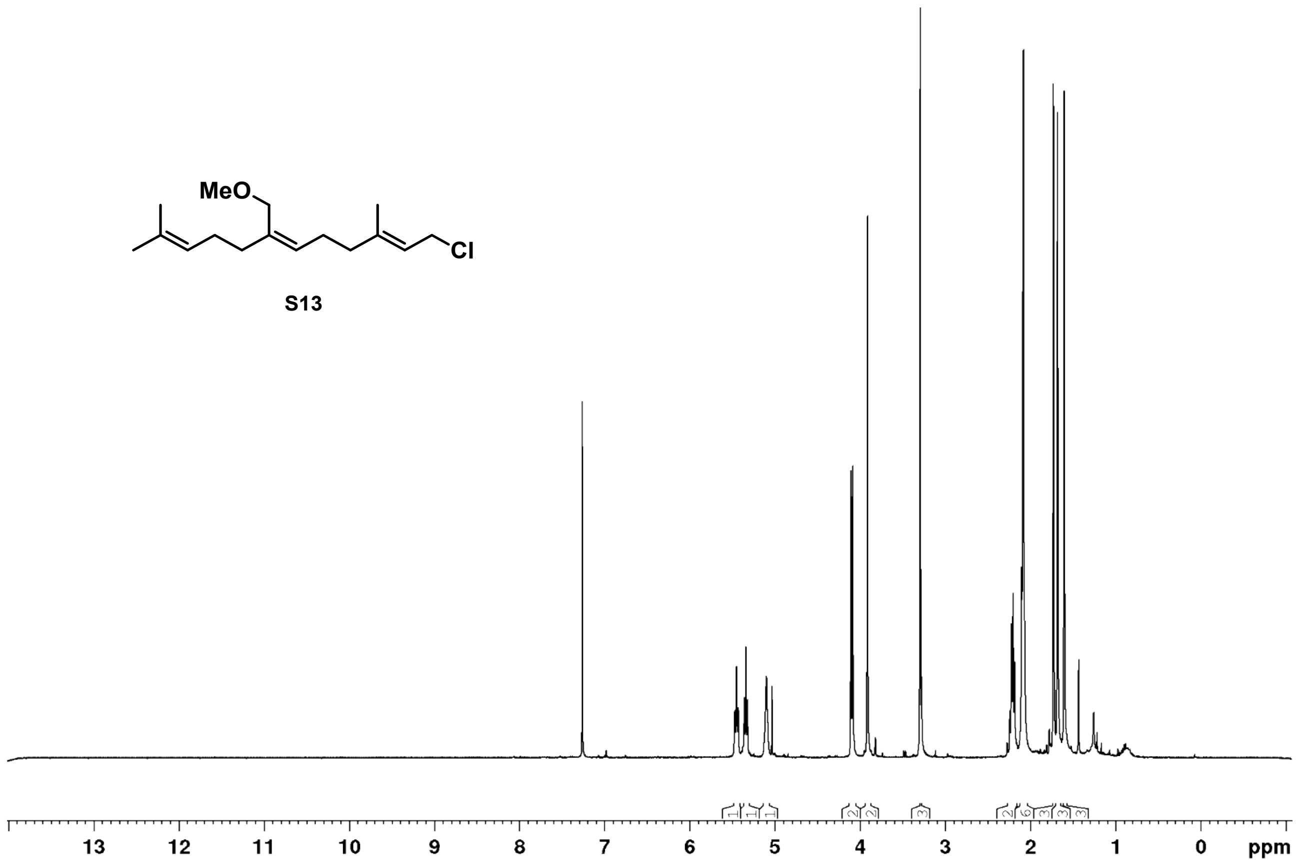
MeO
Cl
S13
13
12
11
10
9
8
7
6
5
4
3
2
1
0
ppm

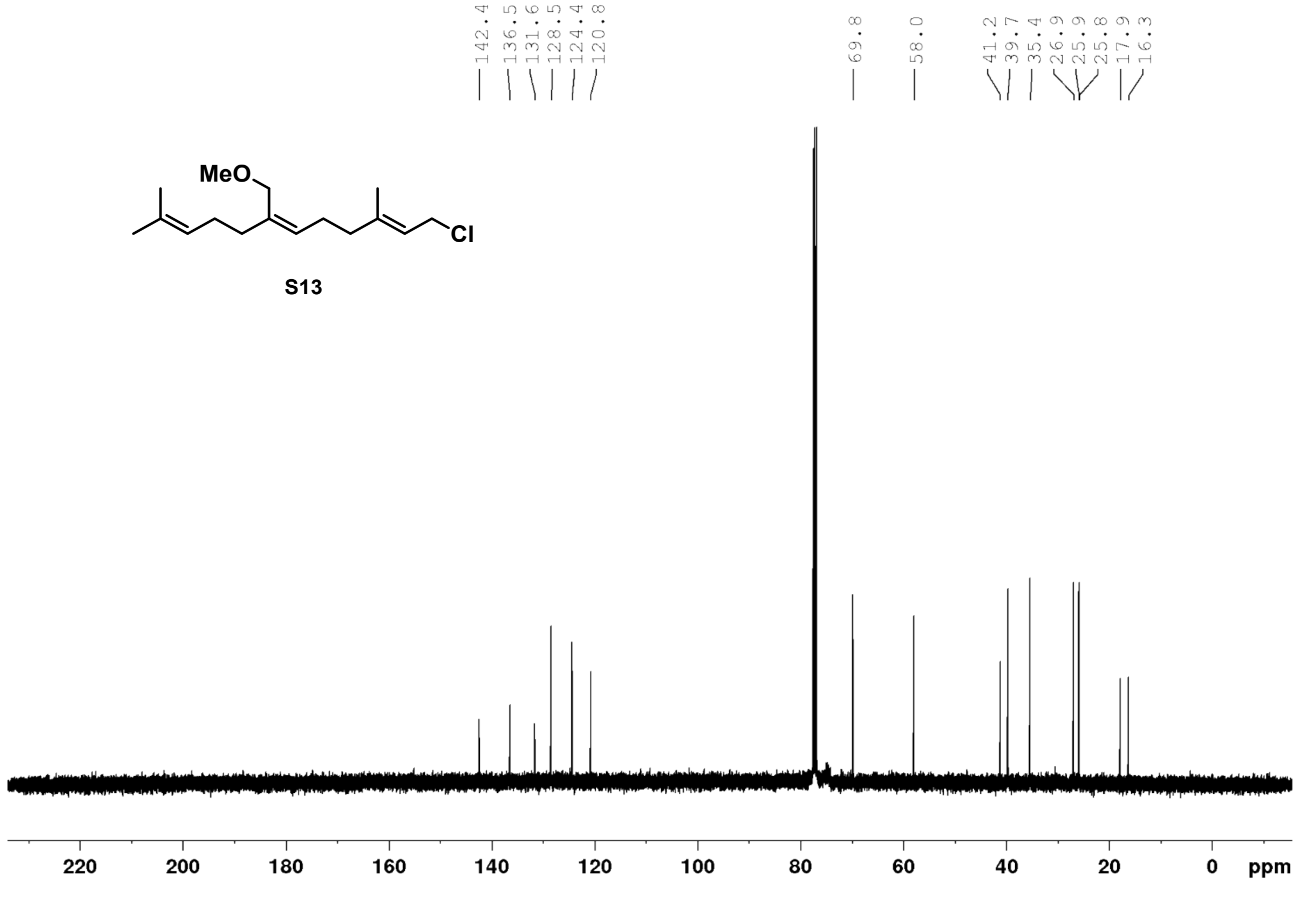
142.4
136.5
131.6
128.5
124.4
120.8
69.8
58.0
41.2
39.7
35.4
26.9
25.9
25.8
17.9
16.3
MeO
Cl
S13
220
200
180
160
140
120
100
80
60
40
20
0
ppm

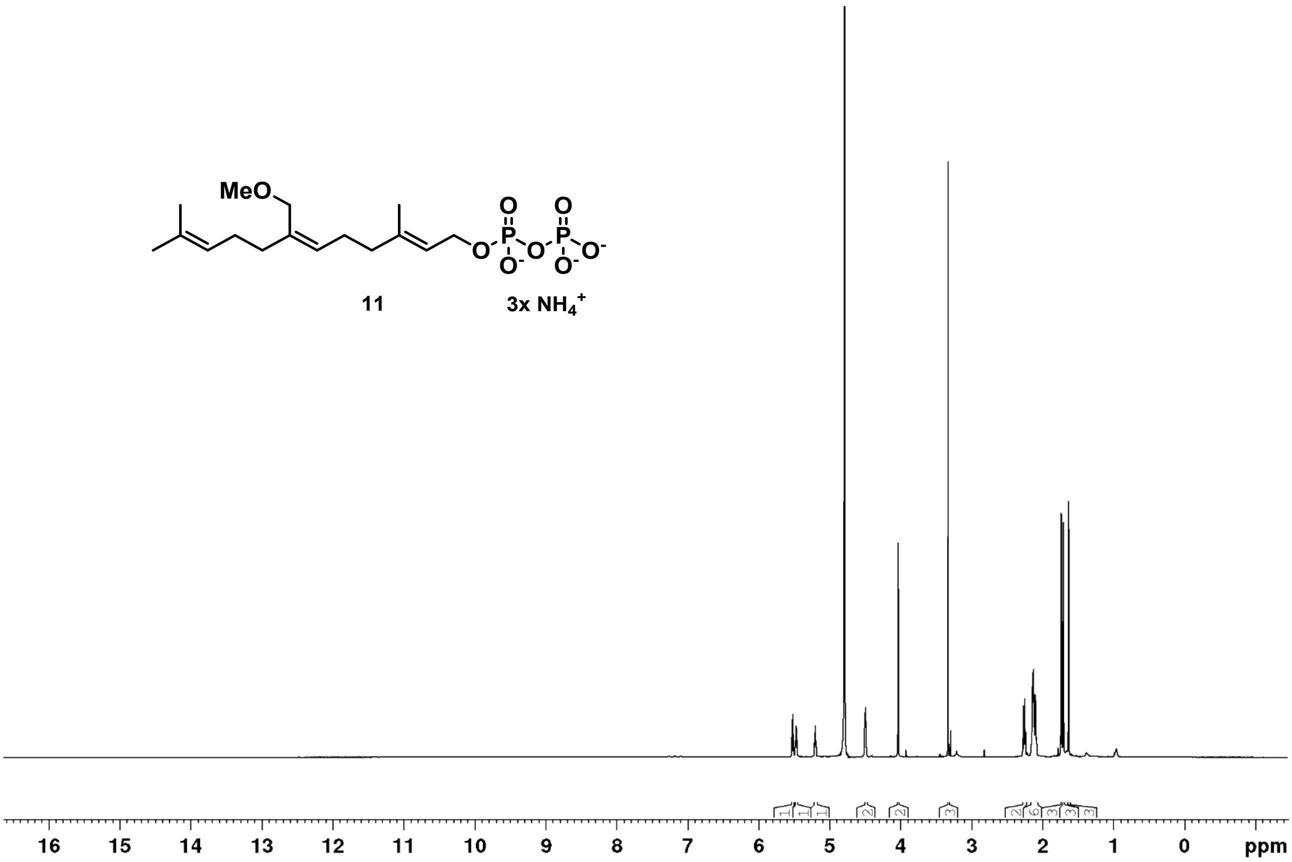
MeO
11
3x NH_4^+
16 15 14 13 12 11 10 9 8 7 6 5 4 3 2 1 0 ppm

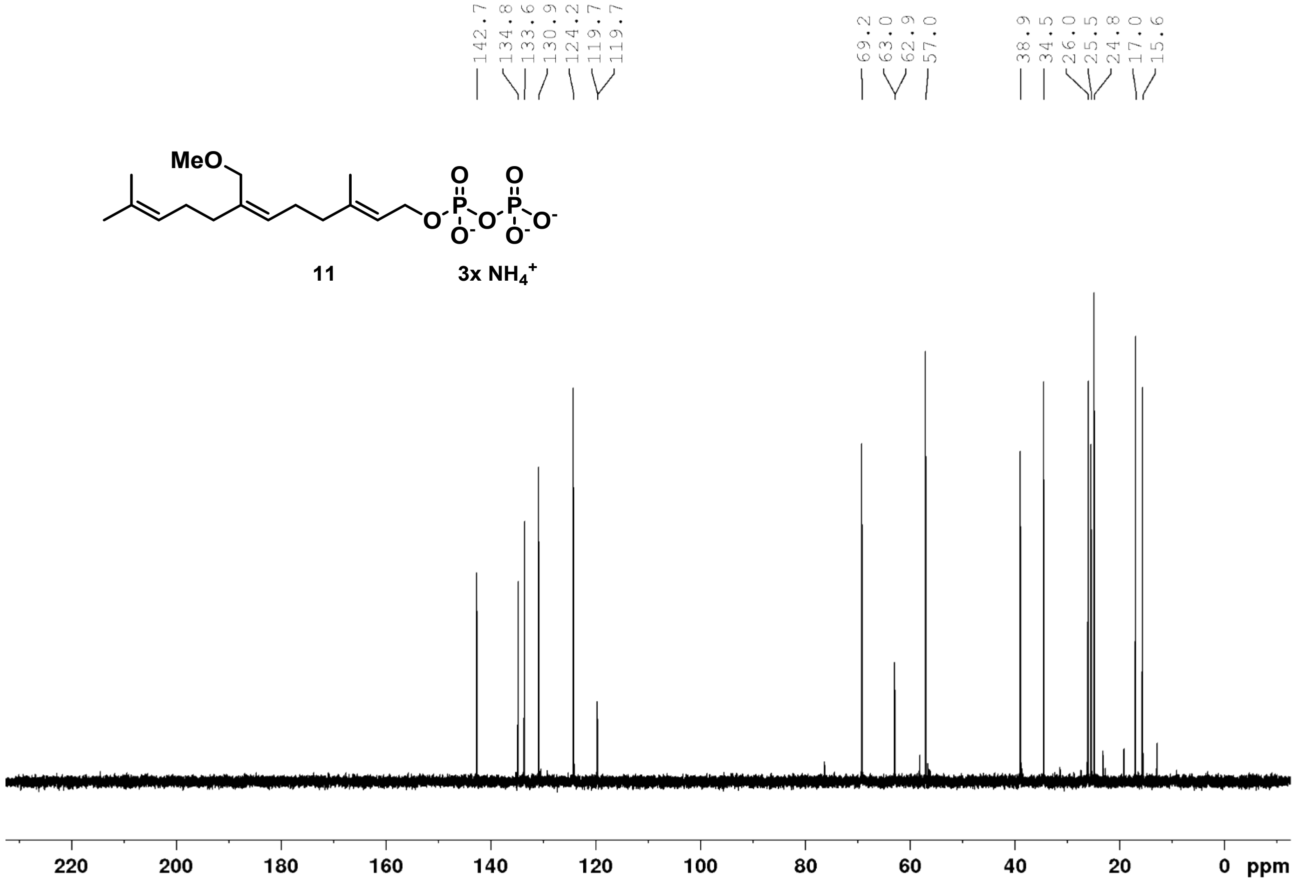
142.7
134.8
133.6
130.9
124.2
119.7
119.7
69.2
63.0
62.9
57.0
38.9
34.5
26.0
25.5
24.8
17.0
15.6
MeO
11
3x NH_4^+
220 200 180 160 140 120 100 80 60 40 20 0 ppm

C. Copies of ^{31}P-NMR spectra (chemical synthesis)

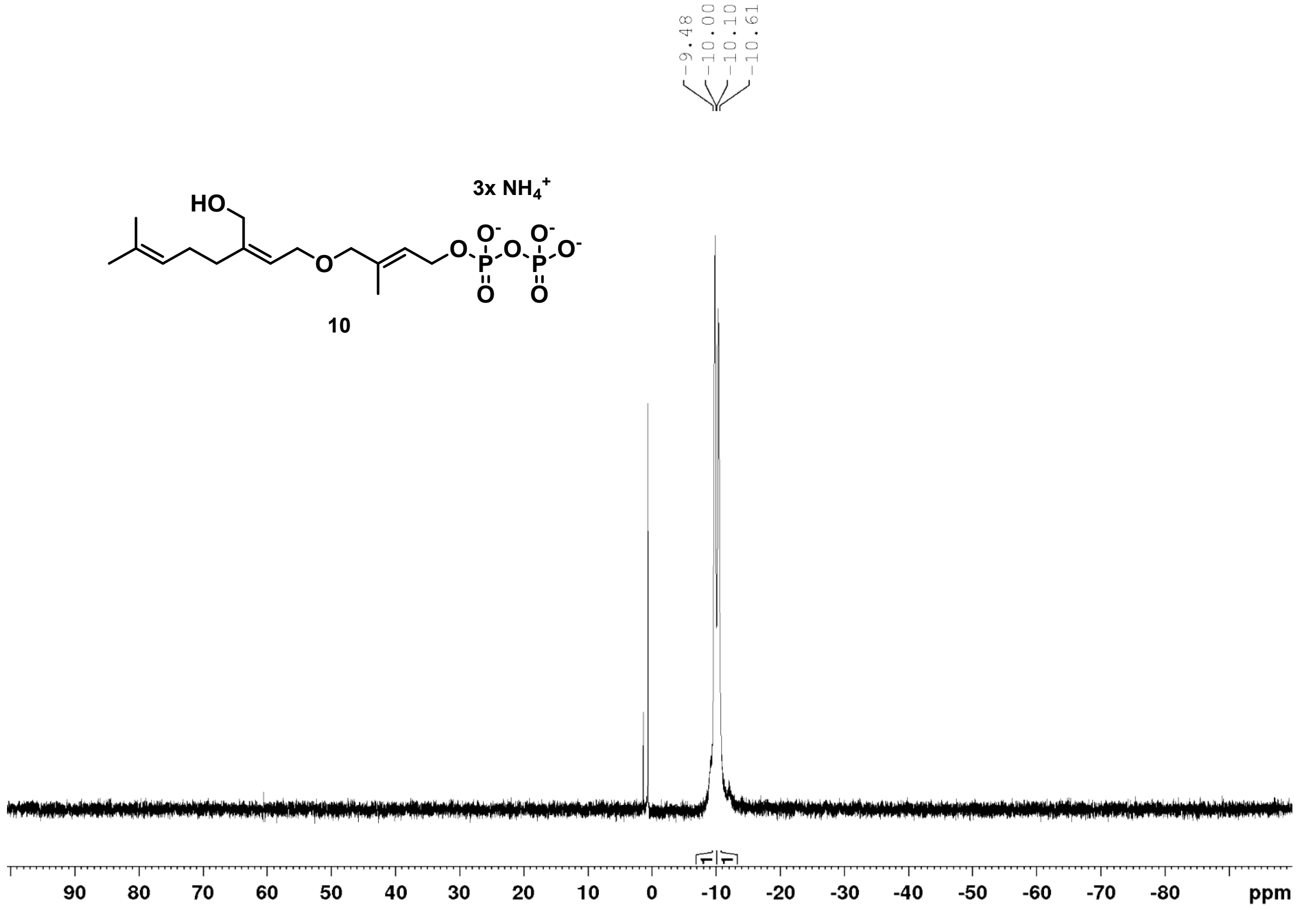

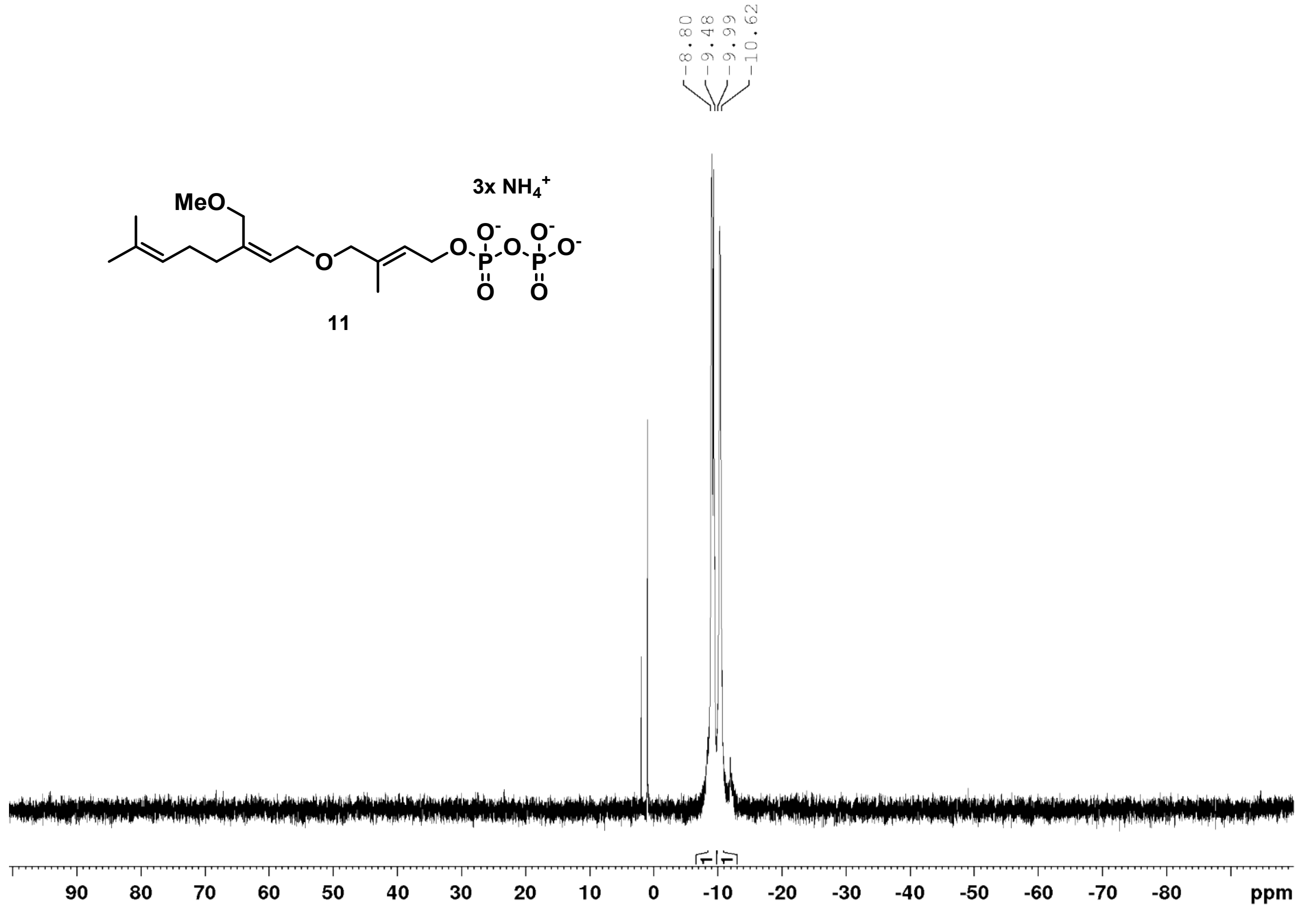

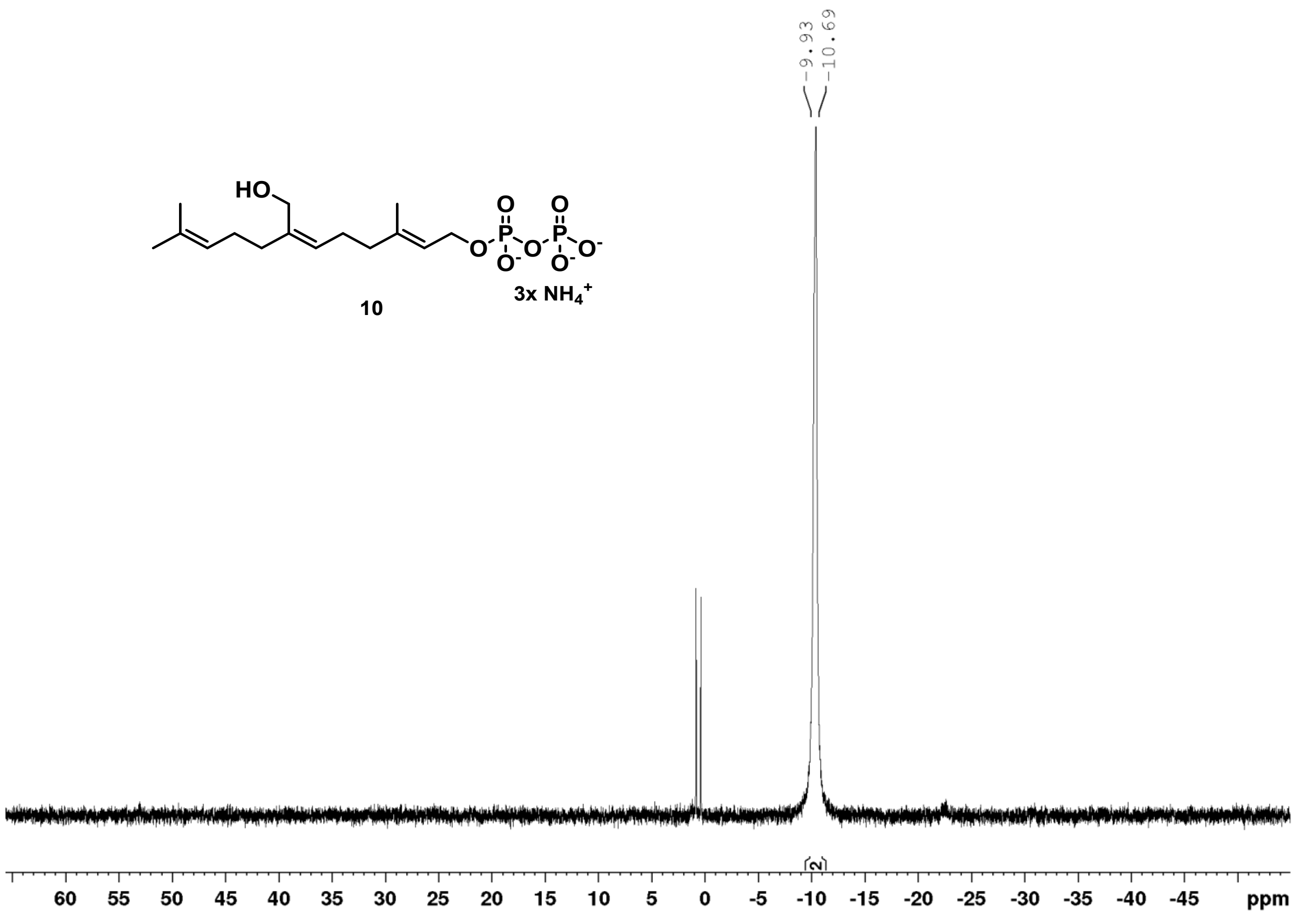
-9.93
-10.69
HO
O
O
P
P
O
O
O
O-
O-
O-
3x NH4+
10
60 55 50 45 40 35 30 25 20 15 10 5 0 -5 -10 -15 -20 -25 -30 -35 -40 -45 ppm

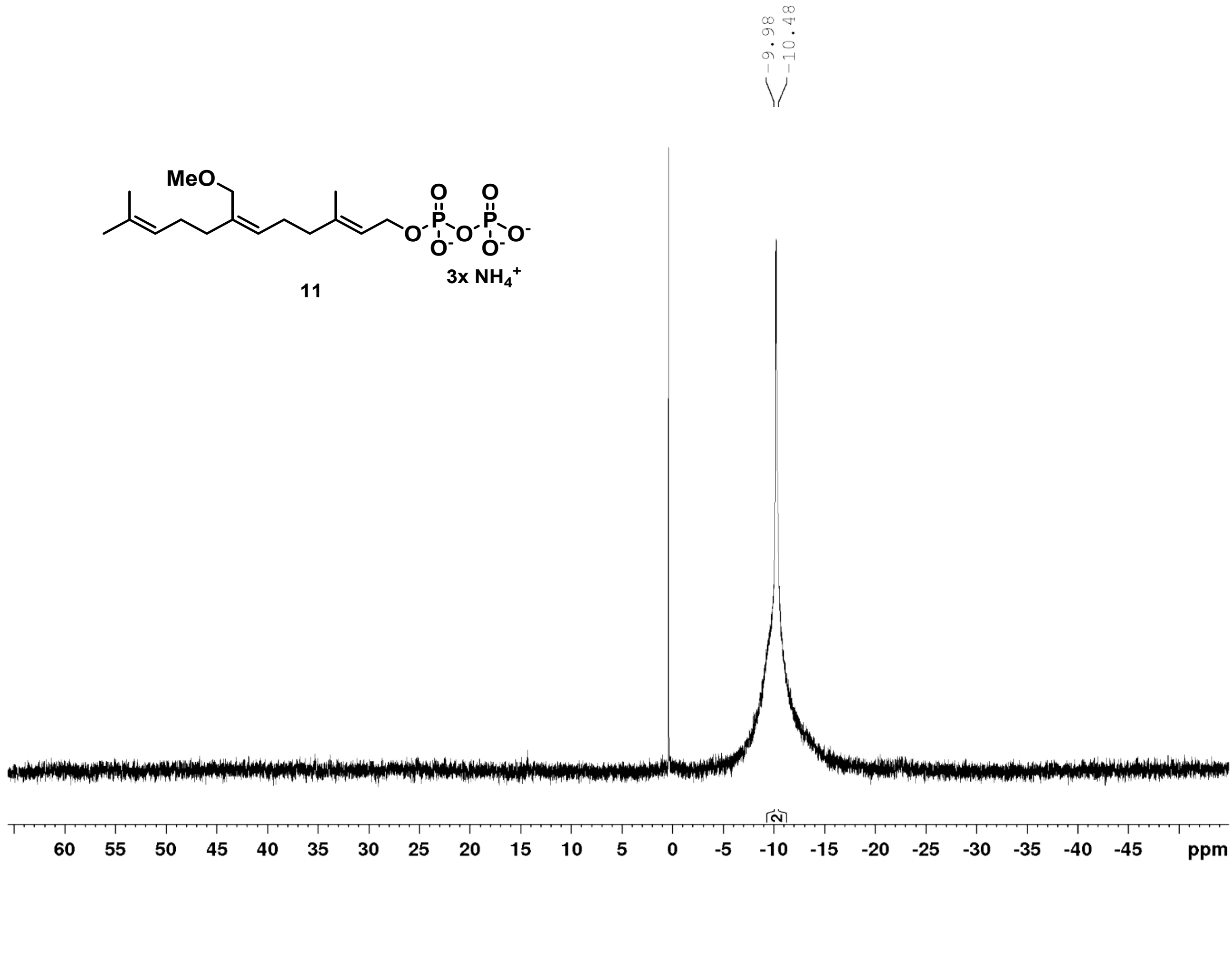
-9.98
-10.48
MeO
O
O
P
P
O
O
O
O-
O-
O-
3x NH4+
11
60 55 50 45 40 35 30 25 20 15 10 5 0 -5 -10 -15 -20 -25 -30 -35 -40 -45 ppm
